# Supplementary material for: Genome-Wide Identification and Evolutionary Analyses of SrfA Operon Genes in Bacillus
Source: Genes (Basel). 2023 Feb 7;14(2):422. doi: 10.3390/genes14020422 (PMC9956979; doi:10.3390/genes14020422)
Supplement: Supplementary file 1 [file genes-14-00422-s001.zip › Supplementary Materials.pdf]

Supporting information:

Figure S1. Detailed phylogenetic tree of *SrfAA*, *SrfAB* and *SrfAC* genes in OG0000006.

Figure S2. Detailed phylogenetic tree of *SrfAA*, *SrfAB* and *SrfAC* genes in OG0000009.

Figure S3. Detailed phylogenetic tree of *SrfAA*, *SrfAB* and *SrfAC* genes in OG0000249.

Figure S4. Detailed phylogenetic tree of *SrfAA*, *SrfAB* and *SrfAC* genes in OG0000642.

Figure S5. Detailed phylogenetic tree of *SrfAA*, *SrfAB* and *SrfAC* genes in OG0000675.

Figure S6. Detailed phylogenetic tree of *SrfAA*, *SrfAB* and *SrfAC* genes in OG0000736.

Figure S7. Detailed phylogenetic tree of *SrfAA*, *SrfAB* and *SrfAC* genes in OG0000753.

Figure S8. Detailed phylogenetic tree of *SrfAA*, *SrfAB* and *SrfAC* genes in OG0000878.

Figure S9. Detailed phylogenetic tree of *SrfAA*, *SrfAB* and *SrfAC* genes in OG0000898.

Figure S10. Detailed phylogenetic tree of *SrfAA*, *SrfAB* and *SrfAC* genes in OG0001033.

Figure S11. Detailed phylogenetic tree of *SrfAA*, *SrfAB* and *SrfAC* genes in OG0001616.

Figure S12. Detailed phylogenetic tree of *SrfAA*, *SrfAB* and *SrfAC* genes in OG0002144.

Figure S13. Detailed phylogenetic tree of *SrfAA*, *SrfAB* and *SrfAC* genes in OG0002176.

Figure S14. Detailed phylogenetic tree of *SrfAA*, *SrfAB* and *SrfAC* genes in OG0002286.

Figure S15. Detailed phylogenetic tree of *SrfAA*, *SrfAB* and *SrfAC* genes in OG0003280.

Figure S16. Detailed phylogenetic tree of *SrfAA*, *SrfAB* and *SrfAC* genes in OG0003557.

Figure S17. Detailed phylogenetic tree of *SrfAA*, *SrfAB* and *SrfAC* genes in OG0004686.

Figure S18. Distribution of *SrfAA*, *SrfAB* and *SrfAC* genes in the *Bacillus subtilis* subsp. *subtilis* str. 168 (GCF_000009045) genome.

Figure S19. Distribution of *SrfAA*, *SrfAB* and *SrfAC* genes in the *Bacillus pseudomycoides* DSM 12442 (GCF_000161455) genome.

Figure S20. Distribution of *SrfAA*, *SrfAB* and *SrfAC* genes in the *Bacillus amyloliquefaciens* Y2 (GCF_000262385) genome.

Figure S21. Distribution of *SrfAA*, *SrfAB* and *SrfAC* genes in the *Bacillus sp*. Pc3 (GCF_000815145) genome.

Figure S22. Distribution of *SrfAA*, *SrfAB* and *SrfAC* genes in the *Bacillus velezensis* (GCF_003073255) genome.

Figure S23. Distribution of *SrfAA*, *SrfAB* and *SrfAC* genes in the *Bacillus velezensis* (GCF_003390415) genome.

Figure S24. Distribution of *SrfAA*, *SrfAB* and *SrfAC* genes in the *Bacillus amyloliquefaciens* (GCF_ 008824205) genome.

Figure S25. Distribution of *SrfAA*, *SrfAB* and *SrfAC* genes in the *Bacillus subtilis* (GCF_012647565) genome.

Figure S26. Distribution of *SrfAA*, *SrfAB* and *SrfAC* genes in the *Bacillus velezensis* (GCF_016904925) genome.

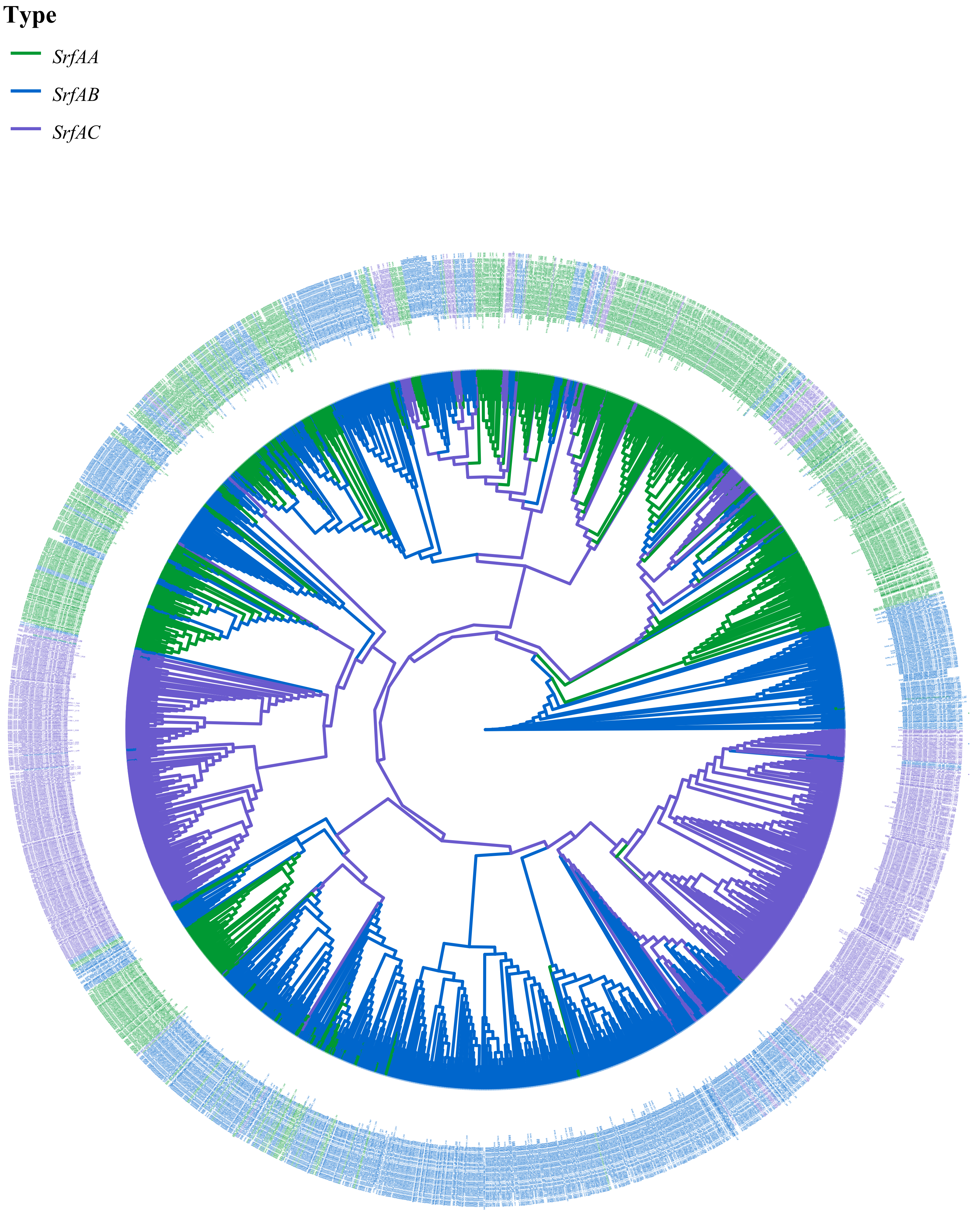

Figure S1. Detailed phylogenetic tree of *SrfAA*, *SrfAB* and *SrfAC* genes in OG0000006.

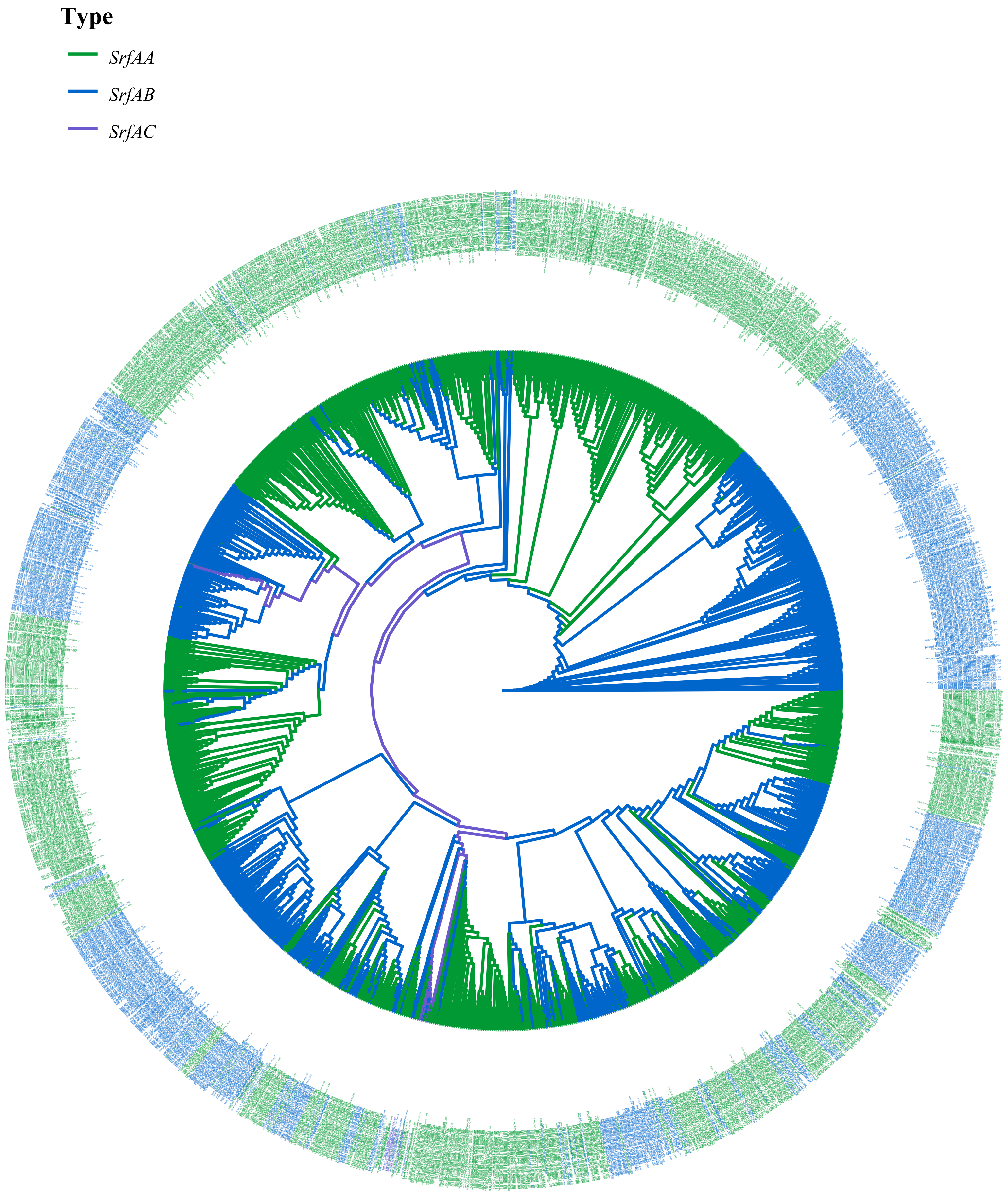

Figure S2. Detailed phylogenetic tree of *SrfAA*, *SrfAB* and *SrfAC* genes in OG0000009.

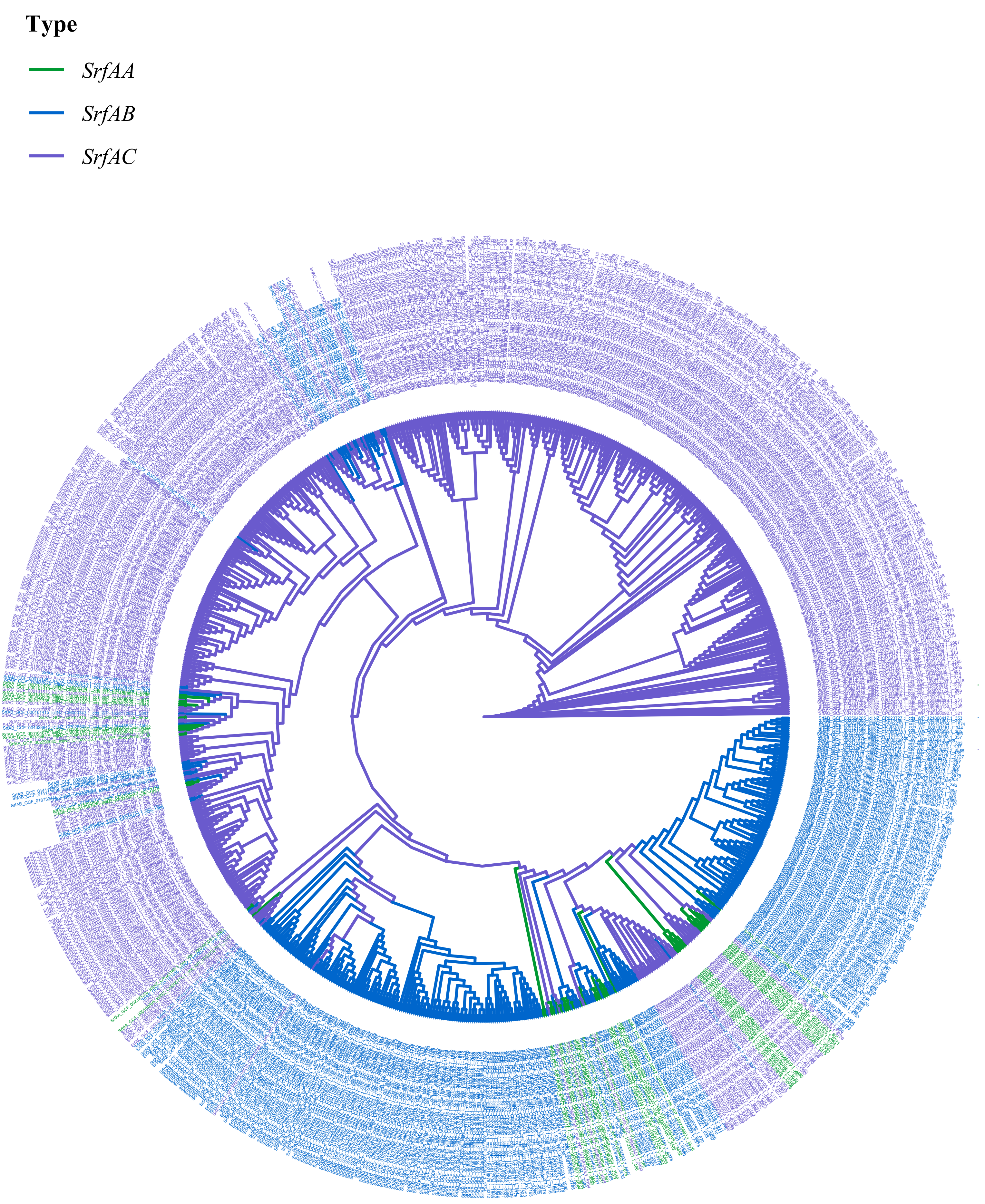

Figure S3. Detailed phylogenetic tree of *SrfAA*, *SrfAB* and *SrfAC* genes in OG0000249.

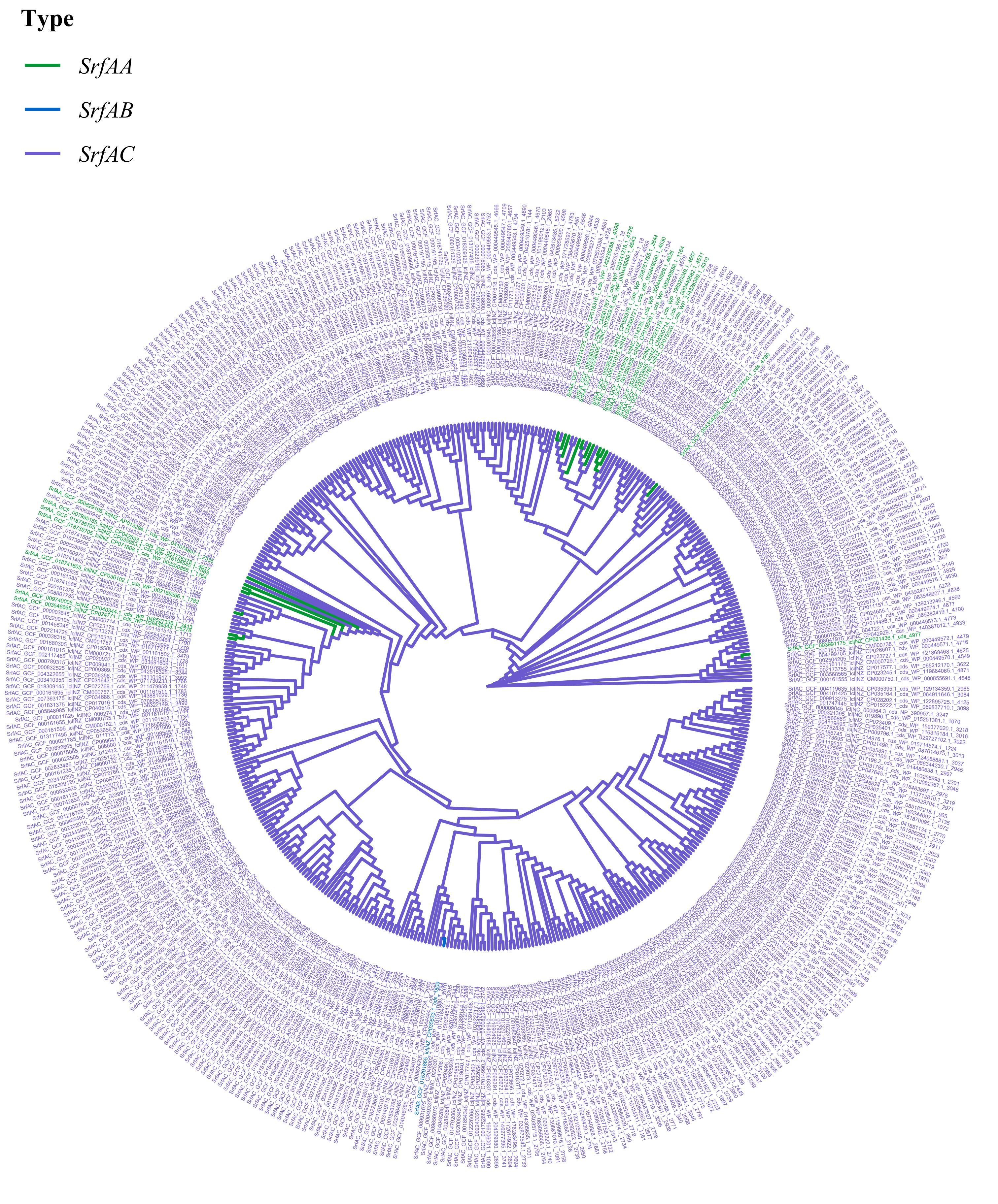

Figure S4. Detailed phylogenetic tree of *SrfAA*, *SrfAB* and *SrfAC* genes in OG0000642.

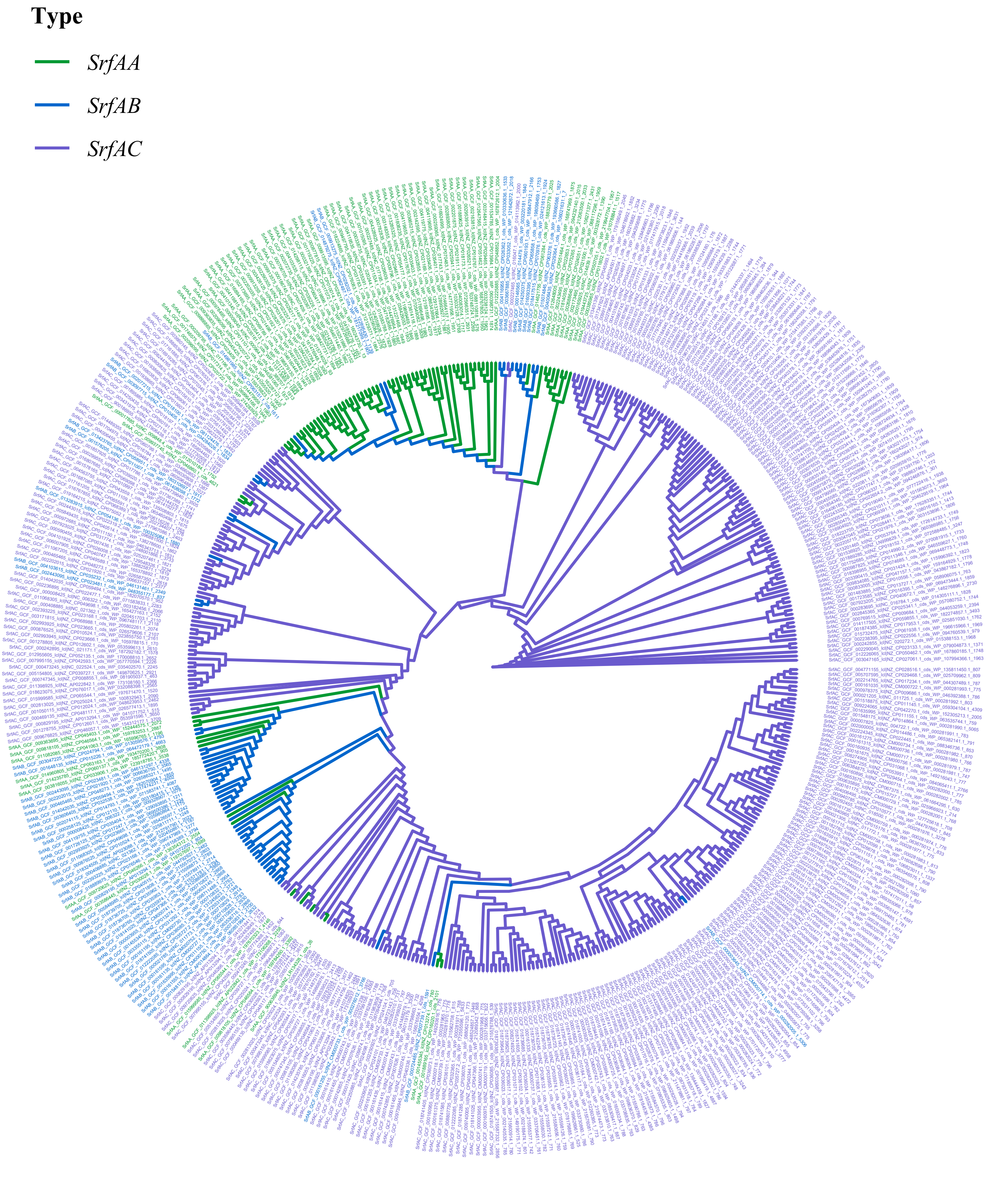

Figure S5. Detailed phylogenetic tree in of *SrfAA*, *SrfAB* and *SrfAC* genes in OG0000675.

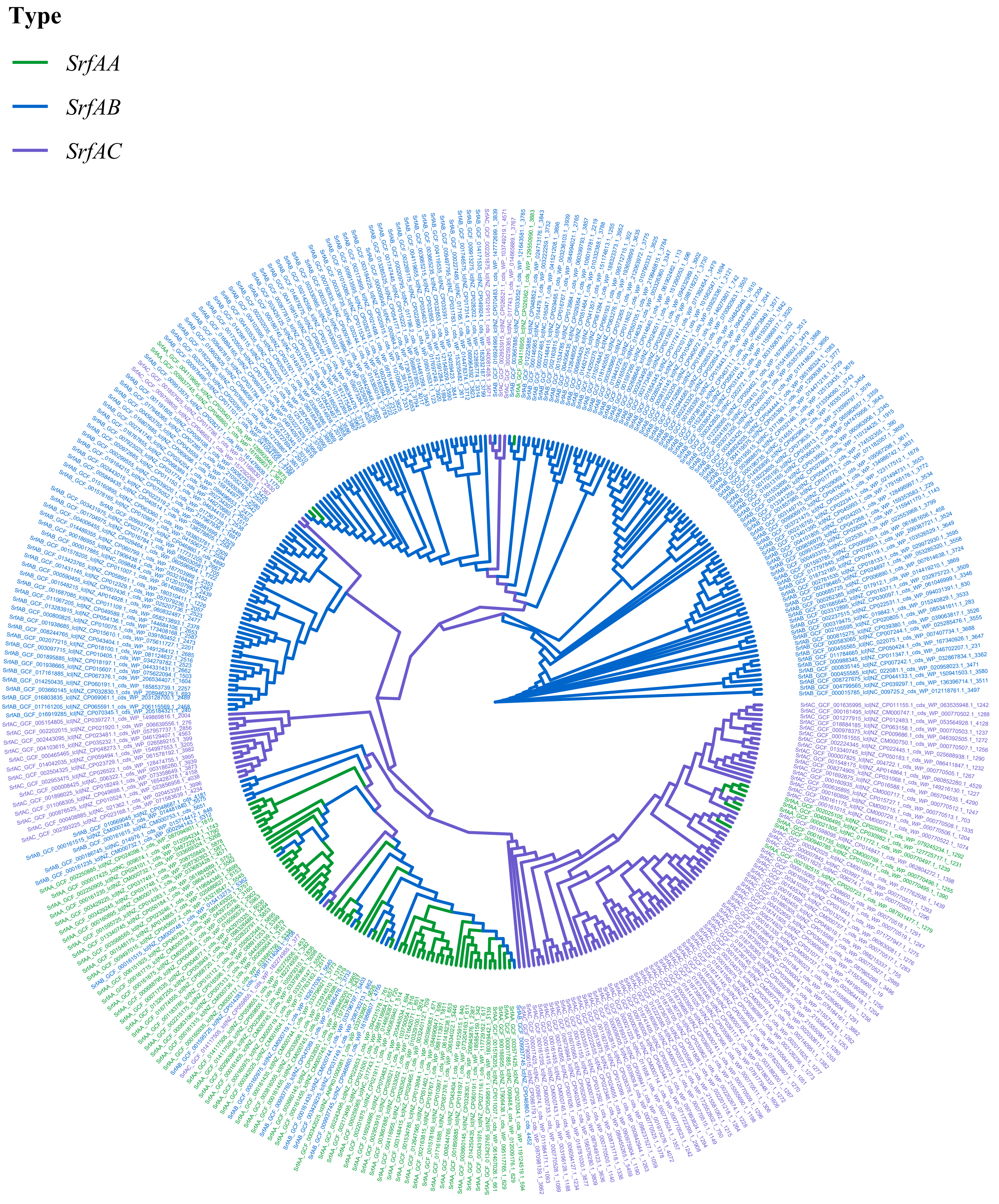

Figure S6. Detailed phylogenetic tree of *SrfAA*, *SrfAB* and *SrfAC* genes in OG0000736.

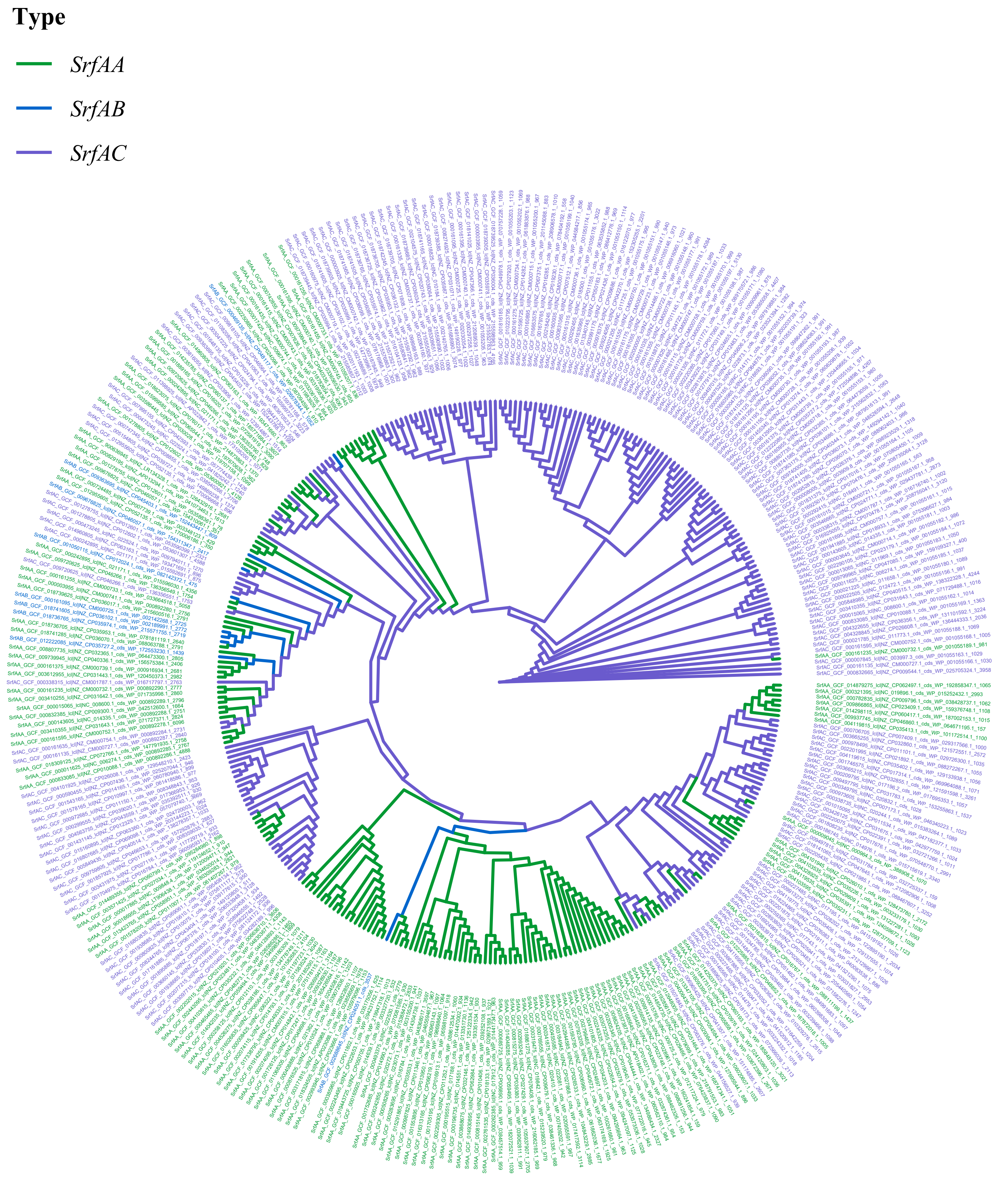

Figure S7. Detailed phylogenetic tree of *SrfAA*, *SrfAB* and *SrfAC* genes in OG0000753.

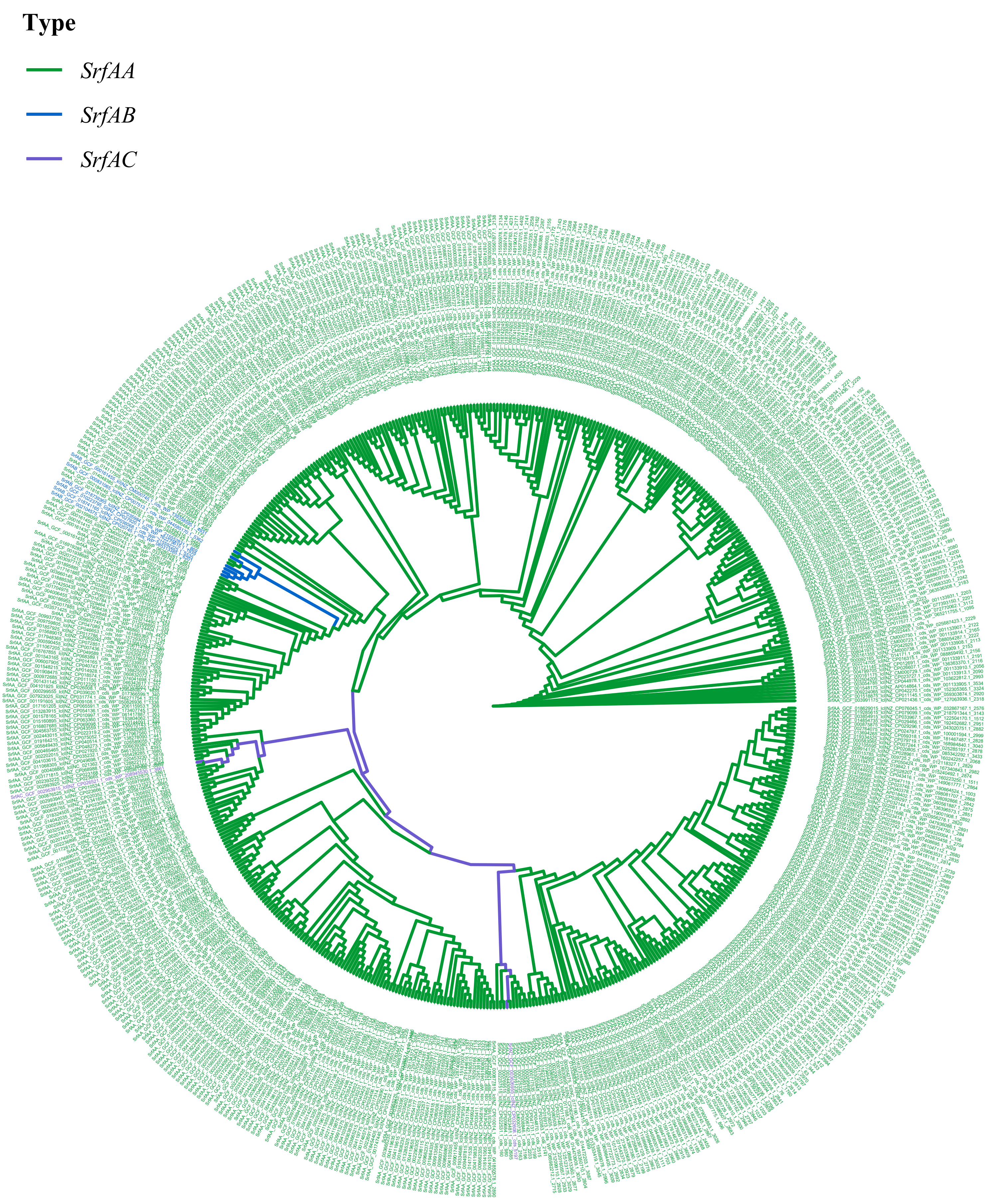

Figure S8. Detailed phylogenetic tree of *SrfAA*, *SrfAB* and *SrfAC* genes in OG0000878.

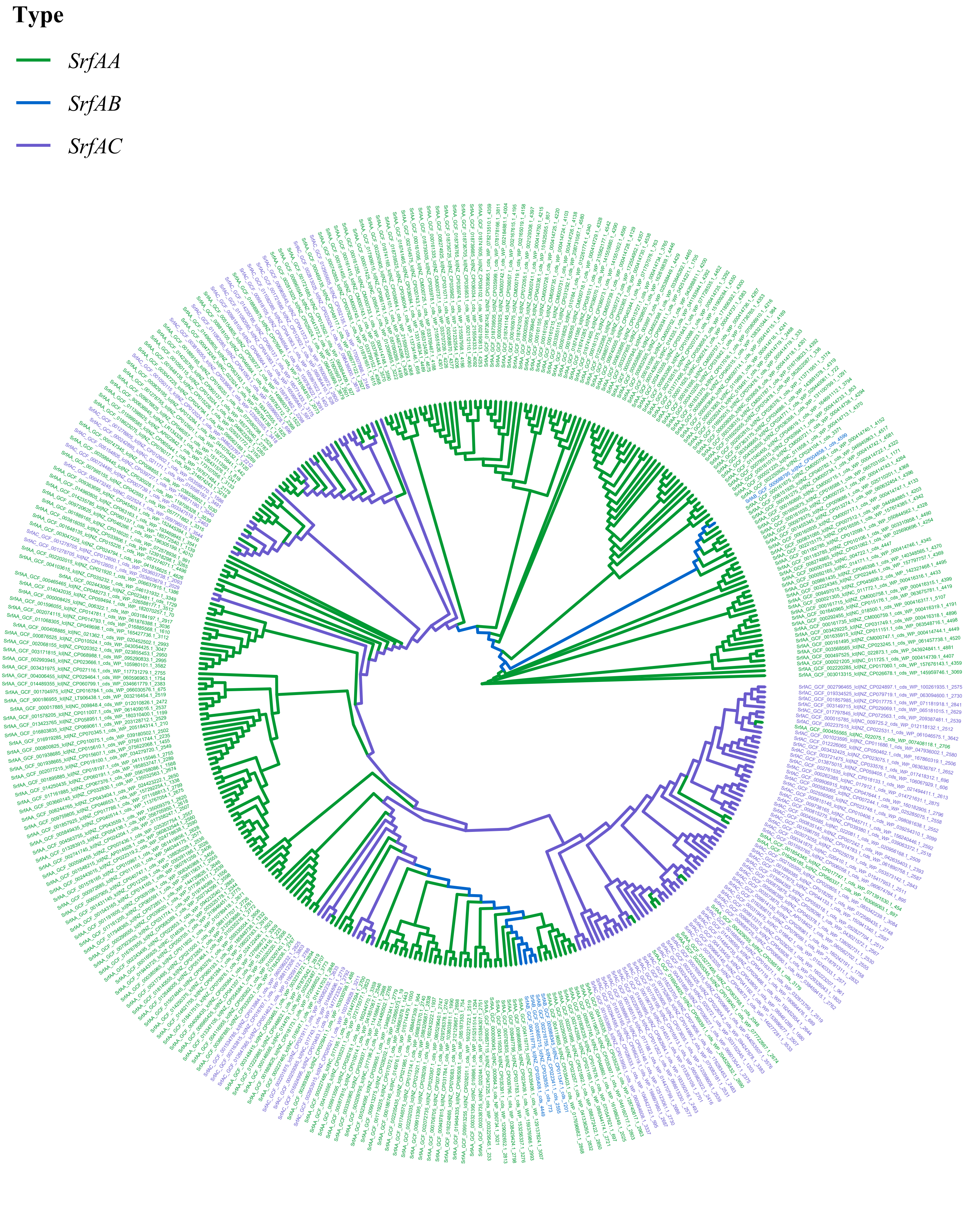

Figure S9. Detailed phylogenetic tree of *SrfAA*, *SrfAB* and *SrfAC* genes in OG0000898.

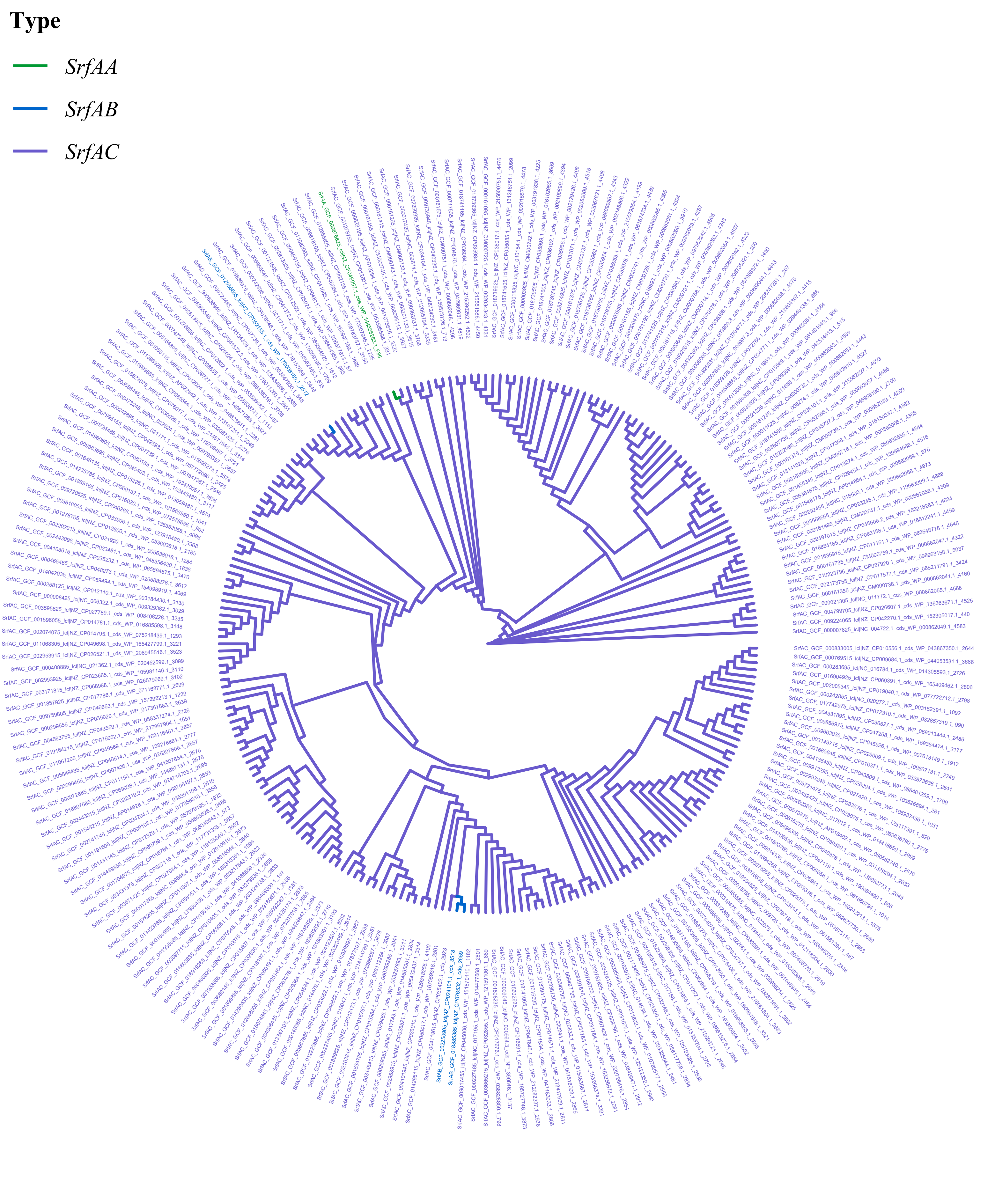

Figure S10. Detailed phylogenetic tree of *SrfAA*, *SrfAB* and *SrfAC* genes in OG0001033.

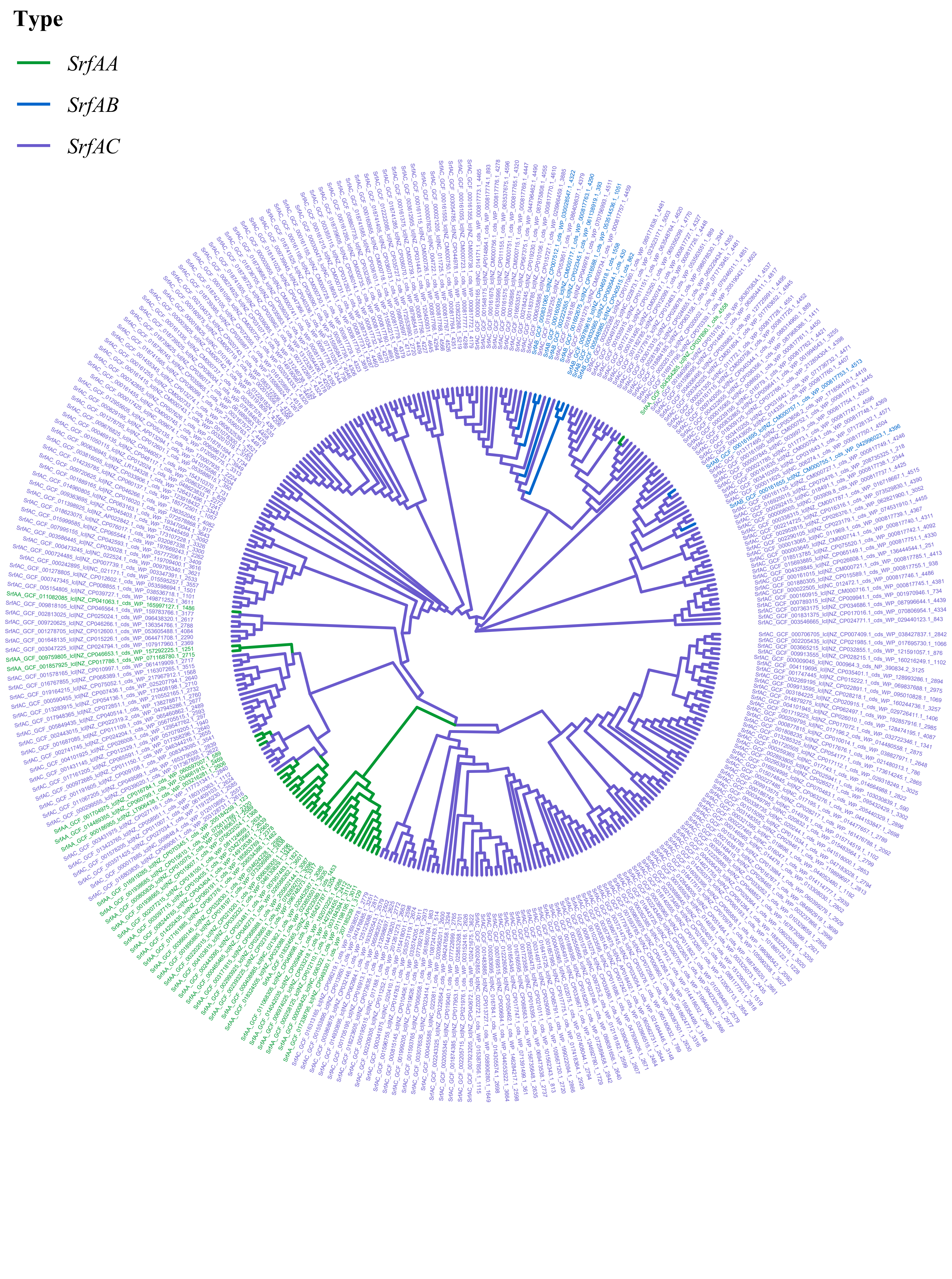

Figure S11. Detailed phylogenetic tree of *SrfAA*, *SrfAB* and *SrfAC* genes in OG0001616.

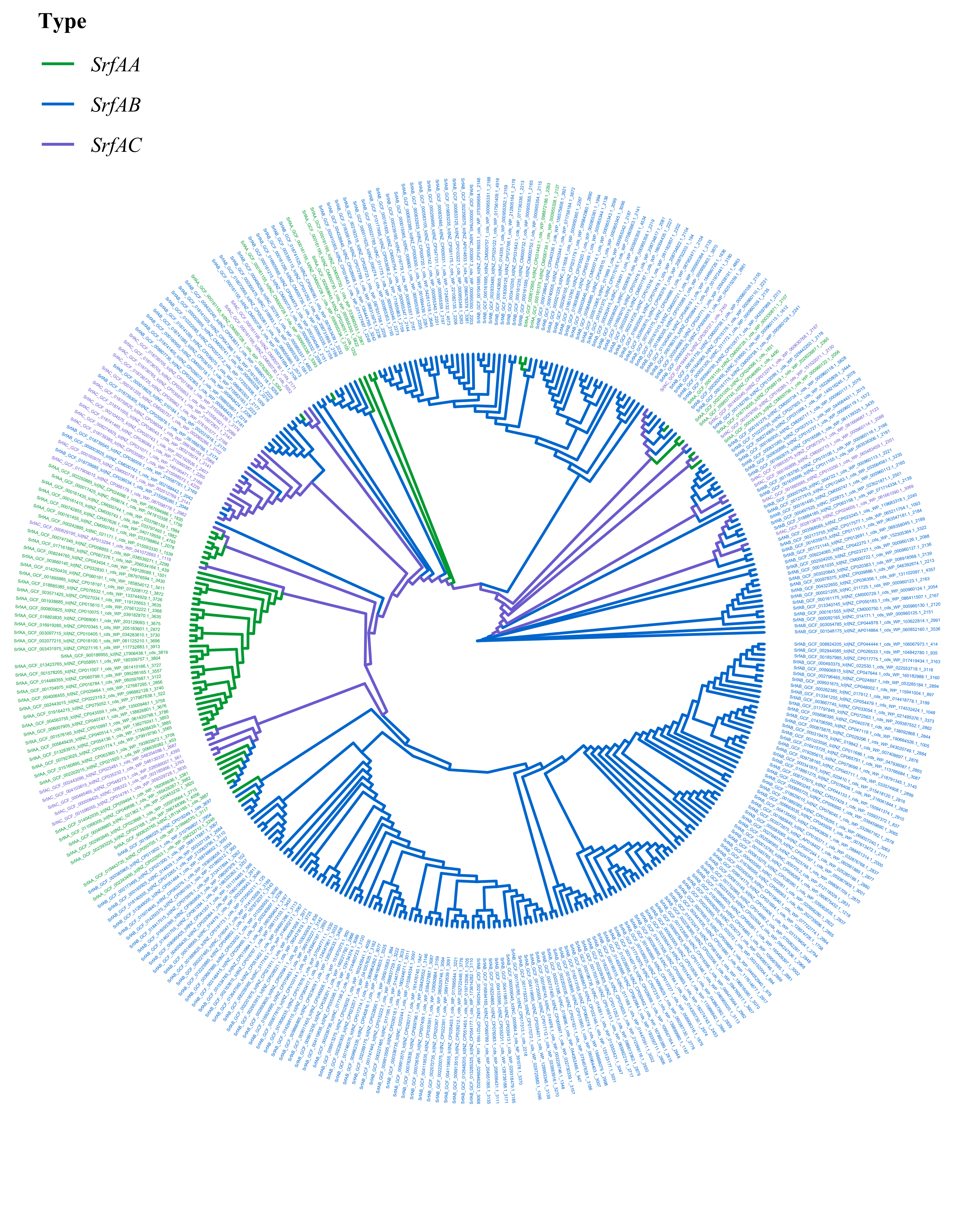

Figure S12. Detailed phylogenetic tree of *SrfAA*, *SrfAB* and *SrfAC* genes in OG0002144.

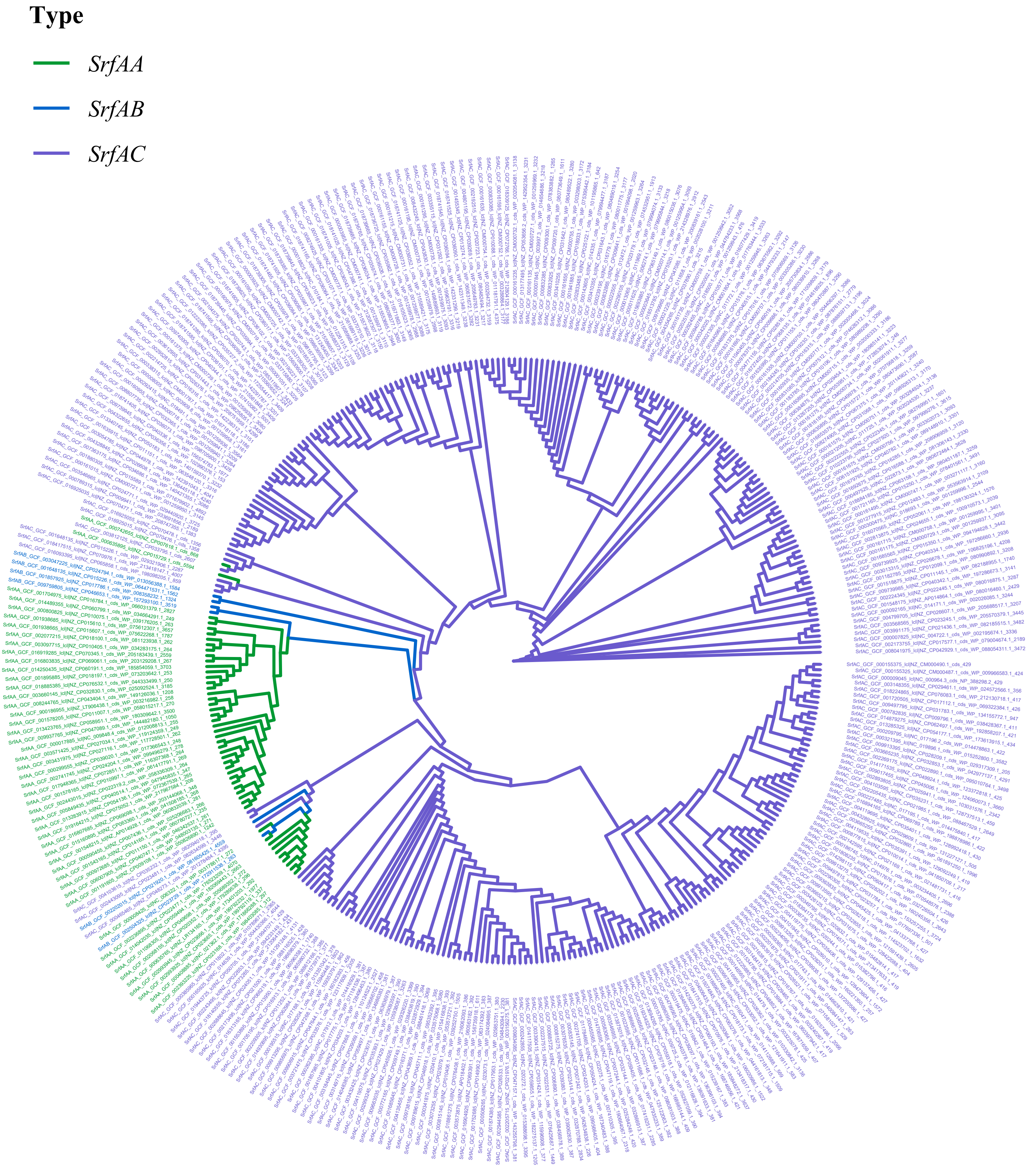

Figure S13. Detailed phylogenetic tree of *SrfAA*, *SrfAB* and *SrfAC* genes in OG0002176.

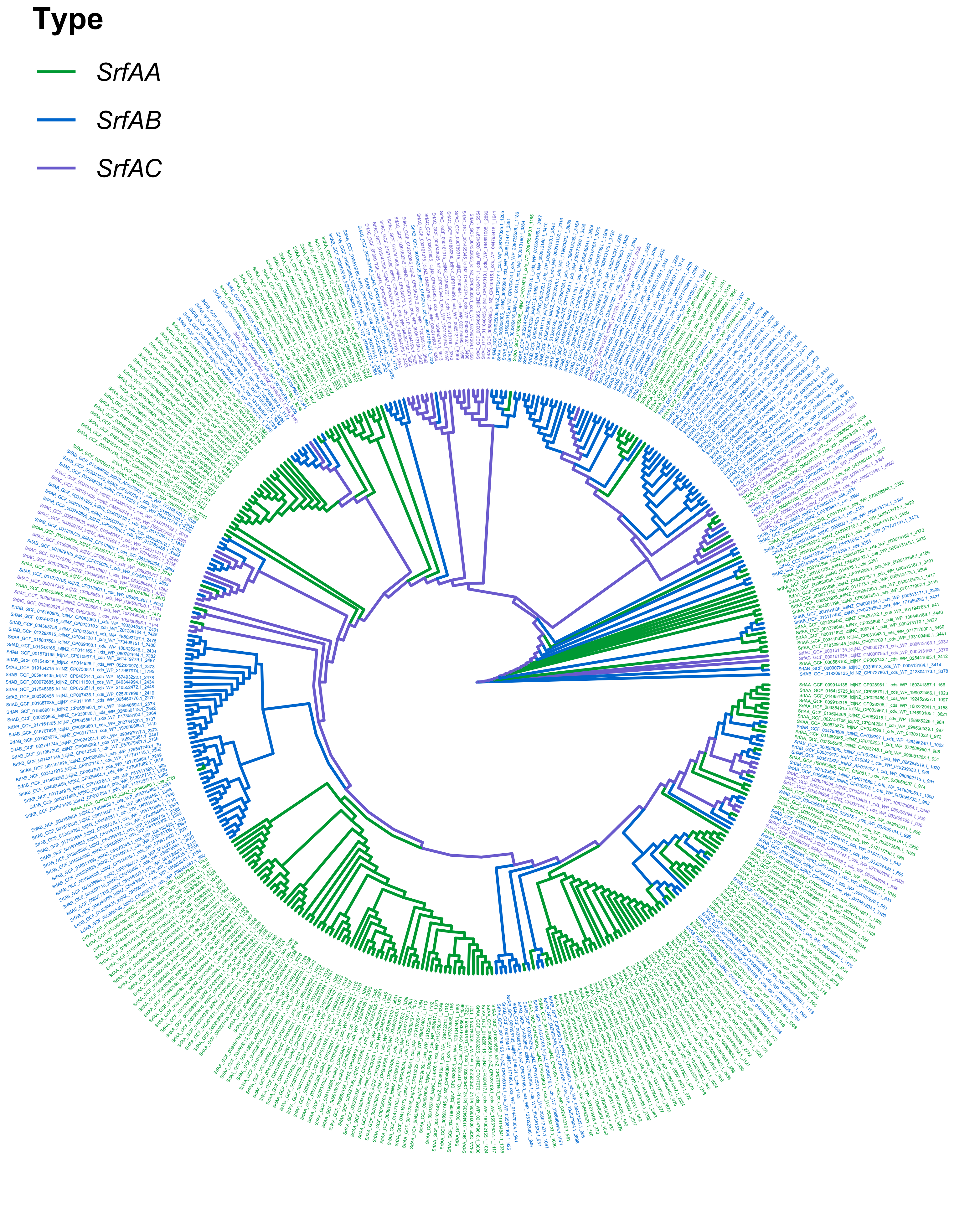

Figure S14. Detailed phylogenetic tree of *SrfAA*, *SrfAB* and *SrfAC* genes in OG0002286.

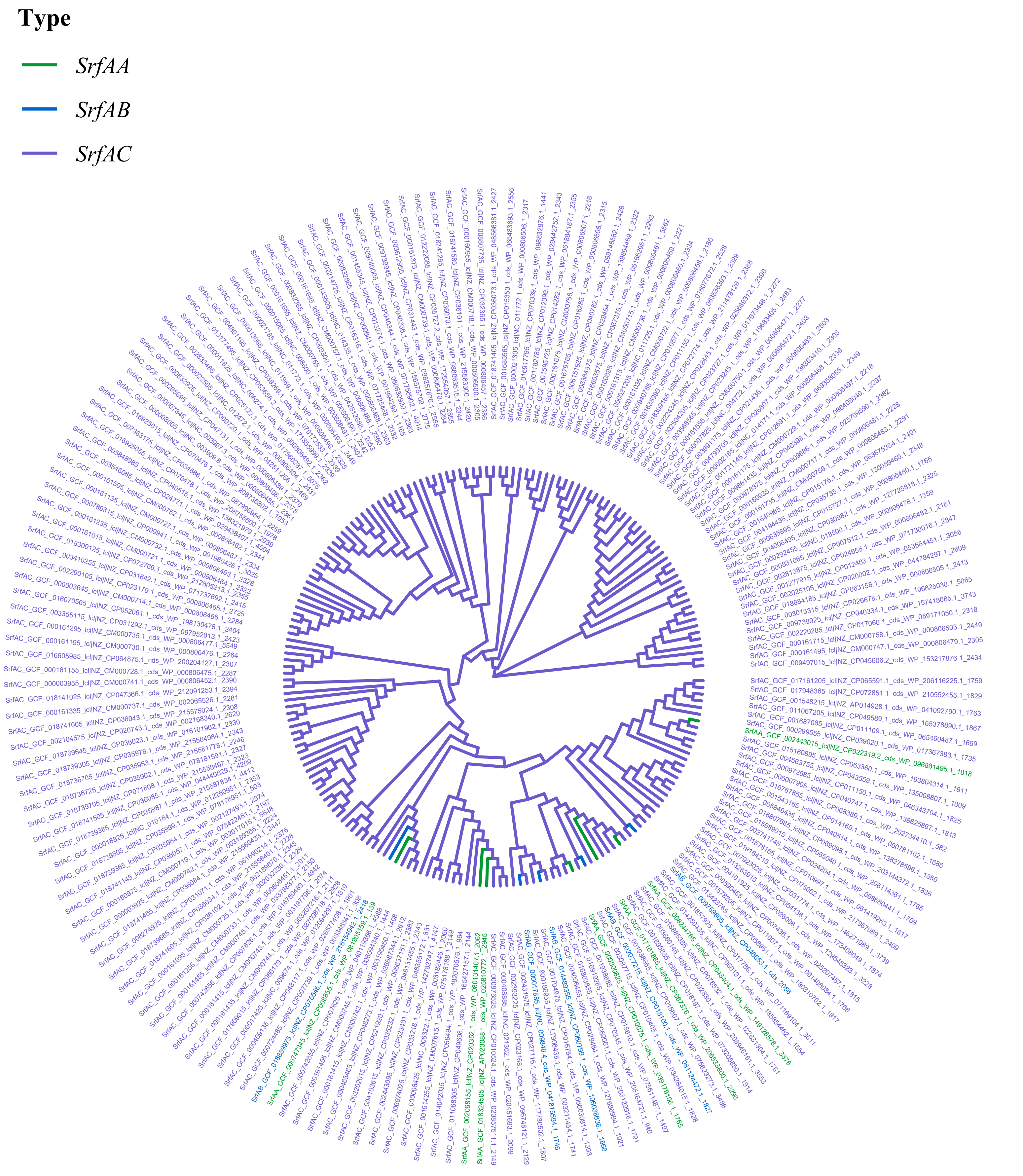

Figure S15. Detailed phylogenetic tree of *SrfAA*, *SrfAB* and *SrfAC* genes in OG0003280.

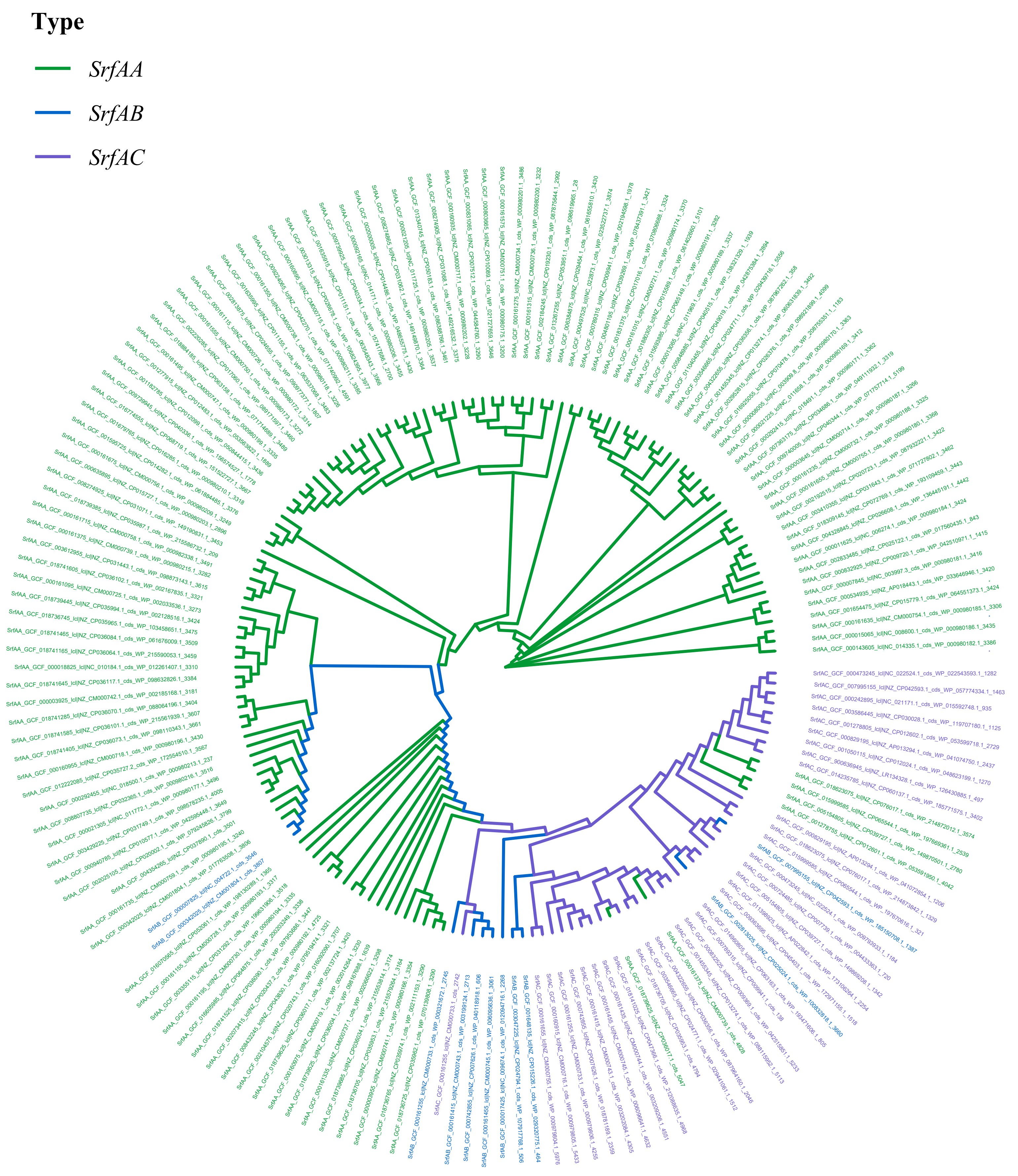

Figure S16. Detailed phylogenetic tree of *SrfAA*, *SrfAB* and *SrfAC* genes in OG0003557.

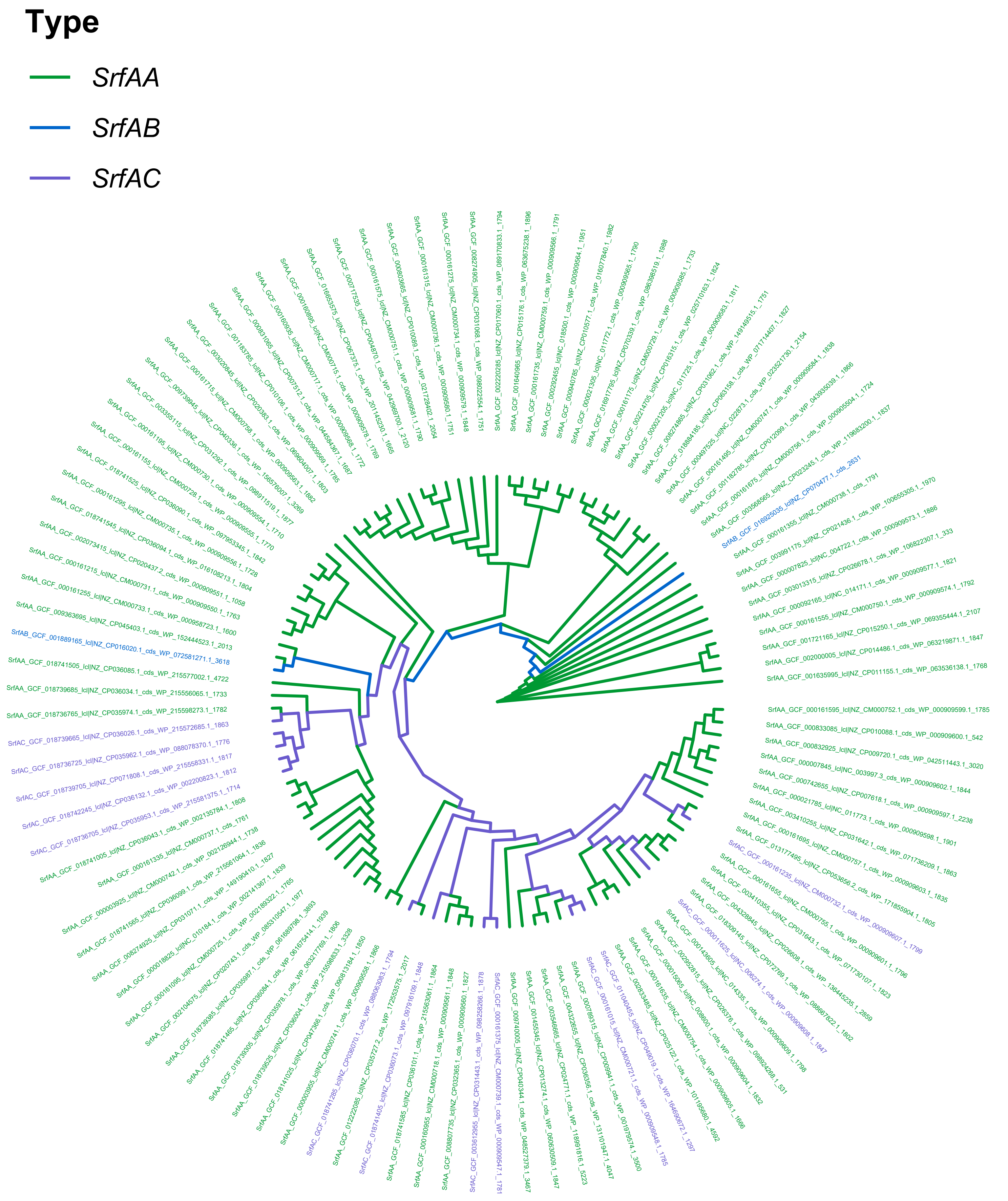

Figure S17. Detailed phylogenetic tree of *SrfAA*, *SrfAB* and *SrfAC* genes in OG0004686.

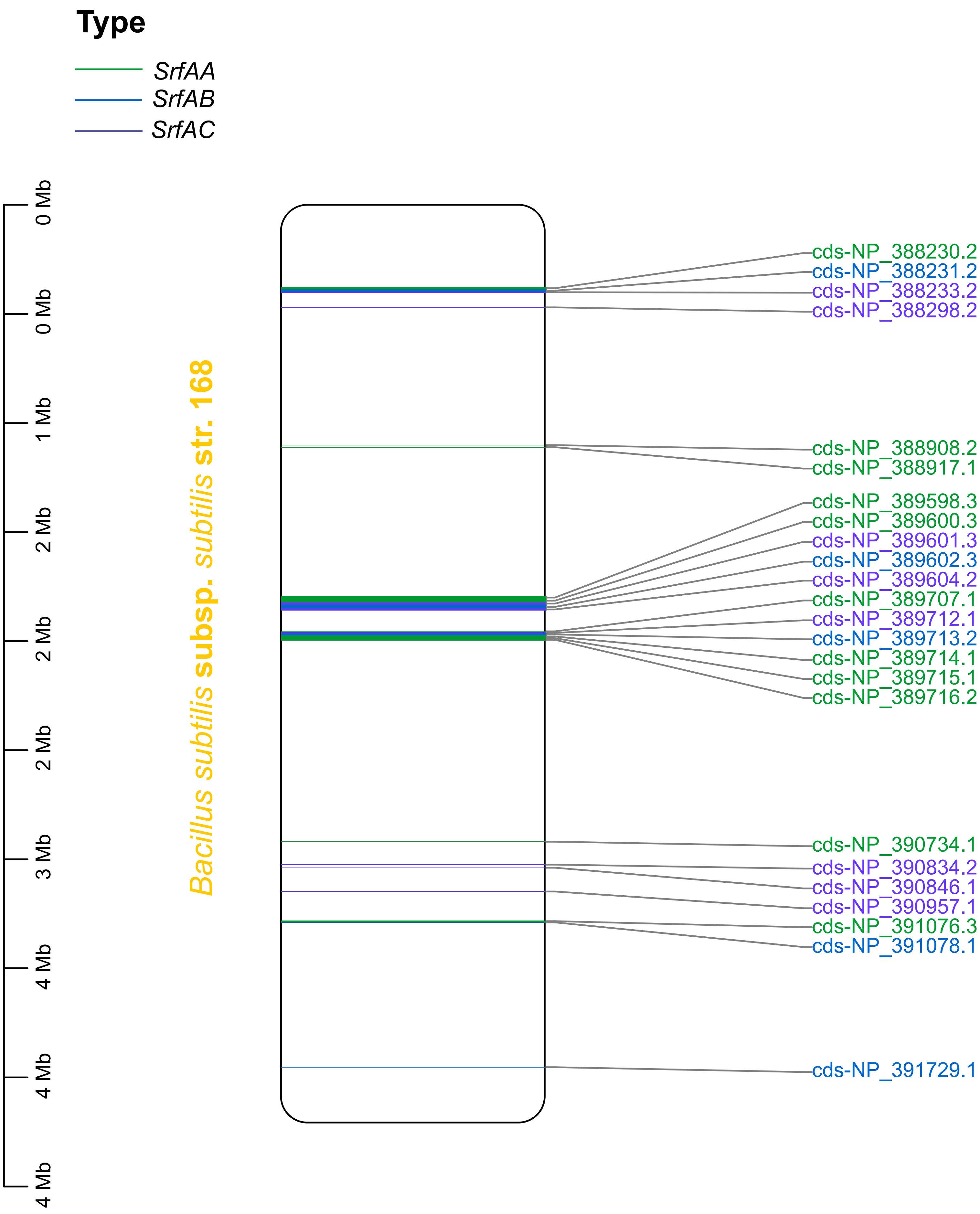

Figure S18. Distribution of *SrfAA*, *SrfAB* and *SrfAC* genes in the *Bacillus subtilis* subsp. *subtilis* str. 168 (GCF_000009045) genome.

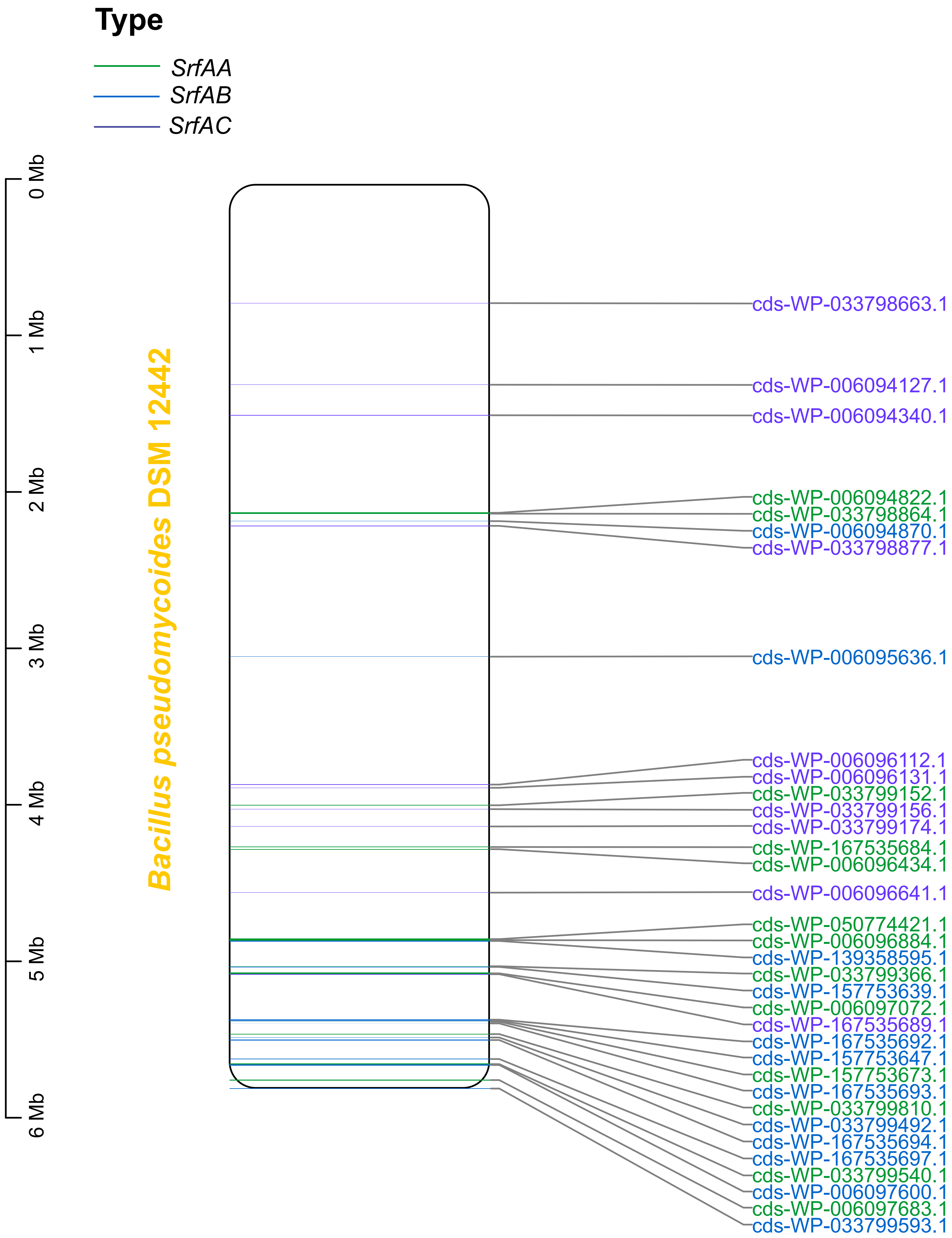

Figure S19. Distribution of *SrfAA*, *SrfAB* and *SrfAC* genes in the *Bacillus pseudomycoides* DSM 12442 (GCF_000161455) genome.

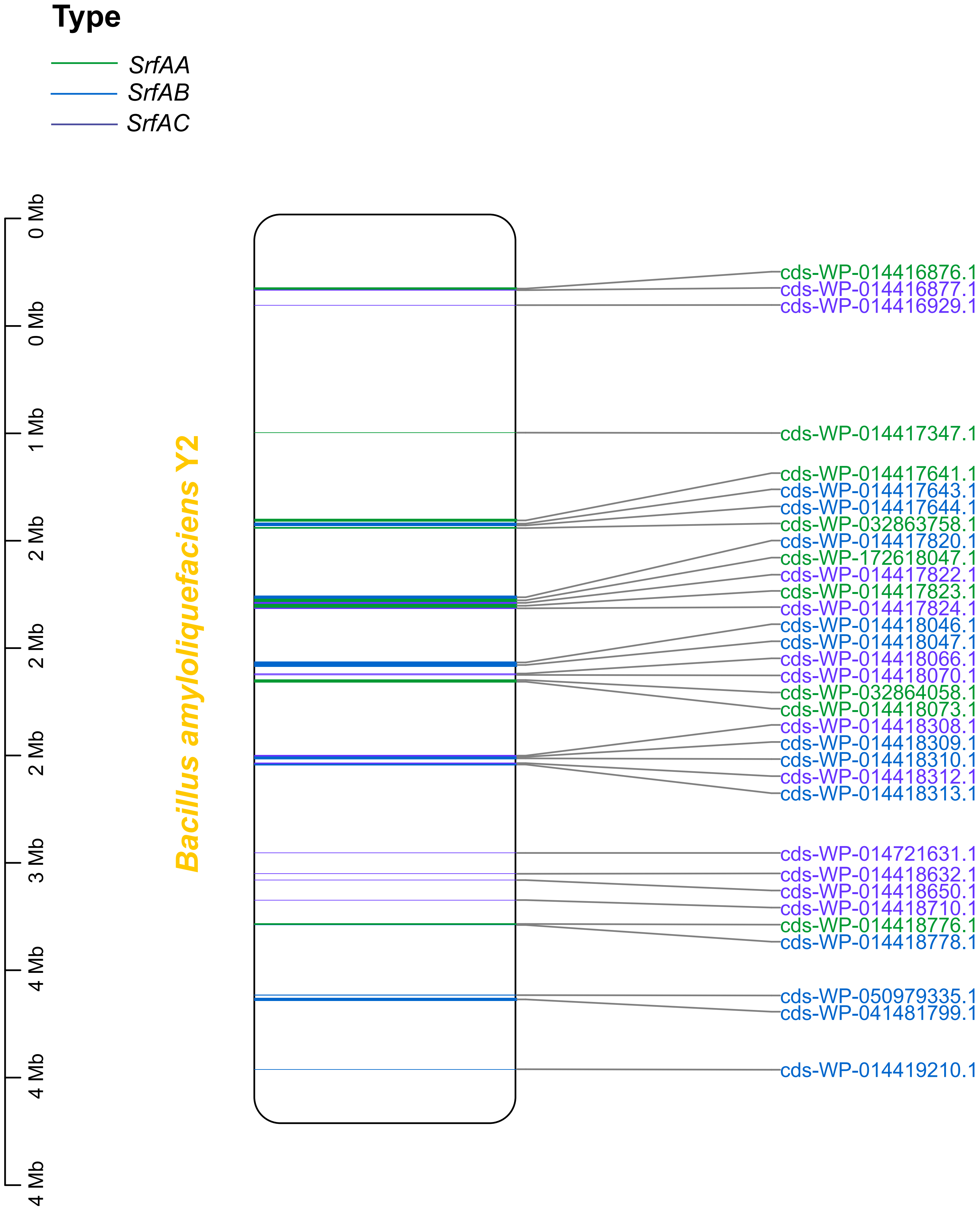

Figure S20. Distribution of *SrfAA*, *SrfAB* and *SrfAC* genes in the *Bacillus amyloliquefaciens* Y2 (GCF_000262385) genome.

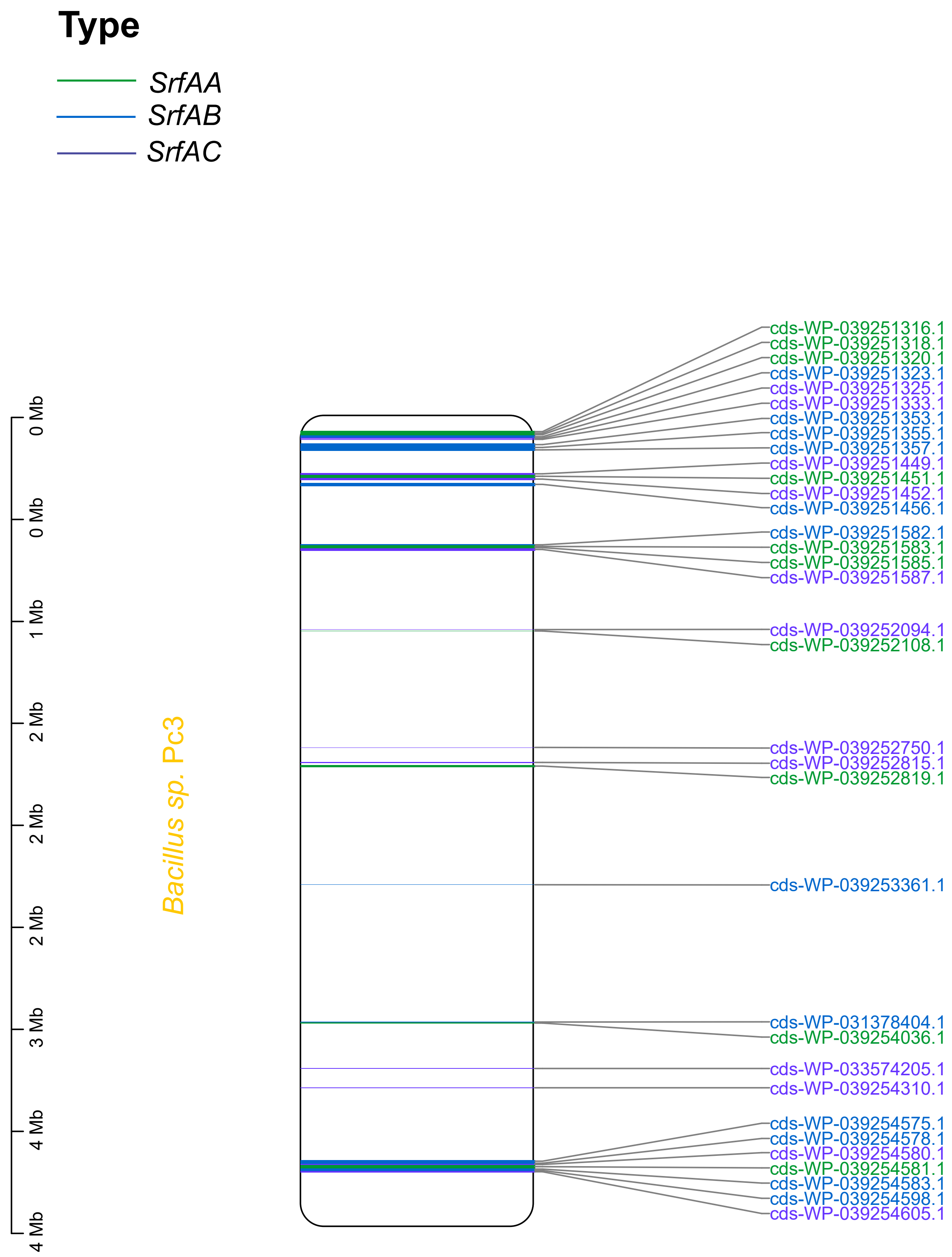

Figure S21. Distribution of *SrfAA*, *SrfAB* and *SrfAC* genes in the *Bacillus sp.* Pc3 (GCF_000815145) genome.

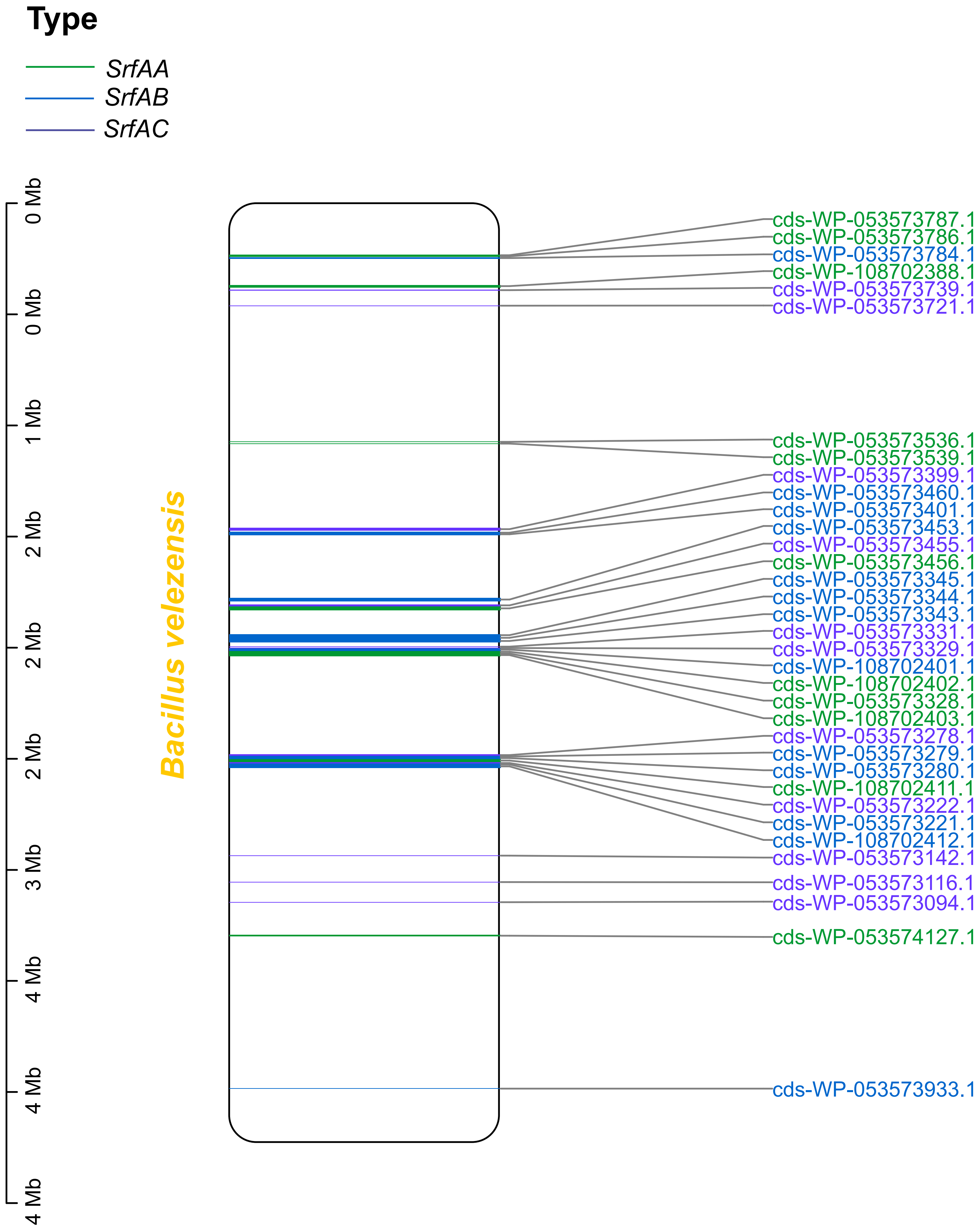

Figure S22. Distribution of *SrfAA*, *SrfAB* and *SrfAC* genes in the *Bacillus velezensis* (GCF_003073255) genome.

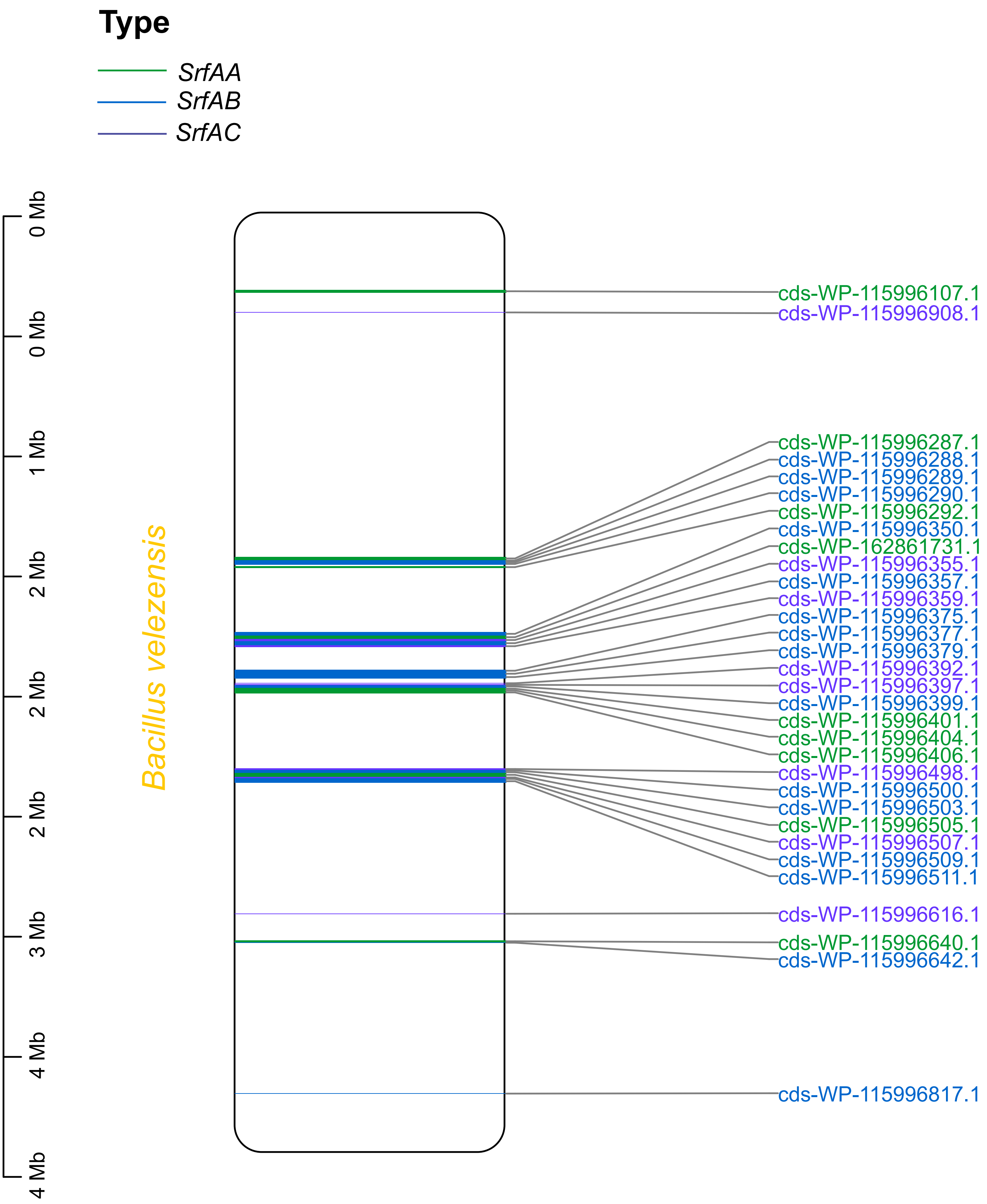

Figure S23. Distribution of *SrfAA*, *SrfAB* and *SrfAC* genes in the *Bacillus velezensis* (GCF_003390415) genome.

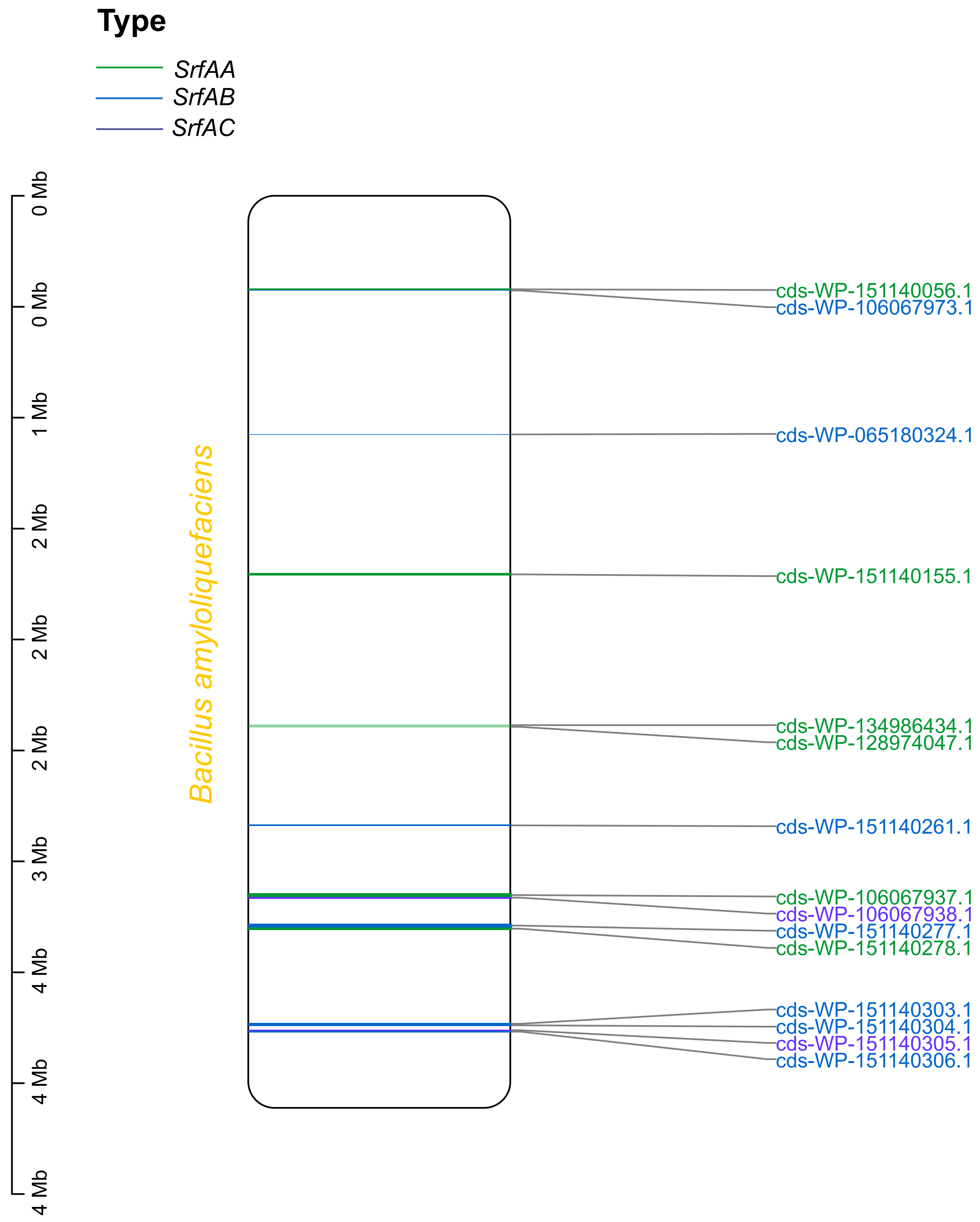

Figure S24. Distribution of *SrfAA*, *SrfAB* and *SrfAC* genes in the *Bacillus amyloliquefaciens* (GCF_008824205) genome.

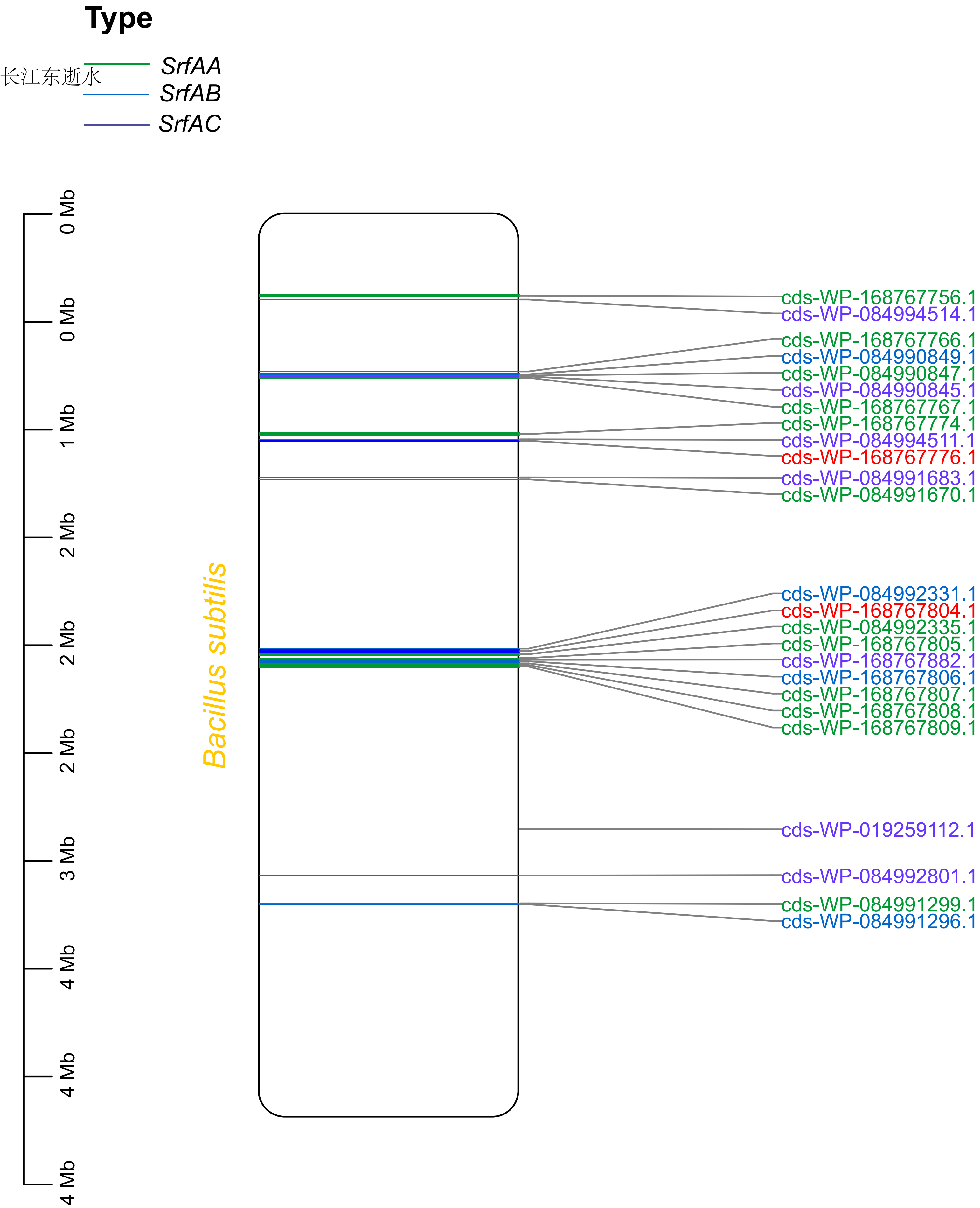

Figure S25. Distribution of *SrfAA*, *SrfAB* and *SrfAC* genes in the *Bacillus subtilis* (GCF_012647565) genome.

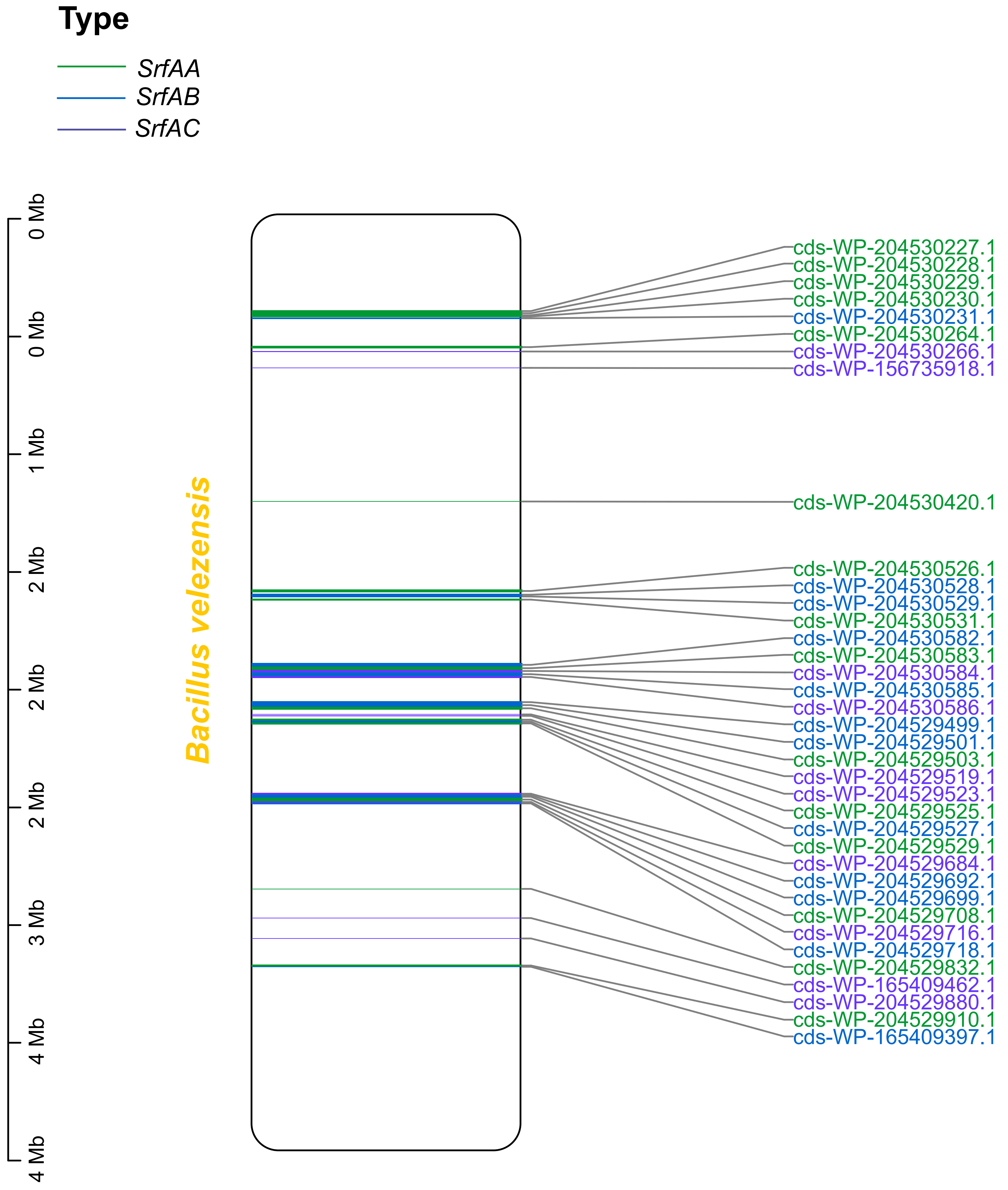

Figure 26. Distribution of *SrfAA*, *SrfAB* and *SrfAC* genes in the *Bacillus velezensis* (GCF_016904925) genome.
