# Supplementary material for: Genome-Wide Identification and Evolutionary Analyses of SrfA Operon Genes in Bacillus
Source: Genes (Basel). 2023 Feb 7;14(2):422. doi: 10.3390/genes14020422 (PMC9956979; doi:10.3390/genes14020422)
Supplement: Supplementary file 1 [file genes-14-00422-s001.zip › Supplementary Table S1,2.pdf]

Supporting information

Table S1. A list of 999 Bacillus species genomes used in this study.

Table S2. Number of *SrfAA*, *SrfAB* and *SrfAC* genes in 999 Bacillus species genomes.

Table S1 A list of 999 genomes of Bacillus species genomes used in this study.

Accession Number	Species
GCF_004101925	*Bacillus aerophilus*
GCF_009740005	*Bacillus albus*
GCF_000972685	*Bacillus altitudinis*
GCF_002443015	*Bacillus altitudinis*
GCF_002741745	*Bacillus altitudinis*
GCF_007923025	*Bacillus altitudinis*
GCF_015160895	*Bacillus altitudinis*
GCF_001191605	*Bacillus altitudinis*
GCF_001431145	*Bacillus altitudinis*
GCF_001431785	*Bacillus altitudinis*
GCF_001543165	*Bacillus altitudinis*
GCF_016767855	*Bacillus altitudinis*
GCF_016807685	*Bacillus altitudinis*
GCF_001700735	*Bacillus altitudinis*
GCF_000299555	*Bacillus altitudinis*
GCF_001908475	*Bacillus altitudinis*
GCF_011067205	*Bacillus altitudinis*
GCF_013283915	*Bacillus altitudinis*
GCF_013307105	*Bacillus altitudinis*
GCF_004563755	*Bacillus altitudinis*
GCF_005849435	*Bacillus altitudinis*
GCF_006007905	*Bacillus altitudinis*
GCF_017948365	*Bacillus altitudinis Strain: 6ww6*
GCF_015689015	*Bacillus altitudinis Strain: Ba1449*
GCF_017161205	*Bacillus altitudinis Strain: 1817*
GCF_019164215	*Bacillus altitudinis Strain: LZP 02*
GCF_019355135	*Bacillus altitudinis Strain: SCU11*
GCF_014622745	*Bacillus amyloliquefaciens*
GCF_014791945	*Bacillus amyloliquefaciens*
GCF_014792065	*Bacillus amyloliquefaciens*
GCF_014854735	*Bacillus amyloliquefaciens*
GCF_007362635	*Bacillus amyloliquefaciens*
GCF_014930895	*Bacillus amyloliquefaciens*
GCF_007827165	*Bacillus amyloliquefaciens*
GCF_007923205	*Bacillus amyloliquefaciens*
GCF_008534375	*Bacillus amyloliquefaciens*
GCF_015999565	*Bacillus amyloliquefaciens*
GCF_001483885	*Bacillus amyloliquefaciens*
GCF_008808015	*Bacillus amyloliquefaciens*
GCF_008824205	*Bacillus amyloliquefaciens*
GCF_001586105	*Bacillus amyloliquefaciens*

GCF_001596755	*Bacillus amyloliquefaciens*
GCF_003149695	*Bacillus amyloliquefaciens*
GCF_003149715	*Bacillus amyloliquefaciens*
GCF_003149795	*Bacillus amyloliquefaciens*
GCF_003312895	*Bacillus amyloliquefaciens*
GCF_001705195	*Bacillus amyloliquefaciens*
GCF_001874385	*Bacillus amyloliquefaciens*
GCF_001889285	*Bacillus amyloliquefaciens*
GCF_001922005	*Bacillus amyloliquefaciens*
GCF_003868675	*Bacillus amyloliquefaciens*
GCF_004006115	*Bacillus amyloliquefaciens*
GCF_013122255	*Bacillus amyloliquefaciens*
GCF_002173635	*Bacillus amyloliquefaciens*
GCF_013328835	*Bacillus amyloliquefaciens*
GCF_013341255	*Bacillus amyloliquefaciens*
GCF_002209305	*Bacillus amyloliquefaciens*
GCF_004421045	*Bacillus amyloliquefaciens*
GCF_019396925	*Bacillus amyloliquefaciens Strain: GKT04*
GCF_018223605	*Bacillus amyloliquefaciens Strain: Ba13*
GCF_017357905	*Bacillus amyloliquefaciens Strain: GXU-1*
GCF_018363035	*Bacillus amyloliquefaciens Strain: L-17*
GCF_018502525	*Bacillus amyloliquefaciens Strain: SN16-1*
GCF_000494835	*Bacillus amyloliquefaciens CC178*
GCF_000196735	*Bacillus amyloliquefaciens DSM 7 = ATCC 23350*
GCF_000242855	*Bacillus amyloliquefaciens IT-45*
GCF_000835145	*Bacillus amyloliquefaciens KHG19*
GCF_000508265	*Bacillus amyloliquefaciens LFB112*
GCF_000204275	*Bacillus amyloliquefaciens LL3*
GCF_017815555	*Bacillus amyloliquefaciens Strain: G10*
GCF_017603845	*Bacillus amyloliquefaciens Strain: XJ5*
GCF_000195515	*Bacillus amyloliquefaciens TA208*
GCF_001593785	*Bacillus amyloliquefaciens UMAF6614*
GCF_001593765	*Bacillus amyloliquefaciens UMAF6639*
GCF_000221645	*Bacillus amyloliquefaciens XH7*
GCF_000262385	*Bacillus amyloliquefaciens Y2*
GCF_006088855	*Bacillus anthracis*
GCF_014249775	*Bacillus anthracis*
GCF_000875715	*Bacillus anthracis*
GCF_002277915	*Bacillus anthracis*
GCF_002356575	*Bacillus anthracis*
GCF_006742565	*Bacillus anthracis*
GCF_001277955	*Bacillus anthracis*
GCF_001543225	*Bacillus anthracis*
GCF_002980615	*Bacillus anthracis*

GCF_001654475	*Bacillus anthracis*
GCF_001683065	*Bacillus anthracis*
GCF_001683095	*Bacillus anthracis*
GCF_001683135	*Bacillus anthracis*
GCF_003227955	*Bacillus anthracis*
GCF_001683155	*Bacillus anthracis*
GCF_001683175	*Bacillus anthracis*
GCF_001683195	*Bacillus anthracis*
GCF_001683215	*Bacillus anthracis*
GCF_001683235	*Bacillus anthracis*
GCF_001683255	*Bacillus anthracis*
GCF_001683275	*Bacillus anthracis*
GCF_001683295	*Bacillus anthracis*
GCF_003410255	*Bacillus anthracis*
GCF_003410355	*Bacillus anthracis*
GCF_009806595	*Bacillus anthracis*
GCF_009806615	*Bacillus anthracis*
GCF_009806635	*Bacillus anthracis*
GCF_009806655	*Bacillus anthracis*
GCF_009806675	*Bacillus anthracis*
GCF_009806695	*Bacillus anthracis*
GCF_001936375	*Bacillus anthracis*
GCF_000534935	*Bacillus anthracis*
GCF_001990245	*Bacillus anthracis*
GCF_012273315	*Bacillus anthracis*
GCF_000725325	*Bacillus anthracis*
GCF_012273335	*Bacillus anthracis*
GCF_012273355	*Bacillus anthracis*
GCF_000742315	*Bacillus anthracis*
GCF_012273375	*Bacillus anthracis*
GCF_000742655	*Bacillus anthracis*
GCF_000742695	*Bacillus anthracis*
GCF_000742875	*Bacillus anthracis*
GCF_000831505	*Bacillus anthracis*
GCF_000832425	*Bacillus anthracis*
GCF_000832445	*Bacillus anthracis*
GCF_000832465	*Bacillus anthracis*
GCF_004328845	*Bacillus anthracis*
GCF_000832505	*Bacillus anthracis*
GCF_002208785	*Bacillus anthracis*
GCF_000832565	*Bacillus anthracis*
GCF_000832585	*Bacillus anthracis*
GCF_013349165	*Bacillus anthracis*
GCF_000832665	*Bacillus anthracis*

GCF_013349185	*Bacillus anthracis*
GCF_000832725	*Bacillus anthracis*
GCF_013349245	*Bacillus anthracis*
GCF_000832745	*Bacillus anthracis*
GCF_000832965	*Bacillus anthracis*
GCF_000833065	*Bacillus anthracis*
GCF_000833125	*Bacillus anthracis*
GCF_000559005	*Bacillus anthracis 52-G*
GCF_000558965	*Bacillus anthracis 8903-G*
GCF_000558985	*Bacillus anthracis 9080-G*
GCF_000022865	*Bacillus anthracis str. A0248*
GCF_000512835	*Bacillus anthracis str. A16*
GCF_000512775	*Bacillus anthracis str. A16R*
GCF_000007845	*Bacillus anthracis str. Ames*
GCF_000008445	*Bacillus anthracis str. 'Ames Ancestor'*
GCF_000295695	*Bacillus anthracis str. BF1*
GCF_000021445	*Bacillus anthracis str. CDC 684*
GCF_000258885	*Bacillus anthracis str. H9401*
GCF_000008165	*Bacillus anthracis str. Sterne*
GCF_000832635	*Bacillus anthracis str. Sterne*
GCF_000583105	*Bacillus anthracis str. SVA11*
GCF_000833275	*Bacillus anthracis str. Turkey32*
GCF_000832785	*Bacillus anthracis str. V770-NP-1R*
GCF_000742895	*Bacillus anthracis str. Vollum*
GCF_019693235	*Bacillus anthracis Strain: P04210076*
GCF_002688645	*Bacillus atrophaeus*
GCF_002173495	*Bacillus atrophaeus*
GCF_002243495	*Bacillus atrophaeus*
GCF_018140555	*Bacillus atrophaeus Strain: CNY01*
GCF_019443725	*Bacillus atrophaeusStrain: PENSV20*
GCF_000165925	*Bacillus atrophaeus 1942*
GCF_000742675	*Bacillus atrophaeus subsp. globigii*
GCF_000385965	*Bacillus atrophaeus UCMB-5137*
GCF_001721685	*Bacillus beveridgei*
GCF_000831065	*Bacillus bombysepticus str. Wang*
GCF_000835185	*Bacillus cereus*
GCF_006384875	*Bacillus cereus*
GCF_002290105	*Bacillus cereus*
GCF_000978375	*Bacillus cereus*
GCF_008041975	*Bacillus cereus*
GCF_001277915	*Bacillus cereus*
GCF_002813875	*Bacillus cereus*
GCF_001518875	*Bacillus cereus*
GCF_016027015	*Bacillus cereus*

GCF_003013315	*Bacillus cereus*
GCF_003020845	*Bacillus cereus*
GCF_009497015	*Bacillus cereus*
GCF_016727405	*Bacillus cereus*
GCF_016774535	*Bacillus cereus*
GCF_016774555	*Bacillus cereus*
GCF_016774575	*Bacillus cereus*
GCF_001635915	*Bacillus cereus*
GCF_001635955	*Bacillus cereus*
GCF_001635995	*Bacillus cereus*
GCF_016917795	*Bacillus cereus*
GCF_009739925	*Bacillus cereus*
GCF_009739965	*Bacillus cereus*
GCF_009739985	*Bacillus cereus*
GCF_001721145	*Bacillus cereus*
GCF_003568565	*Bacillus cereus*
GCF_001880305	*Bacillus cereus*
GCF_010223795	*Bacillus cereus*
GCF_010232525	*Bacillus cereus*
GCF_001941885	*Bacillus cereus*
GCF_001941905	*Bacillus cereus*
GCF_001941925	*Bacillus cereus*
GCF_000635895	*Bacillus cereus*
GCF_004006495	*Bacillus cereus*
GCF_002000005	*Bacillus cereus*
GCF_013112375	*Bacillus cereus*
GCF_002117465	*Bacillus cereus*
GCF_013177495	*Bacillus cereus*
GCF_000789315	*Bacillus cereus*
GCF_013267235	*Bacillus cereus*
GCF_013267255	*Bacillus cereus*
GCF_013267275	*Bacillus cereus*
GCF_013267455	*Bacillus cereus*
GCF_013267475	*Bacillus cereus*
GCF_013267775	*Bacillus cereus*
GCF_000832525	*Bacillus cereus*
GCF_002214705	*Bacillus cereus*
GCF_002214725	*Bacillus cereus*
GCF_002214765	*Bacillus cereus*
GCF_002215175	*Bacillus cereus*
GCF_002216125	*Bacillus cereus*
GCF_004771155	*Bacillus cereus*
GCF_000832765	*Bacillus cereus*
GCF_002220285	*Bacillus cereus*

GCF_004801195	*Bacillus cereus*
GCF_013394245	*Bacillus cereus*
GCF_005707595	*Bacillus cereus*
GCF_018309125	*Bacillus cereus Strain: BC06*
GCF_018309165	*Bacillus cereus Strain: BC33*
GCF_018884185	*Bacillus cereus Strain: CF4-51*
GCF_018309145	*Bacillus cereus Strain: BC07*
GCF_016117775	*Bacillus cereus Strain: FDAARGOS_919*
GCF_002952815	*Bacillus cereus Strain: G9241*
GCF_000022505	*Bacillus cereus 03BB102*
GCF_000832405	*Bacillus cereus 03BB102*
GCF_000832865	*Bacillus cereus 03BB108*
GCF_000160935	*Bacillus cereus 172560W*
GCF_000161135	*Bacillus cereus 95/8201*
GCF_000161375	*Bacillus cereus AH1271*
GCF_000161395	*Bacillus cereus AH1272*
GCF_000003955	*Bacillus cereus AH1273*
GCF_000021225	*Bacillus cereus AH187*
GCF_000161355	*Bacillus cereus AH676*
GCF_000021785	*Bacillus cereus AH820*
GCF_000160895	*Bacillus cereus ATCC 10876*
GCF_000008005	*Bacillus cereus ATCC 10987*
GCF_006094295	*Bacillus cereus ATCC 14579*
GCF_000007825	*Bacillus cereus ATCC 14579*
GCF_002504205	*Bacillus cereus ATCC 14579*
GCF_000161015	*Bacillus cereus ATCC 4342*
GCF_000832845	*Bacillus cereus ATCC 4342*
GCF_000021205	*Bacillus cereus B4264*
GCF_000161115	*Bacillus cereus BDRD-Cer4*
GCF_000161095	*Bacillus cereus BDRD-ST196*
GCF_000161055	*Bacillus cereus BDRD-ST24*
GCF_000160915	*Bacillus cereus BGSC 6E1*
GCF_000143605	*Bacillus cereus biovar anthracis str. CI*
GCF_002224345	*Bacillus cereus C1L*
GCF_000832385	*Bacillus cereus D17*
GCF_000011625	*Bacillus cereus E33L*
GCF_000833045	*Bacillus cereus E33L*
GCF_000338315	*Bacillus cereus F*
GCF_000161315	*Bacillus cereus F65185*
GCF_000239195	*Bacillus cereus F837/76*
GCF_000292415	*Bacillus cereus FRI-35*
GCF_000832805	*Bacillus cereus G9241*
GCF_000021305	*Bacillus cereus G9842*
GCF_000003645	*Bacillus cereus m1293*

GCF_000161035	*Bacillus cereus m1550*
GCF_000283675	*Bacillus cereus NC7401*
GCF_000013065	*Bacillus cereus Q1*
GCF_000161175	*Bacillus cereus Rock1-15*
GCF_000161155	*Bacillus cereus Rock1-3*
GCF_000161195	*Bacillus cereus Rock3-28*
GCF_000161215	*Bacillus cereus Rock3-29*
GCF_000161235	*Bacillus cereus Rock3-42*
GCF_000161255	*Bacillus cereus Rock3-44*
GCF_000161295	*Bacillus cereus Rock4-18*
GCF_000161275	*Bacillus cereus Rock4-2*
GCF_007923085	*Bacillus cereus Strain: Co1-1*
GCF_002251005	*Bacillus cytotoxicus*
GCF_002251025	*Bacillus cytotoxicus*
GCF_002251045	*Bacillus cytotoxicus*
GCF_002251055	*Bacillus cytotoxicus*
GCF_002251115	*Bacillus cytotoxicus*
GCF_002250885	*Bacillus cytotoxicus*
GCF_002250905	*Bacillus cytotoxicus*
GCF_002250925	*Bacillus cytotoxicus*
GCF_002250945	*Bacillus cytotoxicus*
GCF_002250965	*Bacillus cytotoxicus*
GCF_017909295	*Bacillus cytotoxicus Strain: E28.3*
GCF_017909135	*Bacillus cytotoxicus Strain: SM1.1*
GCF_017909395	*Bacillus cytotoxicus Strain: E17.4*
GCF_017909435	*Bacillus cytotoxicus Strain: E8.1*
GCF_017909235	*Bacillus cytotoxicus Strain: PDT2.12*
GCF_017909015	*Bacillus cytotoxicus Strain: SM2.8*
GCF_000017425	*Bacillus cytotoxicus NVH 391-98*
GCF_007995155	*Bacillus dafuensis*
GCF_900636945	*Bacillus freudenreichii*
GCF_002443095	*Bacillus glycinifermentans*
GCF_004103615	*Bacillus glycinifermentans*
GCF_900093775	*Bacillus glycinifermentans*
GCF_001278705	*Bacillus gobiensis*
GCF_018623075	*Bacillus haikouensis Strain: MNJ12*
GCF_014621755	*Bacillus halotolerans*
GCF_006965425	*Bacillus halotolerans*
GCF_016093395	*Bacillus halotolerans*
GCF_004006435	*Bacillus halotolerans*
GCF_018417515	*Bacillus halotolerans Strain: MBH1*
GCF_014042035	*Bacillus haynesii*
GCF_001534785	*Bacillus inaquosorum*
GCF_003148415	*Bacillus inaquosorum*

GCF_019598985	*Bacillus inaquosorum Strain: CCSR02*
GCF_000473245	*Bacillus infantis NRRL B-14911*
GCF_000959025	*Bacillus intestinalis*
GCF_006094335	*Bacillus licheniformis*
GCF_006494795	*Bacillus licheniformis*
GCF_014673515	*Bacillus licheniformis*
GCF_006974025	*Bacillus licheniformis*
GCF_002504325	*Bacillus licheniformis*
GCF_007855455	*Bacillus licheniformis*
GCF_016026835	*Bacillus licheniformis*
GCF_002966955	*Bacillus licheniformis*
GCF_016126875	*Bacillus licheniformis*
GCF_001596055	*Bacillus licheniformis*
GCF_009662395	*Bacillus licheniformis*
GCF_003253815	*Bacillus licheniformis*
GCF_003339545	*Bacillus licheniformis*
GCF_001726125	*Bacillus licheniformis*
GCF_003595625	*Bacillus licheniformis*
GCF_003606405	*Bacillus licheniformis*
GCF_001914255	*Bacillus licheniformis*
GCF_003952865	*Bacillus licheniformis*
GCF_004101525	*Bacillus licheniformis*
GCF_002074075	*Bacillus licheniformis*
GCF_002074095	*Bacillus licheniformis*
GCF_002074115	*Bacillus licheniformis*
GCF_002074135	*Bacillus licheniformis*
GCF_004103575	*Bacillus licheniformis*
GCF_002173675	*Bacillus licheniformis*
GCF_004119755	*Bacillus licheniformis*
GCF_002174255	*Bacillus licheniformis*
GCF_004119795	*Bacillus licheniformis*
GCF_004526275	*Bacillus licheniformis*
GCF_002236895	*Bacillus licheniformis*
GCF_902386215	*Bacillus licheniformis*
GCF_017338795	*Bacillus licheniformis Strain: CP6*
GCF_015689075	*Bacillus licheniformis Strain: LCDD6*
GCF_002953475	*Bacillus licheniformis Strain: MBGJa67*
GCF_000008425	*Bacillus licheniformis DSM 13 = ATCC 14580*
GCF_000011645	*Bacillus licheniformis DSM 13 = ATCC 14580*
GCF_000258125	*Bacillus licheniformis WX-02*
GCF_009739945	*Bacillus luti*
GCF_000724485	*Bacillus methanolicus MGA3*
GCF_003612955	*Bacillus mobilis*
GCF_015074845	*Bacillus mojavensis*

GCF_012648005	*Bacillus mojavensis*
GCF_008274925	*Bacillus mycoides*
GCF_000160975	*Bacillus mycoides*
GCF_000161335	*Bacillus mycoides*
GCF_002104575	*Bacillus mycoides*
GCF_000775975	*Bacillus mycoides*
GCF_000832605	*Bacillus mycoides*
GCF_018736725	*Bacillus mycoides Strain: BPN07/3*
GCF_018736765	*Bacillus mycoides Strain: BPN102*
GCF_018739385	*Bacillus mycoides Strain: BPN29/1*
GCF_018742205	*Bacillus mycoides Strain: BPN29/1*
GCF_018739485	*Bacillus mycoides Strain: BPN36/3*
GCF_018739525	*Bacillus mycoides Strain: BPN37/2*
GCF_018739625	*Bacillus mycoides Strain: BPN51/1*
GCF_018739645	*Bacillus mycoides Strain: BPN52/2*
GCF_018741085	*Bacillus mycoides Strain: BPN601*
GCF_018741145	*Bacillus mycoides Strain: JAS06/1*
GCF_018741165	*Bacillus mycoides Strain: JAS06/3*
GCF_018742045	*Bacillus mycoides Strain: JAS12/5*
GCF_018741565	*Bacillus mycoides Strain: JAS23/1*
GCF_018741605	*Bacillus mycoides Strain: JAS391*
GCF_018741645	*Bacillus mycoides Strain: JAS481*
GCF_018736705	*Bacillus mycoides Strain: BPN03/1*
GCF_018736745	*Bacillus mycoides Strain: BPN09/1*
GCF_018739305	*Bacillus mycoides Strain: BPN121*
GCF_018739505	*Bacillus mycoides Strain: BPN37/1*
GCF_018739545	*Bacillus mycoides Strain: BPN43/2*
GCF_018739665	*Bacillus mycoides Strain: BPN54/2*
GCF_018740945	*Bacillus mycoides Strain: BPN573*
GCF_018741005	*Bacillus mycoides Strain: BPN58/4*
GCF_018739705	*Bacillus mycoides Strain: JAS014*
GCF_018742185	*Bacillus mycoides Strain: JAS06/1*
GCF_018741465	*Bacillus mycoides Strain: JAS1004*
GCF_018741505	*Bacillus mycoides Strain: JAS12/5*
GCF_018751995	*Bacillus mycoides Strain: JAS15/1*
GCF_018742225	*Bacillus mycoides Strain: JAS635*
GCF_018742105	*Bacillus mycoides Strain: JAS85/1*
GCF_018742265	*Bacillus mycoides Strain: JAS85/1*
GCF_018742285	*Bacillus mycoides Strain: JAS94/5*
GCF_000003925	*Bacillus mycoides DSM 2048*
GCF_000018825	*Bacillus mycoides KBAB4*
GCF_018739365	*Bacillus mycoides Strain: BPN211*
GCF_018739445	*Bacillus mycoides Strain: BPN36/2*
GCF_018739685	*Bacillus mycoides Strain: BPN57/2*

GCF_016117755	*Bacillus mycoides Strain: FDAARGOS_924*
GCF_018742245	*Bacillus mycoides Strain: JAS823*
GCF_018141025	*Bacillus nitratireducens Strain: BM02*
GCF_016925055	*Bacillus pacificus Strain: Colony7*
GCF_016925035	*Bacillus pacificus Strain: Colony 6*
GCF_016925015	*Bacillus pacificus Strain: Colony501*
GCF_000876525	*Bacillus paralicheniformis*
GCF_002393225	*Bacillus paralicheniformis*
GCF_008365255	*Bacillus paralicheniformis*
GCF_002993925	*Bacillus paralicheniformis*
GCF_002993945	*Bacillus paralicheniformis*
GCF_009497935	*Bacillus paralicheniformis*
GCF_003711025	*Bacillus paralicheniformis*
GCF_011068305	*Bacillus paralicheniformis*
GCF_002068155	*Bacillus paralicheniformis*
GCF_900635765	*Bacillus paralicheniformis*
GCF_018324565	*Bacillus paralicheniformis Strain: RSC-2*
GCF_000408885	*Bacillus paralicheniformis ATCC 9945a*
GCF_018324505	*Bacillus paralicheniformis Strain: RSC-1*
GCF_015334865	*Bacillus paranthracis*
GCF_015693885	*Bacillus paranthracis*
GCF_009648955	*Bacillus paranthracis*
GCF_016834515	*Bacillus paranthracis*
GCF_009799965	*Bacillus paranthracis*
GCF_010669045	*Bacillus paranthracis*
GCF_005848985	*Bacillus paranthracis*
GCF_018513785	*Bacillus paranthracis Strain: Colony502*
GCF_000161415	*Bacillus pseudomycoides*
GCF_000161435	*Bacillus pseudomycoides*
GCF_000742855	*Bacillus pseudomycoides*
GCF_000832885	*Bacillus pseudomycoides*
GCF_000161455	*Bacillus pseudomycoides DSM 12442*
GCF_014489355	*Bacillus pumilus*
GCF_001548215	*Bacillus pumilus*
GCF_001578165	*Bacillus pumilus*
GCF_001578205	*Bacillus pumilus*
GCF_001687085	*Bacillus pumilus*
GCF_003431975	*Bacillus pumilus*
GCF_001704975	*Bacillus pumilus*
GCF_003571425	*Bacillus pumilus*
GCF_009937765	*Bacillus pumilus*
GCF_000590455	*Bacillus pumilus*
GCF_004006455	*Bacillus pumilus*
GCF_013423765	*Bacillus pumilus*

GCF_900186955	*Bacillus pumilus*
GCF_000017885	*Bacillus pumilus SAFR-032*
GCF_008244765	*Bacillus safensis*
GCF_016803835	*Bacillus safensis*
GCF_001895885	*Bacillus safensis*
GCF_001938665	*Bacillus safensis*
GCF_001938685	*Bacillus safensis*
GCF_001938705	*Bacillus safensis*
GCF_002077215	*Bacillus safensis*
GCF_003660145	*Bacillus safensis Strain: sami*
GCF_003097715	*Bacillus safensis FO-36b*
GCF_002850535	*Bacillus siamensis*
GCF_016313165	*Bacillus siamensis*
GCF_001050115	*Bacillus smithii*
GCF_002202015	*Bacillus sonorensis*
GCF_000242895	*Bacillus sp. 1NLA3E*
GCF_002269275	*Bacillus sp. 1s-1*
GCF_001999205	*Bacillus sp. 275*
GCF_017161885	*Bacillus sp. 3a*
GCF_019464555	*Bacillus sp. 7D3*
GCF_009861435	*Bacillus sp. A260*
GCF_001831375	*Bacillus sp. ABP14*
GCF_009906915	*Bacillus sp. AM1(2019)*
GCF_008274865	*Bacillus sp. AR4-2*
GCF_001679765	*Bacillus sp. B25*
GCF_007363175	*Bacillus sp. BD59S*
GCF_000827045	*Bacillus sp. BH072*
GCF_008432245	*Bacillus sp. BS98*
GCF_003355115	*Bacillus sp. COPE52*
GCF_003429245	*Bacillus sp. CR71*
GCF_012955605	*Bacillus sp. CS13*
GCF_011082085	*Bacillus sp. Cs-700*
GCF_003330965	*Bacillus sp. DM2*
GCF_009720625	*Bacillus sp. DSL-17*
GCF_004799705	*Bacillus sp. DU-106*
GCF_003429225	*Bacillus sp. E25*
GCF_002073415	*Bacillus sp. FDAARGOS_235*
GCF_003812125	*Bacillus sp. FDAARGOS_527*
GCF_001719225	*Bacillus sp. FJAT-14266*
GCF_001278805	*Bacillus sp. FJAT-18017*
GCF_001278755	*Bacillus sp. FJAT-22090*
GCF_003816055	*Bacillus sp. FJAT-42376*
GCF_002813025	*Bacillus sp. FJAT-45348*
GCF_001896025	*Bacillus sp. H15-1*

GCF_002833485	*Bacillus sp. HBCD-sjtu*
GCF_014960805	*Bacillus sp. HD4P25*
GCF_012572025	*Bacillus sp. HNA3*
GCF_001648135	*Bacillus sp. IHB B 7164*
GCF_008274905	*Bacillus sp. JAS24-2*
GCF_018861275	*Bacillus sp. JNUCC-22*
GCF_018885385	*Bacillus sp. JNUCC-24*
GCF_000259365	*Bacillus sp. JS*
GCF_005937795	*Bacillus sp. KBS0812*
GCF_011398925	*Bacillus sp. KH172YL63*
GCF_016924995	*Bacillus sp. LJBS06*
GCF_019334685	*Bacillus sp. LJBS17*
GCF_017797845	*Bacillus sp. LJBV19*
GCF_000978495	*Bacillus sp. LM 4-2*
GCF_011008975	*Bacillus sp. LUNF1*
GCF_002763675	*Bacillus sp. Lzh-5*
GCF_006965545	*Bacillus sp. M4U3P1*
GCF_002953915	*Bacillus sp. MBGLi79*
GCF_002201875	*Bacillus sp. MD-5*
GCF_009759805	*Bacillus sp. ms-22*
GCF_009818105	*Bacillus sp. N1-1*
GCF_009676825	*Bacillus sp. N3536*
GCF_018889975	*Bacillus sp. NP157*
GCF_000465465	*Bacillus sp. NSP9.1*
GCF_000829195	*Bacillus sp. OxB-1*
GCF_014302175	*Bacillus sp. PAMC22265*
GCF_014250375	*Bacillus sp. PAMC26543*
GCF_014235785	*Bacillus sp. PAMC26568*
GCF_014250435	*Bacillus sp. PAMC28571*
GCF_014250415	*Bacillus sp. PAMC28748*
GCF_000815145	*Bacillus sp. Pc3*
GCF_016919285	*Bacillus sp. PDNC022*
GCF_001816185	*Bacillus sp. RZ2MS9*
GCF_005154805	*Bacillus sp. S3*
GCF_000469135	*Bacillus sp. SB49*
GCF_001553895	*Bacillus sp. SDLI1*
GCF_008274885	*Bacillus sp. SH8-8*
GCF_002843505	*Bacillus sp. SJ-10*
GCF_003171815	*Bacillus sp. SUBG0010*
GCF_004322655	*Bacillus sp. SYJ*
GCF_009363695	*Bacillus sp. THAF10*
GCF_016653575	*Bacillus sp. TK-2*
GCF_015999585	*Bacillus sp. V3*
GCF_000800825	*Bacillus sp. WP8*

GCF_004103875	*Bacillus sp. WR11*
GCF_000747345	*Bacillus sp. X1(2014)*
GCF_003047225	*Bacillus sp. Y-01*
GCF_003586445	*Bacillus sp. Y1*
GCF_000877815	*Bacillus sp. YP1*
GCF_003047165	*Bacillus sp. ZY-1-1*
GCF_002269175	*Bacillus subtilis*
GCF_002269195	*Bacillus subtilis*
GCF_006165085	*Bacillus subtilis*
GCF_006364495	*Bacillus subtilis*
GCF_000952895	*Bacillus subtilis*
GCF_000953615	*Bacillus subtilis*
GCF_002290305	*Bacillus subtilis*
GCF_014879275	*Bacillus subtilis*
GCF_001015095	*Bacillus subtilis*
GCF_001037985	*Bacillus subtilis*
GCF_015535655	*Bacillus subtilis*
GCF_015602625	*Bacillus subtilis*
GCF_015654205	*Bacillus subtilis*
GCF_002893805	*Bacillus subtilis*
GCF_008831405	*Bacillus subtilis*
GCF_016065415	*Bacillus subtilis*
GCF_009017455	*Bacillus subtilis*
GCF_002982175	*Bacillus subtilis*
GCF_009363835	*Bacillus subtilis*
GCF_009497795	*Bacillus subtilis*
GCF_009497815	*Bacillus subtilis*
GCF_009662155	*Bacillus subtilis*
GCF_009662175	*Bacillus subtilis*
GCF_009662195	*Bacillus subtilis*
GCF_009662215	*Bacillus subtilis*
GCF_009662235	*Bacillus subtilis*
GCF_001604995	*Bacillus subtilis*
GCF_009662255	*Bacillus subtilis*
GCF_009662275	*Bacillus subtilis*
GCF_009662335	*Bacillus subtilis*
GCF_003148355	*Bacillus subtilis*
GCF_009662355	*Bacillus subtilis*
GCF_009662375	*Bacillus subtilis*
GCF_009662415	*Bacillus subtilis*
GCF_009662435	*Bacillus subtilis*
GCF_009662455	*Bacillus subtilis*
GCF_016894165	*Bacillus subtilis*
GCF_003184225	*Bacillus subtilis*

GCF_009665145	*Bacillus subtilis*
GCF_009676665	*Bacillus subtilis*
GCF_001704095	*Bacillus subtilis*
GCF_001720505	*Bacillus subtilis*
GCF_009857115	*Bacillus subtilis*
GCF_009866865	*Bacillus subtilis*
GCF_009884005	*Bacillus subtilis*
GCF_001746575	*Bacillus subtilis*
GCF_009913255	*Bacillus subtilis*
GCF_001747445	*Bacillus subtilis*
GCF_009913275	*Bacillus subtilis*
GCF_003610955	*Bacillus subtilis*
GCF_001808235	*Bacillus subtilis*
GCF_009913395	*Bacillus subtilis*
GCF_009913515	*Bacillus subtilis*
GCF_009913535	*Bacillus subtilis*
GCF_009913555	*Bacillus subtilis*
GCF_009913575	*Bacillus subtilis*
GCF_009913595	*Bacillus subtilis*
GCF_001889385	*Bacillus subtilis*
GCF_001889625	*Bacillus subtilis*
GCF_009914155	*Bacillus subtilis*
GCF_001890405	*Bacillus subtilis*
GCF_009937745	*Bacillus subtilis*
GCF_001902555	*Bacillus subtilis*
GCF_011765645	*Bacillus subtilis*
GCF_004101345	*Bacillus subtilis*
GCF_004101365	*Bacillus subtilis*
GCF_004101405	*Bacillus subtilis*
GCF_004101425	*Bacillus subtilis*
GCF_004101445	*Bacillus subtilis*
GCF_002055965	*Bacillus subtilis*
GCF_004101465	*Bacillus subtilis*
GCF_004101485	*Bacillus subtilis*
GCF_004101565	*Bacillus subtilis*
GCF_002072735	*Bacillus subtilis*
GCF_004101945	*Bacillus subtilis*
GCF_004103535	*Bacillus subtilis*
GCF_012647565	*Bacillus subtilis*
GCF_004103555	*Bacillus subtilis*
GCF_004103595	*Bacillus subtilis*
GCF_002096095	*Bacillus subtilis*
GCF_000769515	*Bacillus subtilis*
GCF_004119535	*Bacillus subtilis*

GCF_000772125	*Bacillus subtilis*
GCF_004119555	*Bacillus subtilis*
GCF_000772165	*Bacillus subtilis*
GCF_000772205	*Bacillus subtilis*
GCF_004119595	*Bacillus subtilis*
GCF_002142595	*Bacillus subtilis*
GCF_004119615	*Bacillus subtilis*
GCF_000782835	*Bacillus subtilis*
GCF_002163815	*Bacillus subtilis*
GCF_004119635	*Bacillus subtilis*
GCF_004119655	*Bacillus subtilis*
GCF_000789295	*Bacillus subtilis*
GCF_004119675	*Bacillus subtilis*
GCF_004119695	*Bacillus subtilis*
GCF_004119715	*Bacillus subtilis*
GCF_004119775	*Bacillus subtilis*
GCF_004119815	*Bacillus subtilis*
GCF_004119835	*Bacillus subtilis*
GCF_004119875	*Bacillus subtilis*
GCF_013285325	*Bacillus subtilis*
GCF_002202055	*Bacillus subtilis*
GCF_002205435	*Bacillus subtilis*
GCF_013347105	*Bacillus subtilis*
GCF_002216085	*Bacillus subtilis*
GCF_013388335	*Bacillus subtilis*
GCF_002220075	*Bacillus subtilis*
GCF_013393725	*Bacillus subtilis*
GCF_005153965	*Bacillus subtilis*
GCF_005160425	*Bacillus subtilis*
GCF_005234095	*Bacillus subtilis*
GCF_005849145	*Bacillus subtilis*
GCF_014171535	*Bacillus subtilis*
GCF_018394175	*Bacillus subtilis Strain: BYS2*
GCF_018141065	*Bacillus subtilis Strain: PMB102*
GCF_018224865	*Bacillus subtilis Strain: SC3.7*
GCF_003515125	*Bacillus subtilis Strain: FB6-3*
GCF_019464335	*Bacillus subtilis Strain: HD15*
GCF_018884165	*Bacillus subtilis Strain: s-16*
GCF_017753785	*Bacillus subtilis Strain: YB-04*
GCF_000523045	*Bacillus subtilis BEST7003*
GCF_000186745	*Bacillus subtilis BSn5*
GCF_000973605	*Bacillus subtilis HJ5*
GCF_000971925	*Bacillus subtilis KCTC 1028 = ATCC 6051a*
GCF_000497485	*Bacillus subtilis PY79*

GCF_000293765	*Bacillus subtilis QB928*
GCF_019599105	*Bacillus subtilis Strain: YPS-32*
GCF_001597265	*Bacillus subtilis subsp. globigii*
GCF_001565875	*Bacillus subtilis subsp. natto*
GCF_000209795	*Bacillus subtilis subsp. natto BEST195*
GCF_006094475	*Bacillus subtilis subsp. spizizenii ATCC 6633*
GCF_000146565	*Bacillus subtilis subsp. spizizenii str. W23*
GCF_000227465	*Bacillus subtilis subsp. spizizenii TU-B-10*
GCF_014298115	*Bacillus subtilis subsp. subtilis*
GCF_006741845	*Bacillus subtilis subsp. subtilis*
GCF_014961985	*Bacillus subtilis subsp. subtilis*
GCF_001465815	*Bacillus subtilis subsp. subtilis*
GCF_001541905	*Bacillus subtilis subsp. subtilis*
GCF_001660525	*Bacillus subtilis subsp. subtilis*
GCF_003426125	*Bacillus subtilis subsp. subtilis*
GCF_001697265	*Bacillus subtilis subsp. subtilis*
GCF_001703495	*Bacillus subtilis subsp. subtilis*
GCF_001750745	*Bacillus subtilis subsp. subtilis*
GCF_001750765	*Bacillus subtilis subsp. subtilis*
GCF_003665195	*Bacillus subtilis subsp. subtilis*
GCF_003665215	*Bacillus subtilis subsp. subtilis*
GCF_003665235	*Bacillus subtilis subsp. subtilis*
GCF_003665255	*Bacillus subtilis subsp. subtilis*
GCF_003665275	*Bacillus subtilis subsp. subtilis*
GCF_003665295	*Bacillus subtilis subsp. subtilis*
GCF_003665315	*Bacillus subtilis subsp. subtilis*
GCF_003665335	*Bacillus subtilis subsp. subtilis*
GCF_003665355	*Bacillus subtilis subsp. subtilis*
GCF_003665395	*Bacillus subtilis subsp. subtilis*
GCF_012648205	*Bacillus subtilis subsp. subtilis*
GCF_012648345	*Bacillus subtilis subsp. subtilis*
GCF_002173615	*Bacillus subtilis subsp. subtilis*
GCF_002173695	*Bacillus subtilis subsp. subtilis*
GCF_002173715	*Bacillus subtilis subsp. subtilis*
GCF_000827065	*Bacillus subtilis subsp. subtilis*
GCF_002201955	*Bacillus subtilis subsp. subtilis*
GCF_002201995	*Bacillus subtilis subsp. subtilis*
GCF_002202035	*Bacillus subtilis subsp. subtilis*
GCF_004328925	*Bacillus subtilis subsp. subtilis*
GCF_018454405	*Bacillus subtilis subsp. subtilis Strain: A1 - Midalam*
GCF_000344745	*Bacillus subtilis subsp. subtilis 6051-HGW*
GCF_006088795	*Bacillus subtilis subsp. subtilis NCIB 3610 = ATCC 6051*
GCF_000186085	*Bacillus subtilis subsp. subtilis NCIB 3610 = ATCC 6051*
GCF_000009045	*Bacillus subtilis subsp. subtilis str. 168*

GCF_000155325	*Bacillus subtilis subsp. subtilis str. 168*
GCF_002009095	*Bacillus subtilis subsp. subtilis str. 168*
GCF_002009135	*Bacillus subtilis subsp. subtilis str. 168*
GCF_012931705	*Bacillus subtilis subsp. subtilis str. 168*
GCF_013009385	*Bacillus subtilis subsp. subtilis str. 168*
GCF_000789275	*Bacillus subtilis subsp. subtilis str. 168*
GCF_000699525	*Bacillus subtilis subsp. subtilis str. AG1839*
GCF_000349795	*Bacillus subtilis subsp. subtilis str. BAB-1*
GCF_000321395	*Bacillus subtilis subsp. subtilis str. BSP1*
GCF_000155355	*Bacillus subtilis subsp. subtilis str. JH642*
GCF_000699465	*Bacillus subtilis subsp. subtilis str. JH642 substr. AG174*
GCF_000706705	*Bacillus subtilis subsp. subtilis str. OH 131.1*
GCF_000227485	*Bacillus subtilis subsp. subtilis str. RO-NN-1*
GCF_000155375	*Bacillus subtilis subsp. subtilis str. SMY*
GCF_012030675	*Bacillus subtilis subsp. subtilis str. SMY*
GCF_018986855	*Bacillus subtilis subsp. subtilisStrain: Miz-8*
GCF_000737405	*Bacillus subtilis TO-A*
GCF_000338735	*Bacillus subtilis XF-1*
GCF_012225885	*Bacillus tequilensis*
GCF_006151925	*Bacillus thuringiensis*
GCF_001017635	*Bacillus thuringiensis*
GCF_001182785	*Bacillus thuringiensis*
GCF_001420855	*Bacillus thuringiensis*
GCF_001455345	*Bacillus thuringiensis*
GCF_009025915	*Bacillus thuringiensis*
GCF_016070565	*Bacillus thuringiensis*
GCF_009224065	*Bacillus thuringiensis*
GCF_001595725	*Bacillus thuringiensis*
GCF_003054785	*Bacillus thuringiensis*
GCF_001598095	*Bacillus thuringiensis*
GCF_001618665	*Bacillus thuringiensis*
GCF_001685565	*Bacillus thuringiensis*
GCF_001692675	*Bacillus thuringiensis*
GCF_003626955	*Bacillus thuringiensis*
GCF_003991175	*Bacillus thuringiensis*
GCF_002025105	*Bacillus thuringiensis*
GCF_002119445	*Bacillus thuringiensis*
GCF_013267295	*Bacillus thuringiensis*
GCF_002173755	*Bacillus thuringiensis*
GCF_013267315	*Bacillus thuringiensis*
GCF_013267335	*Bacillus thuringiensis*
GCF_002184245	*Bacillus thuringiensis*
GCF_002192515	*Bacillus thuringiensis*
GCF_013267795	*Bacillus thuringiensis*

GCF_013267815	*Bacillus thuringiensis*
GCF_004194435	*Bacillus thuringiensis*
GCF_004194455	*Bacillus thuringiensis*
GCF_004194475	*Bacillus thuringiensis*
GCF_000832485	*Bacillus thuringiensis*
GCF_013340745	*Bacillus thuringiensis*
GCF_013343075	*Bacillus thuringiensis*
GCF_004354265	*Bacillus thuringiensis*
GCF_000832825	*Bacillus thuringiensis*
GCF_002222555	*Bacillus thuringiensis*
GCF_005155285	*Bacillus thuringiensis*
GCF_000832925	*Bacillus thuringiensis*
GCF_000833085	*Bacillus thuringiensis*
GCF_017751245	*Bacillus thuringiensis Strain: Bt-GS57*
GCF_017378115	*Bacillus thuringiensis Strain: TAND672*
GCF_000092165	*Bacillus thuringiensis BMB171*
GCF_001721165	*Bacillus thuringiensis Bt18247*
GCF_000161495	*Bacillus thuringiensis Bt407*
GCF_000306745	*Bacillus thuringiensis Bt407*
GCF_000342025	*Bacillus thuringiensis DAR 81934*
GCF_000835025	*Bacillus thuringiensis HD1002*
GCF_000292455	*Bacillus thuringiensis HD-771*
GCF_000292705	*Bacillus thuringiensis HD-789*
GCF_000161715	*Bacillus thuringiensis IBL 200*
GCF_000161735	*Bacillus thuringiensis IBL 4222*
GCF_003546665	*Bacillus thuringiensis LM1212*
GCF_000300475	*Bacillus thuringiensis MC28*
GCF_001640965	*Bacillus thuringiensis serovar alesti*
GCF_012222085	*Bacillus thuringiensis serovar andalousiensis*
GCF_000161635	*Bacillus thuringiensis serovar andalousiensis BGSC 4AW1*
GCF_000161615	*Bacillus thuringiensis serovar berliner ATCC 10792*
GCF_000193355	*Bacillus thuringiensis serovar chinensis CT-43*
GCF_000803665	*Bacillus thuringiensis serovar galleriae*
GCF_000161675	*Bacillus thuringiensis serovar huazhongensis BGSC 4BD1*
GCF_001183785	*Bacillus thuringiensis serovar indiana*
GCF_003445395	*Bacillus thuringiensis serovar israelensis*
GCF_013113775	*Bacillus thuringiensis serovar israelensis*
GCF_000835235	*Bacillus thuringiensis serovar kurstaki*
GCF_000717535	*Bacillus thuringiensis serovar kurstaki str. HD-1*
GCF_000338755	*Bacillus thuringiensis serovar kurstaki str. HD73*
GCF_000161575	*Bacillus thuringiensis serovar kurstaki str. T03a001*
GCF_000688795	*Bacillus thuringiensis serovar kurstaki str. YBT-1520*

GCF_000747545	*Bacillus thuringiensis serovar kurstaki str. YBT-1520*
GCF_000161595	*Bacillus thuringiensis serovar monterrey BGSC 4AJ1*
GCF_000940785	*Bacillus thuringiensis serovar morrisoni*
GCF_000161555	*Bacillus thuringiensis serovar pakistani str. T13001*
GCF_000161655	*Bacillus thuringiensis serovar pondicheriensis BGSC 4BA1*
GCF_000161695	*Bacillus thuringiensis serovar pulsiensis BGSC 4CC1*
GCF_000341665	*Bacillus thuringiensis serovar thuringiensis str. IS5056*
GCF_000161515	*Bacillus thuringiensis serovar thuringiensis str. T01001*
GCF_001548175	*Bacillus thuringiensis serovar tolworthi*
GCF_000015065	*Bacillus thuringiensis str. Al Hakam*
GCF_018885365	*Bacillus thuringiensis Strain: GR007*
GCF_000497525	*Bacillus thuringiensis YBT-1518*
GCF_016605985	*Bacillus toyonensis*
GCF_018739605	*Bacillus toyonensis Strain: BPN45/4*
GCF_018741125	*Bacillus toyonensis Strain: JAS03/3*
GCF_018741545	*Bacillus toyonensis Strain: JAS22/1*
GCF_018741525	*Bacillus toyonensis Strain: JAS13/1*
GCF_000496285	*Bacillus toyonensis BCT-7112*
GCF_018741625	*Bacillus toyonensis Strain: JAS411*
GCF_016026855	*Bacillus tropicus*
GCF_016027575	*Bacillus tropicus*
GCF_011040455	*Bacillus tropicus*
GCF_013331815	*Bacillus tropicus*
GCF_018141085	*Bacillus tropicus Strain: LM1212-DB*
GCF_003667885	*Bacillus vallismortis*
GCF_004116955	*Bacillus vallismortis*
GCF_002113805	*Bacillus vallismortis*
GCF_000875875	*Bacillus velezensis*
GCF_014358035	*Bacillus velezensis*
GCF_002290045	*Bacillus velezensis*
GCF_006489235	*Bacillus velezensis*
GCF_014495825	*Bacillus velezensis*
GCF_006489255	*Bacillus velezensis*
GCF_006489275	*Bacillus velezensis*
GCF_014622705	*Bacillus velezensis*
GCF_002310315	*Bacillus velezensis*
GCF_002327165	*Bacillus velezensis*
GCF_000973485	*Bacillus velezensis*
GCF_014706595	*Bacillus velezensis*
GCF_014764325	*Bacillus velezensis*
GCF_006965525	*Bacillus velezensis*
GCF_014841075	*Bacillus velezensis*
GCF_000987825	*Bacillus velezensis*

GCF_006974185	*Bacillus velezensis*
GCF_000988345	*Bacillus velezensis*
GCF_002556565	*Bacillus velezensis*
GCF_002688525	*Bacillus velezensis*
GCF_001023595	*Bacillus velezensis*
GCF_002741705	*Bacillus velezensis*
GCF_014961825	*Bacillus velezensis*
GCF_002761535	*Bacillus velezensis*
GCF_002764075	*Bacillus velezensis*
GCF_015239615	*Bacillus velezensis*
GCF_002796465	*Bacillus velezensis*
GCF_015277495	*Bacillus velezensis*
GCF_015291865	*Bacillus velezensis*
GCF_008244925	*Bacillus velezensis*
GCF_002844125	*Bacillus velezensis*
GCF_002845365	*Bacillus velezensis*
GCF_002850475	*Bacillus velezensis*
GCF_008367895	*Bacillus velezensis*
GCF_015732475	*Bacillus velezensis*
GCF_002893845	*Bacillus velezensis*
GCF_002944585	*Bacillus velezensis*
GCF_008727675	*Bacillus velezensis*
GCF_008802875	*Bacillus velezensis*
GCF_001536925	*Bacillus velezensis*
GCF_002968415	*Bacillus velezensis*
GCF_016065635	*Bacillus velezensis*
GCF_002993245	*Bacillus velezensis*
GCF_009362235	*Bacillus velezensis*
GCF_003045165	*Bacillus velezensis*
GCF_016406185	*Bacillus velezensis*
GCF_001593395	*Bacillus velezensis*
GCF_003047005	*Bacillus velezensis*
GCF_016415725	*Bacillus velezensis*
GCF_003047025	*Bacillus velezensis*
GCF_003047045	*Bacillus velezensis*
GCF_016766595	*Bacillus velezensis*
GCF_003071465	*Bacillus velezensis*
GCF_003073255	*Bacillus velezensis*
GCF_003073455	*Bacillus velezensis*
GCF_003076535	*Bacillus velezensis*
GCF_016801735	*Bacillus velezensis*
GCF_016834435	*Bacillus velezensis*
GCF_016859395	*Bacillus velezensis*
GCF_016888865	*Bacillus velezensis*

GCF_003150855	*Bacillus velezensis*
GCF_009663035	*Bacillus velezensis*
GCF_016904925	*Bacillus velezensis*
GCF_009664145	*Bacillus velezensis*
GCF_003184945	*Bacillus velezensis*
GCF_003265265	*Bacillus velezensis*
GCF_003285085	*Bacillus velezensis*
GCF_009738165	*Bacillus velezensis*
GCF_003390415	*Bacillus velezensis*
GCF_001685645	*Bacillus velezensis*
GCF_009789615	*Bacillus velezensis*
GCF_001687745	*Bacillus velezensis*
GCF_003431885	*Bacillus velezensis*
GCF_003432425	*Bacillus velezensis*
GCF_003441635	*Bacillus velezensis*
GCF_009834085	*Bacillus velezensis*
GCF_003546955	*Bacillus velezensis*
GCF_009856975	*Bacillus velezensis*
GCF_003555525	*Bacillus velezensis*
GCF_001723585	*Bacillus velezensis*
GCF_003573875	*Bacillus velezensis*
GCF_009913295	*Bacillus velezensis*
GCF_003612755	*Bacillus velezensis*
GCF_009913315	*Bacillus velezensis*
GCF_001752685	*Bacillus velezensis*
GCF_009913335	*Bacillus velezensis*
GCF_009913355	*Bacillus velezensis*
GCF_009913375	*Bacillus velezensis*
GCF_001854345	*Bacillus velezensis*
GCF_009913475	*Bacillus velezensis*
GCF_009913495	*Bacillus velezensis*
GCF_001857985	*Bacillus velezensis*
GCF_009914135	*Bacillus velezensis*
GCF_003667745	*Bacillus velezensis*
GCF_009914175	*Bacillus velezensis*
GCF_009931675	*Bacillus velezensis*
GCF_003721475	*Bacillus velezensis*
GCF_003854915	*Bacillus velezensis*
GCF_003855275	*Bacillus velezensis*
GCF_003860445	*Bacillus velezensis*
GCF_011032725	*Bacillus velezensis*
GCF_011100445	*Bacillus velezensis*
GCF_011769925	*Bacillus velezensis*
GCF_002005345	*Bacillus velezensis*

GCF_011784665	*Bacillus velezensis*
GCF_012226065	*Bacillus velezensis*
GCF_002057535	*Bacillus velezensis*
GCF_002072695	*Bacillus velezensis*
GCF_004101805	*Bacillus velezensis*
GCF_012275165	*Bacillus velezensis*
GCF_012647845	*Bacillus velezensis*
GCF_002105595	*Bacillus velezensis*
GCF_000769555	*Bacillus velezensis*
GCF_002117165	*Bacillus velezensis*
GCF_004119575	*Bacillus velezensis*
GCF_013122275	*Bacillus velezensis*
GCF_013201135	*Bacillus velezensis*
GCF_013201465	*Bacillus velezensis*
GCF_004119735	*Bacillus velezensis*
GCF_000815275	*Bacillus velezensis*
GCF_002192235	*Bacillus velezensis*
GCF_004119855	*Bacillus velezensis*
GCF_004135455	*Bacillus velezensis*
GCF_002205715	*Bacillus velezensis*
GCF_004329055	*Bacillus velezensis*
GCF_004331895	*Bacillus velezensis*
GCF_004337655	*Bacillus velezensis*
GCF_013348705	*Bacillus velezensis*
GCF_013367755	*Bacillus velezensis*
GCF_002216755	*Bacillus velezensis*
GCF_004799565	*Bacillus velezensis*
GCF_013401395	*Bacillus velezensis*
GCF_013694265	*Bacillus velezensis*
GCF_002237515	*Bacillus velezensis*
GCF_013701805	*Bacillus velezensis*
GCF_002238395	*Bacillus velezensis*
GCF_013875015	*Bacillus velezensis*
GCF_002243325	*Bacillus velezensis*
GCF_005696395	*Bacillus velezensis*
GCF_000833005	*Bacillus velezensis*
GCF_014048285	*Bacillus velezensis*
GCF_014048385	*Bacillus velezensis*
GCF_014117505	*Bacillus velezensis*
GCF_014180705	*Bacillus velezensis*
GCF_014211995	*Bacillus velezensis*
GCF_017347625	*Bacillus velezensis Strain: AD-3*
GCF_017742975	*Bacillus velezensis Strain: AD8*
GCF_019222745	*Bacillus velezensis Strain: BS-G1*

GCF_009734085	*Bacillus velezensis Strain: GA1*
GCF_019334525	*Bacillus velezensis Strain: LABIM44*
GCF_019285615	*Bacillus velezensis Strain: LBY-1*
GCF_018415955	*Bacillus velezensis Strain: VTX20*
GCF_017948225	*Bacillus velezensis Strain: VY03*
GCF_019464595	*Bacillus velezensis Strain: WB*
GCF_018223705	*Bacillus velezensis Strain: Y816*
GCF_019163475	*Bacillus velezensis Strain: YYC*
GCF_019551675	*Bacillus velezensis Strain: 19573-3*
GCF_018866365	*Bacillus velezensis Strain: GMEKP1*
GCF_018775385	*Bacillus velezensis Strain: J17-4*
GCF_018406485	*Bacillus velezensis Strain: KOF112*
GCF_018732185	*Bacillus velezensis Strain: NZ4*
GCF_018771705	*Bacillus velezensis Strain: US1*
GCF_018629015	*Bacillus velezensis Strain: VCN56*
GCF_018398955	*Bacillus velezensis Strain: VTX9*
GCF_000319475	*Bacillus velezensis AS43.3*
GCF_000283695	*Bacillus velezensis CAU B946*
GCF_000015785	*Bacillus velezensis FZB42*
GCF_000493375	*Bacillus velezensis NAU-B3*
GCF_000973585	*Bacillus velezensis NJN-6*
GCF_000685725	*Bacillus velezensis SQR9*
GCF_019449395	*Bacillus velezensis Strain: GS-1*
GCF_018278845	*Bacillus velezensis Strain: JK19*
GCF_018771665	*Bacillus velezensis Strain: Q12*
GCF_000583065	*Bacillus velezensis TrigoCor1448*
GCF_000455565	*Bacillus velezensis UCMB5033*
GCF_000341875	*Bacillus velezensis UCMB5036*
GCF_000455585	*Bacillus velezensis UCMB5113*
GCF_000284395	*Bacillus velezensis YAU B9601-Y2*
GCF_001889165	*Bacillus weihaiensis*
GCF_000160955	*Bacillus wiedmannii*
GCF_008807735	*Bacillus wiedmannii*
GCF_018741285	*Bacillus wiedmannii Strain: JAS07/5*
GCF_018741405	*Bacillus wiedmannii Strain: JAS08/1*
GCF_018741585	*Bacillus wiedmannii Strain: JAS28/2*
GCF_001857925	*Bacillus xiamenensis*

Table S2. Number of *SrfAA*, *SrfAB* and *SrfAC* genes in 999 Bacillus species genomes.

**Accession Number**	**Species**	***SrfAA***	***SrfAB***	***SrfAC***
GCF_004101925	*Bacillus aerophilus*	2	3	6
GCF_009740005	*Bacillus albus*	8	5	8
GCF_000972685	*Bacillus altitudinis*	4	4	7
GCF_002443015	*Bacillus altitudinis*	7	4	6
GCF_002741745	*Bacillus altitudinis*	4	4	5
GCF_007923025	*Bacillus altitudinis*	7	5	3
GCF_015160895	*Bacillus altitudinis*	5	4	5
GCF_001191605	*Bacillus altitudinis*	4	3	4
GCF_001431145	*Bacillus altitudinis*	3	4	5
GCF_001431785	*Bacillus altitudinis*	0	0	0
GCF_001543165	*Bacillus altitudinis*	4	3	4
GCF_016767855	*Bacillus altitudinis*	3	4	5
GCF_016807685	*Bacillus altitudinis*	3	4	7
GCF_001700735	*Bacillus altitudinis*	0	0	0
GCF_000299555	*Bacillus altitudinis*	4	4	7
GCF_001908475	*Bacillus altitudinis*	2	2	1
GCF_011067205	*Bacillus altitudinis*	2	4	5
GCF_013283915	*Bacillus altitudinis*	6	5	4
GCF_013307105	*Bacillus altitudinis*	1	1	1
GCF_004563755	*Bacillus altitudinis*	5	4	7

GCF_005849435	*Bacillus altitudinis*	6	4	7
GCF_006007905	*Bacillus altitudinis*	6	2	4
GCF_017948365	*Bacillus altitudinis Strain: 6ww6*	4	4	2
GCF_015689015	*Bacillus altitudinis Strain: Ba1449*	2	3	3
GCF_017161205	*Bacillus altitudinis Strain: 1817*	3	4	5
GCF_019164215	*Bacillus altitudinis Strain: LZP 02*	5	4	7
GCF_019355135	*Bacillus altitudinis Strain: SCU11*	1	1	0
GCF_014622745	*Bacillus amyloliquefaciens*	0	0	1
GCF_014791945	*Bacillus amyloliquefaciens*	1	0	0
GCF_014792065	*Bacillus amyloliquefaciens*	16	8	5
GCF_014854735	*Bacillus amyloliquefaciens*	11	11	8
GCF_007362635	*Bacillus amyloliquefaciens*	2	0	0
GCF_014930895	*Bacillus amyloliquefaciens*	14	12	10
GCF_007827165	*Bacillus amyloliquefaciens*	0	0	0
GCF_007923205	*Bacillus amyloliquefaciens*	11	16	10
GCF_008534375	*Bacillus amyloliquefaciens*	1	2	0
GCF_015999565	*Bacillus amyloliquefaciens*	0	0	0
GCF_001483885	*Bacillus amyloliquefaciens*	8	17	9
GCF_008808015	*Bacillus amyloliquefaciens*	5	0	1
GCF_008824205	*Bacillus amyloliquefaciens*	8	13	5
GCF_001586105	*Bacillus amyloliquefaciens*	1	1	0
GCF_001596755	*Bacillus amyloliquefaciens*	12	12	11

GCF_003149 695	*Bacillus amyloliquefaciens*	6	4	0
GCF_003149 715	*Bacillus amyloliquefaciens*	13	14	12
GCF_003149 795	*Bacillus amyloliquefaciens*	4	3	0
GCF_003312 895	*Bacillus amyloliquefaciens*	10	12	5
GCF_001705 195	*Bacillus amyloliquefaciens*	10	7	11
GCF_001874 385	*Bacillus amyloliquefaciens*	9	15	10
GCF_001889 285	*Bacillus amyloliquefaciens*	9	12	7
GCF_001922 005	*Bacillus amyloliquefaciens*	1	0	0
GCF_003868 675	*Bacillus amyloliquefaciens*	11	10	10
GCF_004006 115	*Bacillus amyloliquefaciens*	0	0	0
GCF_013122 255	*Bacillus amyloliquefaciens*	1	2	0
GCF_002173 635	*Bacillus amyloliquefaciens*	2	1	1
GCF_013328 835	*Bacillus amyloliquefaciens*	4	3	2
GCF_013341 255	*Bacillus amyloliquefaciens*	14	14	4
GCF_002209 305	*Bacillus amyloliquefaciens*	10	7	10
GCF_004421 045	*Bacillus amyloliquefaciens*	1	0	0
GCF_019396 925	*Bacillus amyloliquefaciens Strain: GKT04*	1	1	0
GCF_018223 605	*Bacillus amyloliquefaciens Strain: Ba13*	7	8	10
GCF_017357 905	*Bacillus amyloliquefaciens Strain: GXU-1*	5	5	2
GCF_018363 035	*Bacillus amyloliquefaciens Strain: L-17*	0	0	0
GCF_018502 525	*Bacillus amyloliquefaciens Strain: SN16-1*	0	0	0
GCF_000494 835	*Bacillus amyloliquefaciens CC178*	2	0	0

GCF_000196735	*Bacillus amyloliquefaciens DSM 7 = ATCC 23350*	8	7	5
GCF_000242855	*Bacillus amyloliquefaciens IT-45*	10	16	13
GCF_000835145	*Bacillus amyloliquefaciens KHG19*	14	11	10
GCF_000508265	*Bacillus amyloliquefaciens LFB112*	15	17	8
GCF_000204275	*Bacillus amyloliquefaciens LL3*	4	2	4
GCF_017815555	*Bacillus amyloliquefaciens Strain: G10*	0	0	0
GCF_017603845	*Bacillus amyloliquefaciens Strain: XJ5*	7	4	2
GCF_000195515	*Bacillus amyloliquefaciens TA208*	7	6	11
GCF_001593785	*Bacillus amyloliquefaciens UMAF6614*	16	11	10
GCF_001593765	*Bacillus amyloliquefaciens UMAF6639*	16	15	9
GCF_000221645	*Bacillus amyloliquefaciens XH7*	1	0	0
GCF_000262385	*Bacillus amyloliquefaciens Y2*	13	16	12
GCF_006088855	*Bacillus anthracis*	0	0	0
GCF_014249775	*Bacillus anthracis*	0	0	1
GCF_000875715	*Bacillus anthracis*	0	0	0
GCF_002277915	*Bacillus anthracis*	0	0	0
GCF_002356575	*Bacillus anthracis*	0	1	0
GCF_006742565	*Bacillus anthracis*	0	0	0
GCF_001277955	*Bacillus anthracis*	1	0	0
GCF_001543225	*Bacillus anthracis*	0	0	0
GCF_002980615	*Bacillus anthracis*	0	0	0
GCF_001654475	*Bacillus anthracis*	1	0	0

GCF_001683 065	*Bacillus anthracis*	0	0	0
GCF_001683 095	*Bacillus anthracis*	0	0	0
GCF_001683 135	*Bacillus anthracis*	0	0	0
GCF_003227 955	*Bacillus anthracis*	1	0	0
GCF_001683 155	*Bacillus anthracis*	0	0	0
GCF_001683 175	*Bacillus anthracis*	0	0	0
GCF_001683 195	*Bacillus anthracis*	0	0	0
GCF_001683 215	*Bacillus anthracis*	0	0	0
GCF_001683 235	*Bacillus anthracis*	0	0	0
GCF_001683 255	*Bacillus anthracis*	0	0	0
GCF_001683 275	*Bacillus anthracis*	0	0	0
GCF_001683 295	*Bacillus anthracis*	0	0	0
GCF_003410 255	*Bacillus anthracis*	4	2	5
GCF_003410 355	*Bacillus anthracis*	6	1	7
GCF_009806 595	*Bacillus anthracis*	0	0	0
GCF_009806 615	*Bacillus anthracis*	0	0	0
GCF_009806 635	*Bacillus anthracis*	0	0	0
GCF_009806 655	*Bacillus anthracis*	0	0	0
GCF_009806 675	*Bacillus anthracis*	0	0	0
GCF_009806 695	*Bacillus anthracis*	0	0	0
GCF_001936 375	*Bacillus anthracis*	1	0	0
GCF_000534 935	*Bacillus anthracis*	2	0	0

GCF_001990245	*Bacillus anthracis*	0	0	0
GCF_012273315	*Bacillus anthracis*	0	0	0
GCF_000725325	*Bacillus anthracis*	1	0	0
GCF_012273335	*Bacillus anthracis*	0	0	0
GCF_012273355	*Bacillus anthracis*	0	0	0
GCF_000742315	*Bacillus anthracis*	0	0	0
GCF_012273375	*Bacillus anthracis*	0	0	0
GCF_000742655	*Bacillus anthracis*	3	0	3
GCF_000742695	*Bacillus anthracis*	0	0	0
GCF_000742875	*Bacillus anthracis*	0	0	0
GCF_000831505	*Bacillus anthracis*	0	0	0
GCF_000832425	*Bacillus anthracis*	1	0	0
GCF_000832445	*Bacillus anthracis*	0	0	0
GCF_000832465	*Bacillus anthracis*	0	1	0
GCF_004328845	*Bacillus anthracis*	5	3	5
GCF_000832505	*Bacillus anthracis*	2	0	0
GCF_002208785	*Bacillus anthracis*	0	0	0
GCF_000832565	*Bacillus anthracis*	0	0	0
GCF_000832585	*Bacillus anthracis*	0	0	0
GCF_013349165	*Bacillus anthracis*	0	0	0
GCF_000832665	*Bacillus anthracis*	0	1	1
GCF_013349185	*Bacillus anthracis*	0	0	0

GCF_000832725	*Bacillus anthracis*	0	0	0
GCF_013349245	*Bacillus anthracis*	0	0	0
GCF_000832745	*Bacillus anthracis*	0	0	0
GCF_000832965	*Bacillus anthracis*	0	0	0
GCF_000833065	*Bacillus anthracis*	0	0	0
GCF_000833125	*Bacillus anthracis*	0	1	0
GCF_000559005	*Bacillus anthracis 52-G*	1	2	0
GCF_000558965	*Bacillus anthracis 8903-G*	1	0	0
GCF_000558985	*Bacillus anthracis 9080-G*	2	0	0
GCF_000022865	*Bacillus anthracis str. A0248*	0	0	0
GCF_000512835	*Bacillus anthracis str. A16*	0	0	0
GCF_000512775	*Bacillus anthracis str. A16R*	0	0	0
GCF_000007845	*Bacillus anthracis str. Ames*	5	2	9
GCF_000008445	*Bacillus anthracis str. 'Ames Ancestor'*	0	0	0
GCF_000295695	*Bacillus anthracis str. BF1*	2	1	1
GCF_000021445	*Bacillus anthracis str. CDC 684*	1	0	0
GCF_000258885	*Bacillus anthracis str. H9401*	1	0	0
GCF_000008165	*Bacillus anthracis str. Sterne*	1	0	0
GCF_000832635	*Bacillus anthracis str. Sterne*	0	0	0
GCF_000583105	*Bacillus anthracis str. SVA11*	3	1	0
GCF_000833275	*Bacillus anthracis str. Turkey32*	0	0	0
GCF_000832785	*Bacillus anthracis str. V770-NP-1R*	0	0	0

GCF_000742 895	*Bacillus anthracis str. Vollum*	0	0	0
GCF_019693 235	*Bacillus anthracis Strain: P04210076*	1	1	0
GCF_002688 645	*Bacillus atrophaeus*	10	6	9
GCF_002173 495	*Bacillus atrophaeus*	17	9	9
GCF_002243 495	*Bacillus atrophaeus*	17	9	11
GCF_018140 555	*Bacillus atrophaeus Strain: CNY01*	10	8	7
GCF_019443 725	*Bacillus atrophaeusStrain: PENSV20*	14	8	7
GCF_000165 925	*Bacillus atrophaeus 1942*	12	7	8
GCF_000742 675	*Bacillus atrophaeus subsp. globigii*	0	0	0
GCF_000385 965	*Bacillus atrophaeus UCMB-5137*	12	7	8
GCF_001721 685	*Bacillus beveridgei*	1	0	2
GCF_000831 065	*Bacillus bombysepticus str. Wang*	11	10	5
GCF_000835 185	*Bacillus cereus*	0	0	0
GCF_006384 875	*Bacillus cereus*	4	7	5
GCF_002290 105	*Bacillus cereus*	2	2	6
GCF_000978 375	*Bacillus cereus*	4	5	6
GCF_008041 975	*Bacillus cereus*	2	2	2
GCF_001277 915	*Bacillus cereus*	6	3	7
GCF_002813 875	*Bacillus cereus*	4	4	8
GCF_001518 875	*Bacillus cereus*	4	3	3
GCF_016027 015	*Bacillus cereus*	0	0	0
GCF_003013 315	*Bacillus cereus*	7	5	6

GCF_003020845	*Bacillus cereus*	5	4	0
GCF_009497015	*Bacillus cereus*	13	6	5
GCF_016727405	*Bacillus cereus*	7	5	1
GCF_016774535	*Bacillus cereus*	0	0	0
GCF_016774555	*Bacillus cereus*	10	7	1
GCF_016774575	*Bacillus cereus*	0	0	0
GCF_001635915	*Bacillus cereus*	13	9	6
GCF_001635955	*Bacillus cereus*	0	1	0
GCF_001635995	*Bacillus cereus*	7	5	8
GCF_016917795	*Bacillus cereus*	5	5	3
GCF_009739925	*Bacillus cereus*	4	3	4
GCF_009739965	*Bacillus cereus*	0	0	0
GCF_009739985	*Bacillus cereus*	4	3	2
GCF_001721145	*Bacillus cereus*	5	5	1
GCF_003568565	*Bacillus cereus*	9	8	5
GCF_001880305	*Bacillus cereus*	5	3	7
GCF_010223795	*Bacillus cereus*	4	6	3
GCF_010232525	*Bacillus cereus*	0	0	0
GCF_001941885	*Bacillus cereus*	2	1	2
GCF_001941905	*Bacillus cereus*	0	0	0
GCF_001941925	*Bacillus cereus*	0	0	0
GCF_000635895	*Bacillus cereus*	10	7	7

GCF_004006495	*Bacillus cereus*	5	3	3
GCF_002000005	*Bacillus cereus*	5	3	3
GCF_013112375	*Bacillus cereus*	1	0	0
GCF_002117465	*Bacillus cereus*	1	0	1
GCF_013177495	*Bacillus cereus*	7	4	6
GCF_000789315	*Bacillus cereus*	4	1	9
GCF_013267235	*Bacillus cereus*	0	0	0
GCF_013267255	*Bacillus cereus*	10	8	7
GCF_013267275	*Bacillus cereus*	0	0	0
GCF_013267455	*Bacillus cereus*	0	0	0
GCF_013267475	*Bacillus cereus*	0	0	0
GCF_013267775	*Bacillus cereus*	0	2	0
GCF_000832525	*Bacillus cereus*	3	2	3
GCF_002214705	*Bacillus cereus*	8	8	1
GCF_002214725	*Bacillus cereus*	1	2	5
GCF_002214765	*Bacillus cereus*	4	3	1
GCF_002215175	*Bacillus cereus*	7	4	1
GCF_002216125	*Bacillus cereus*	0	1	0
GCF_004771155	*Bacillus cereus*	3	5	2
GCF_000832765	*Bacillus cereus*	0	0	0
GCF_002220285	*Bacillus cereus*	6	3	5
GCF_004801195	*Bacillus cereus*	3	0	4

GCF_013394245	*Bacillus cereus*	2	1	0
GCF_005707595	*Bacillus cereus*	0	1	1
GCF_018309125	*Bacillus cereus Strain: BC06*	1	2	6
GCF_018309165	*Bacillus cereus Strain: BC33*	1	3	2
GCF_018884185	*Bacillus cereus Strain: CF4-51*	5	5	6
GCF_018309145	*Bacillus cereus Strain: BC07*	4	1	2
GCF_016117775	*Bacillus cereus Strain: FDAARGOS_919*	0	0	0
GCF_002952815	*Bacillus cereus Strain: G9241*	5	2	5
GCF_000022505	*Bacillus cereus 03BB102*	2	0	7
GCF_000832405	*Bacillus cereus 03BB102*	0	0	0
GCF_000832865	*Bacillus cereus 03BB108*	1	0	5
GCF_000160935	*Bacillus cereus 172560W*	11	8	7
GCF_000161135	*Bacillus cereus 95/8201*	2	1	8
GCF_000161375	*Bacillus cereus AH1271*	6	0	10
GCF_000161395	*Bacillus cereus AH1272*	1	0	1
GCF_000003955	*Bacillus cereus AH1273*	6	2	9
GCF_000021225	*Bacillus cereus AH187*	5	2	7
GCF_000161355	*Bacillus cereus AH676*	6	3	4
GCF_000021785	*Bacillus cereus AH820*	4	2	5
GCF_000160895	*Bacillus cereus ATCC 10876*	15	9	10
GCF_000008005	*Bacillus cereus ATCC 10987*	4	2	7
GCF_006094295	*Bacillus cereus ATCC 14579*	0	0	0

GCF_000007825	*Bacillus cereus ATCC 14579*	3	7	8
GCF_002504205	*Bacillus cereus ATCC 14579*	4	4	2
GCF_000161015	*Bacillus cereus ATCC 4342*	4	1	9
GCF_000832845	*Bacillus cereus ATCC 4342*	0	0	0
GCF_000021205	*Bacillus cereus B4264*	6	3	7
GCF_000161115	*Bacillus cereus BDRD-Cer4*	3	2	1
GCF_000161095	*Bacillus cereus BDRD-ST196*	9	4	9
GCF_000161055	*Bacillus cereus BDRD-ST24*	3	1	2
GCF_000160915	*Bacillus cereus BGSC 6E1*	6	6	3
GCF_000143605	*Bacillus cereus biovar anthracis str. CI*	7	2	4
GCF_002224345	*Bacillus cereus C1L*	6	7	6
GCF_000832385	*Bacillus cereus D17*	2	4	4
GCF_000011625	*Bacillus cereus E33L*	6	2	11
GCF_000833045	*Bacillus cereus E33L*	0	0	0
GCF_000338315	*Bacillus cereus F*	3	1	6
GCF_000161315	*Bacillus cereus F65185*	9	8	5
GCF_000239195	*Bacillus cereus F837/76*	1	1	3
GCF_000292415	*Bacillus cereus FRI-35*	3	3	4
GCF_000832805	*Bacillus cereus G9241*	0	0	0
GCF_000021305	*Bacillus cereus G9842*	8	4	9
GCF_000003645	*Bacillus cereus m1293*	4	3	7
GCF_000161035	*Bacillus cereus m1550*	5	4	4

GCF_000283 675	*Bacillus cereus NC7401*	0	0	0
GCF_000013 065	*Bacillus cereus Q1*	3	2	8
GCF_000161 175	*Bacillus cereus Rock1-15*	5	3	6
GCF_000161 155	*Bacillus cereus Rock1-3*	14	4	8
GCF_000161 195	*Bacillus cereus Rock3-28*	9	4	8
GCF_000161 215	*Bacillus cereus Rock3-29*	4	2	5
GCF_000161 235	*Bacillus cereus Rock3-42*	11	3	8
GCF_000161 255	*Bacillus cereus Rock3-44*	6	3	8
GCF_000161 295	*Bacillus cereus Rock4-18*	6	4	5
GCF_000161 275	*Bacillus cereus Rock4-2*	9	7	5
GCF_007923 085	*Bacillus cereus Strain: Co1-1*	1	0	1
GCF_002251 005	*Bacillus cytotoxicus*	1	0	0
GCF_002251 025	*Bacillus cytotoxicus*	0	0	0
GCF_002251 045	*Bacillus cytotoxicus*	0	0	0
GCF_002251 055	*Bacillus cytotoxicus*	0	0	0
GCF_002251 115	*Bacillus cytotoxicus*	0	0	0
GCF_002250 885	*Bacillus cytotoxicus*	5	7	2
GCF_002250 905	*Bacillus cytotoxicus*	3	3	2
GCF_002250 925	*Bacillus cytotoxicus*	1	0	2
GCF_002250 945	*Bacillus cytotoxicus*	0	1	0
GCF_002250 965	*Bacillus cytotoxicus*	0	1	0
GCF_017909 295	*Bacillus cytotoxicus Strain: E28.3*	0	1	0

GCF_017909 135	*Bacillus cytotoxicus Strain: SM1.1*	0	0	0
GCF_017909 395	*Bacillus cytotoxicus Strain: E17.4*	0	0	0
GCF_017909 435	*Bacillus cytotoxicus Strain: E8.1*	0	1	0
GCF_017909 235	*Bacillus cytotoxicus Strain: PDT2.12*	0	2	0
GCF_017909 015	*Bacillus cytotoxicus Strain: SM2.8*	2	3	3
GCF_000017 425	*Bacillus cytotoxicus NVH 391-98*	5	5	6
GCF_007995 155	*Bacillus dafuensis*	4	3	9
GCF_900636 945	*Bacillus freudenreichii*	3	0	5
GCF_002443 095	*Bacillus glycinifermentans*	9	6	7
GCF_004103 615	*Bacillus glycinifermentans*	8	2	7
GCF_900093 775	*Bacillus glycinifermentans*	0	0	0
GCF_001278 705	*Bacillus gobiensis*	2	4	5
GCF_018623 075	*Bacillus haikouensis Strain: MNJ12*	3	0	6
GCF_014621 755	*Bacillus halotolerans*	9	3	5
GCF_006965 425	*Bacillus halotolerans*	9	4	7
GCF_016093 395	*Bacillus halotolerans*	12	5	7
GCF_004006 435	*Bacillus halotolerans*	11	5	8
GCF_018417 515	*Bacillus halotolerans Strain: MBH1*	11	4	8
GCF_014042 035	*Bacillus haynesii*	13	3	7
GCF_001534 785	*Bacillus inaquosorum*	16	10	10
GCF_003148 415	*Bacillus inaquosorum*	14	11	9
GCF_019598 985	*Bacillus inaquosorum Strain: CCSR02*	7	5	2

GCF_000473245	*Bacillus infantis NRRL B-14911*	1	0	11
GCF_000959025	*Bacillus intestinalis*	1	0	2
GCF_006094335	*Bacillus licheniformis*	0	1	0
GCF_006494795	*Bacillus licheniformis*	1	1	0
GCF_014673515	*Bacillus licheniformis*	0	0	0
GCF_006974025	*Bacillus licheniformis*	3	0	1
GCF_002504325	*Bacillus licheniformis*	1	2	2
GCF_007855455	*Bacillus licheniformis*	0	0	1
GCF_016026835	*Bacillus licheniformis*	1	1	1
GCF_002966955	*Bacillus licheniformis*	1	1	1
GCF_016126875	*Bacillus licheniformis*	0	0	0
GCF_001596055	*Bacillus licheniformis*	3	1	3
GCF_009662395	*Bacillus licheniformis*	0	0	0
GCF_003253815	*Bacillus licheniformis*	1	1	0
GCF_003339545	*Bacillus licheniformis*	0	0	0
GCF_001726125	*Bacillus licheniformis*	3	2	1
GCF_003595625	*Bacillus licheniformis*	2	1	1
GCF_003606405	*Bacillus licheniformis*	1	2	0
GCF_001914255	*Bacillus licheniformis*	2	1	1
GCF_003952865	*Bacillus licheniformis*	0	0	0
GCF_004101525	*Bacillus licheniformis*	1	0	0
GCF_002074075	*Bacillus licheniformis*	2	1	3

GCF_002074095	*Bacillus licheniformis*	1	1	0
GCF_002074115	*Bacillus licheniformis*	3	2	1
GCF_002074135	*Bacillus licheniformis*	2	0	0
GCF_004103575	*Bacillus licheniformis*	1	0	1
GCF_002173675	*Bacillus licheniformis*	2	0	0
GCF_004119755	*Bacillus licheniformis*	0	1	0
GCF_002174255	*Bacillus licheniformis*	0	0	0
GCF_004119795	*Bacillus licheniformis*	0	1	0
GCF_004526275	*Bacillus licheniformis*	2	1	0
GCF_002236895	*Bacillus licheniformis*	3	1	1
GCF_902386215	*Bacillus licheniformis*	0	0	0
GCF_017338795	*Bacillus licheniformis Strain: CP6*	4	1	0
GCF_015689075	*Bacillus licheniformis Strain: LCDD6*	2	1	0
GCF_002953475	*Bacillus licheniformis Strain: MBGJa67*	2	1	1
GCF_000008425	*Bacillus licheniformis DSM 13 = ATCC 14580*	6	2	7
GCF_000011645	*Bacillus licheniformis DSM 13 = ATCC 14580*	0	0	0
GCF_000258125	*Bacillus licheniformis WX-02*	4	2	2
GCF_009739945	*Bacillus luti*	7	2	7
GCF_000724485	*Bacillus methanolicus MGA3*	1	2	7
GCF_003612955	*Bacillus mobilis*	5	0	7
GCF_015074845	*Bacillus mojavensis*	9	6	9
GCF_012648005	*Bacillus mojavensis*	9	4	6

GCF_008274925	*Bacillus mycoides*	9	3	12
GCF_000160975	*Bacillus mycoides*	13	6	10
GCF_000161335	*Bacillus mycoides*	7	3	10
GCF_002104575	*Bacillus mycoides*	7	4	8
GCF_000775975	*Bacillus mycoides*	1	0	0
GCF_000832605	*Bacillus mycoides*	0	0	0
GCF_018736725	*Bacillus mycoides Strain: BPN07/3*	3	2	10
GCF_018736765	*Bacillus mycoides Strain: BPN102*	4	3	5
GCF_018739385	*Bacillus mycoides Strain: BPN29/1*	7	1	7
GCF_018742205	*Bacillus mycoides Strain: BPN29/1*	0	0	0
GCF_018739485	*Bacillus mycoides Strain: BPN36/3*	3	3	0
GCF_018739525	*Bacillus mycoides Strain: BPN37/2*	10	2	7
GCF_018739625	*Bacillus mycoides Strain: BPN51/1*	6	1	5
GCF_018739645	*Bacillus mycoides Strain: BPN52/2*	6	2	5
GCF_018741085	*Bacillus mycoides Strain: BPN601*	0	0	0
GCF_018741145	*Bacillus mycoides Strain: JAS06/1*	5	2	4
GCF_018741165	*Bacillus mycoides Strain: JAS06/3*	6	4	6
GCF_018742045	*Bacillus mycoides Strain: JAS12/5*	0	0	0
GCF_018741565	*Bacillus mycoides Strain: JAS23/1*	5	1	6
GCF_018741605	*Bacillus mycoides Strain: JAS391*	10	3	7
GCF_018741645	*Bacillus mycoides Strain: JAS481*	9	2	2
GCF_018736705	*Bacillus mycoides Strain: BPN03/1*	5	1	11

GCF_018736 745	*Bacillus mycoides Strain: BPN09/1*	7	1	4
GCF_018739 305	*Bacillus mycoides Strain: BPN121*	6	5	7
GCF_018739 505	*Bacillus mycoides Strain: BPN37/1*	5	1	6
GCF_018739 545	*Bacillus mycoides Strain: BPN43/2*	0	1	0
GCF_018739 665	*Bacillus mycoides Strain: BPN54/2*	1	0	3
GCF_018740 945	*Bacillus mycoides Strain: BPN573*	0	0	0
GCF_018741 005	*Bacillus mycoides Strain: BPN58/4*	5	1	5
GCF_018739 705	*Bacillus mycoides Strain: JAS014*	2	2	7
GCF_018742 185	*Bacillus mycoides Strain: JAS06/1*	0	0	0
GCF_018741 465	*Bacillus mycoides Strain: JAS1004*	10	1	6
GCF_018741 505	*Bacillus mycoides Strain: JAS12/5*	4	1	9
GCF_018751 995	*Bacillus mycoides Strain: JAS15/1*	4	1	0
GCF_018742 225	*Bacillus mycoides Strain: JAS635*	0	1	0
GCF_018742 105	*Bacillus mycoides Strain: JAS85/1*	3	1	1
GCF_018742 265	*Bacillus mycoides Strain: JAS85/1*	0	0	0
GCF_018742 285	*Bacillus mycoides Strain: JAS94/5*	5	1	0
GCF_000003 925	*Bacillus mycoides DSM 2048*	8	2	9
GCF_000018 825	*Bacillus mycoides KBAB4*	10	3	9
GCF_018739 365	*Bacillus mycoides Strain: BPN211*	5	4	5
GCF_018739 445	*Bacillus mycoides Strain: BPN36/2*	7	1	5
GCF_018739 685	*Bacillus mycoides Strain: BPN57/2*	5	4	7
GCF_016117 755	*Bacillus mycoides Strain: FDAARGOS_924*	0	0	0

GCF_018742245	*Bacillus mycoides Strain: JAS823*	1	1	3
GCF_018141025	*Bacillus nitratireducens Strain: BM02*	3	3	9
GCF_016925055	*Bacillus pacificus Strain: Colony7*	4	1	5
GCF_016925035	*Bacillus pacificus Strain: Colony 6*	1	2	3
GCF_016925015	*Bacillus pacificus Strain: Colony501*	2	2	6
GCF_000876525	*Bacillus paralicheniformis*	10	3	6
GCF_002393225	*Bacillus paralicheniformis*	10	5	6
GCF_008365255	*Bacillus paralicheniformis*	5	0	1
GCF_002993925	*Bacillus paralicheniformis*	10	3	6
GCF_002993945	*Bacillus paralicheniformis*	15	6	5
GCF_009497935	*Bacillus paralicheniformis*	1	1	0
GCF_003711025	*Bacillus paralicheniformis*	3	1	0
GCF_011068305	*Bacillus paralicheniformis*	11	2	7
GCF_002068155	*Bacillus paralicheniformis*	11	2	2
GCF_900635765	*Bacillus paralicheniformis*	8	1	1
GCF_018324565	*Bacillus paralicheniformis Strain: RSC-2*	2	1	0
GCF_000408885	*Bacillus paralicheniformis ATCC 9945a*	13	3	7
GCF_018324505	*Bacillus paralicheniformis Strain: RSC-1*	9	2	2
GCF_015334865	*Bacillus paranthracis*	1	0	0
GCF_015693885	*Bacillus paranthracis*	2	2	2
GCF_009648955	*Bacillus paranthracis*	0	0	0
GCF_016834515	*Bacillus paranthracis*	0	0	0

GCF_009799965	*Bacillus paranthracis*	1	0	2
GCF_010669045	*Bacillus paranthracis*	2	1	0
GCF_005848985	*Bacillus paranthracis*	4	4	8
GCF_018513785	*Bacillus paranthracis Strain: Colony502*	2	2	3
GCF_000161415	*Bacillus pseudomycoides*	16	7	10
GCF_000161435	*Bacillus pseudomycoides*	20	6	8
GCF_000742855	*Bacillus pseudomycoides*	5	4	8
GCF_000832885	*Bacillus pseudomycoides*	1	1	0
GCF_000161455	*Bacillus pseudomycoides DSM 12442*	19	14	13
GCF_014489355	*Bacillus pumilus*	10	8	5
GCF_001548215	*Bacillus pumilus*	4	4	4
GCF_001578165	*Bacillus pumilus*	8	7	8
GCF_001578205	*Bacillus pumilus*	9	8	6
GCF_001687085	*Bacillus pumilus*	2	4	5
GCF_003431975	*Bacillus pumilus*	9	9	8
GCF_001704975	*Bacillus pumilus*	9	7	7
GCF_003571425	*Bacillus pumilus*	8	6	5
GCF_009937765	*Bacillus pumilus*	10	12	2
GCF_000590455	*Bacillus pumilus*	4	4	8
GCF_004006455	*Bacillus pumilus*	7	7	4
GCF_013423765	*Bacillus pumilus*	9	8	6
GCF_900186955	*Bacillus pumilus*	10	7	6

GCF_000017 885	*Bacillus pumilus SAFR-032*	8	8	5
GCF_008244 765	*Bacillus safensis*	10	7	5
GCF_016803 835	*Bacillus safensis*	6	7	7
GCF_001895 885	*Bacillus safensis*	11	7	7
GCF_001938 665	*Bacillus safensis*	4	4	6
GCF_001938 685	*Bacillus safensis*	7	4	6
GCF_001938 705	*Bacillus safensis*	0	0	0
GCF_002077 215	*Bacillus safensis*	7	6	3
GCF_003660 145	*Bacillus safensis Strain: sami*	11	7	7
GCF_003097 715	*Bacillus safensis FO-36b*	6	5	5
GCF_002850 535	*Bacillus siamensis*	1	1	2
GCF_016313 165	*Bacillus siamensis*	11	9	10
GCF_001050 115	*Bacillus smithii*	3	1	6
GCF_002202 015	*Bacillus sonorensis*	12	6	7
GCF_000242 895	*Bacillus sp. 1NLA3E*	3	0	8
GCF_002269 275	*Bacillus sp. 1s-1*	0	0	0
GCF_001999 205	*Bacillus sp. 275*	11	10	6
GCF_017161 885	*Bacillus sp. 3a*	9	8	4
GCF_019464 555	*Bacillus sp. 7D3*	3	1	1
GCF_009861 435	*Bacillus sp. A260*	1	1	1
GCF_001831 375	*Bacillus sp. ABP14*	4	1	6
GCF_009906 915	*Bacillus sp. AM1(2019)*	13	13	8

GCF_008274865	*Bacillus sp. AR4-2*	7	3	0
GCF_001679765	*Bacillus sp. B25*	5	3	5
GCF_007363175	*Bacillus sp. BD59S*	6	2	7
GCF_000827045	*Bacillus sp. BH072*	3	3	0
GCF_008432245	*Bacillus sp. BS98*	5	3	1
GCF_003355115	*Bacillus sp. COPE52*	10	6	7
GCF_003429245	*Bacillus sp. CR71*	2	0	0
GCF_012955605	*Bacillus sp. CS13*	1	1	6
GCF_011082085	*Bacillus sp. Cs-700*	2	0	9
GCF_003330965	*Bacillus sp. DM2*	5	1	1
GCF_009720625	*Bacillus sp. DSL-17*	4	0	7
GCF_004799705	*Bacillus sp. DU-106*	3	0	4
GCF_003429225	*Bacillus sp. E25*	10	7	4
GCF_002073415	*Bacillus sp. FDAARGOS_235*	4	1	0
GCF_003812125	*Bacillus sp. FDAARGOS_527*	1	0	1
GCF_001719225	*Bacillus sp. FJAT-14266*	9	3	5
GCF_001278805	*Bacillus sp. FJAT-18017*	1	0	7
GCF_001278755	*Bacillus sp. FJAT-22090*	3	2	6
GCF_003816055	*Bacillus sp. FJAT-42376*	3	0	7
GCF_002813025	*Bacillus sp. FJAT-45348*	1	1	7
GCF_001896025	*Bacillus sp. H15-1*	1	2	1
GCF_002833485	*Bacillus sp. HBCD-sjtu*	4	1	4

GCF_014960805	*Bacillus sp. HD4P25*	4	1	5
GCF_012572025	*Bacillus sp. HNA3*	1	1	1
GCF_001648135	*Bacillus sp. IHB B 7164*	2	4	5
GCF_008274905	*Bacillus sp. JAS24-2*	9	5	5
GCF_018861275	*Bacillus sp. JNUCC-22*	9	12	10
GCF_018885385	*Bacillus sp. JNUCC-24*	7	6	4
GCF_000259365	*Bacillus sp. JS*	12	7	12
GCF_005937795	*Bacillus sp. KBS0812*	0	0	0
GCF_011398925	*Bacillus sp. KH172YL63*	2	1	8
GCF_016924995	*Bacillus sp. LJBS06*	11	8	8
GCF_019334685	*Bacillus sp. LJBS17*	5	3	3
GCF_017797845	*Bacillus sp. LJBV19*	12	13	10
GCF_000978495	*Bacillus sp. LM 4-2*	8	4	5
GCF_011008975	*Bacillus sp. LUNF1*	0	0	0
GCF_002763675	*Bacillus sp. Lzh-5*	2	1	0
GCF_006965545	*Bacillus sp. M4U3P1*	1	0	3
GCF_002953915	*Bacillus sp. MBGLi79*	16	6	15
GCF_002201875	*Bacillus sp. MD-5*	11	7	10
GCF_009759805	*Bacillus sp. ms-22*	4	4	6
GCF_009818105	*Bacillus sp. N1-1*	3	0	9
GCF_009676825	*Bacillus sp. N3536*	3	1	7
GCF_018889975	*Bacillus sp. NP157*	3	3	1

GCF_000465465	*Bacillus sp. NSP9.1*	6	2	8
GCF_000829195	*Bacillus sp. OxB-1*	4	2	11
GCF_014302175	*Bacillus sp. PAMC22265*	0	0	0
GCF_014250375	*Bacillus sp. PAMC26543*	10	7	5
GCF_014235785	*Bacillus sp. PAMC26568*	4	0	7
GCF_014250435	*Bacillus sp. PAMC28571*	9	7	5
GCF_014250415	*Bacillus sp. PAMC28748*	1	0	0
GCF_000815145	*Bacillus sp. Pc3*	13	14	12
GCF_016919285	*Bacillus sp. PDNC022*	7	4	5
GCF_001816185	*Bacillus sp. RZ2MS9*	6	3	4
GCF_005154805	*Bacillus sp. S3*	3	0	10
GCF_000469135	*Bacillus sp. SB49*	2	1	7
GCF_001553895	*Bacillus sp. SDLI1*	12	13	13
GCF_008274885	*Bacillus sp. SH8-8*	0	0	0
GCF_002843505	*Bacillus sp. SJ-10*	7	5	5
GCF_003171815	*Bacillus sp. SUBG0010*	11	2	5
GCF_004322655	*Bacillus sp. SYJ*	4	1	10
GCF_009363695	*Bacillus sp. THAF10*	3	1	5
GCF_016653575	*Bacillus sp. TK-2*	6	4	6
GCF_015999585	*Bacillus sp. V3*	4	0	7
GCF_000800825	*Bacillus sp. WP8*	8	4	5
GCF_004103875	*Bacillus sp. WR11*	0	0	0

GCF_000747 345	*Bacillus sp. X1(2014)*	4	0	7
GCF_003047 225	*Bacillus sp. Y-01*	2	4	3
GCF_003586 445	*Bacillus sp. Y1*	3	0	5
GCF_000877 815	*Bacillus sp. YP1*	9	5	6
GCF_003047 165	*Bacillus sp. ZY-1-1*	7	14	8
GCF_002269 175	*Bacillus subtilis*	3	3	2
GCF_002269 195	*Bacillus subtilis*	0	0	1
GCF_006165 085	*Bacillus subtilis*	2	3	2
GCF_006364 495	*Bacillus subtilis*	0	0	0
GCF_000952 895	*Bacillus subtilis*	0	0	0
GCF_000953 615	*Bacillus subtilis*	0	0	0
GCF_002290 305	*Bacillus subtilis*	14	11	4
GCF_014879 275	*Bacillus subtilis*	7	5	8
GCF_001015 095	*Bacillus subtilis*	9	4	8
GCF_001037 985	*Bacillus subtilis*	0	0	0
GCF_015535 655	*Bacillus subtilis*	0	2	1
GCF_015602 625	*Bacillus subtilis*	5	4	2
GCF_015654 205	*Bacillus subtilis*	1	0	0
GCF_002893 805	*Bacillus subtilis*	7	5	5
GCF_008831 405	*Bacillus subtilis*	1	1	0
GCF_016065 415	*Bacillus subtilis*	0	0	0
GCF_009017 455	*Bacillus subtilis*	9	6	7

GCF_002982175	*Bacillus subtilis*	0	0	0
GCF_009363835	*Bacillus subtilis*	5	1	4
GCF_009497795	*Bacillus subtilis*	10	4	7
GCF_009497815	*Bacillus subtilis*	8	4	8
GCF_009662155	*Bacillus subtilis*	4	2	1
GCF_009662175	*Bacillus subtilis*	1	2	0
GCF_009662195	*Bacillus subtilis*	4	2	2
GCF_009662215	*Bacillus subtilis*	1	0	2
GCF_009662235	*Bacillus subtilis*	5	2	2
GCF_001604995	*Bacillus subtilis*	0	0	1
GCF_009662255	*Bacillus subtilis*	1	0	1
GCF_009662275	*Bacillus subtilis*	1	0	0
GCF_009662335	*Bacillus subtilis*	3	2	3
GCF_003148355	*Bacillus subtilis*	8	3	6
GCF_009662355	*Bacillus subtilis*	4	2	1
GCF_009662375	*Bacillus subtilis*	2	1	1
GCF_009662415	*Bacillus subtilis*	0	0	0
GCF_009662435	*Bacillus subtilis*	0	0	0
GCF_009662455	*Bacillus subtilis*	0	0	0
GCF_016894165	*Bacillus subtilis*	8	5	5
GCF_003184225	*Bacillus subtilis*	5	4	4
GCF_009665145	*Bacillus subtilis*	0	0	0

GCF_009676665	*Bacillus subtilis*	3	5	0
GCF_001704095	*Bacillus subtilis*	4	2	1
GCF_001720505	*Bacillus subtilis*	8	5	5
GCF_009857115	*Bacillus subtilis*	1	1	0
GCF_009866865	*Bacillus subtilis*	7	2	2
GCF_009884005	*Bacillus subtilis*	3	1	0
GCF_001746575	*Bacillus subtilis*	5	3	2
GCF_009913255	*Bacillus subtilis*	10	5	4
GCF_001747445	*Bacillus subtilis*	5	3	4
GCF_009913275	*Bacillus subtilis*	9	5	7
GCF_003610955	*Bacillus subtilis*	0	0	1
GCF_001808235	*Bacillus subtilis*	10	4	8
GCF_009913395	*Bacillus subtilis*	4	2	1
GCF_009913515	*Bacillus subtilis*	4	4	4
GCF_009913535	*Bacillus subtilis*	0	0	0
GCF_009913555	*Bacillus subtilis*	3	0	2
GCF_009913575	*Bacillus subtilis*	9	5	6
GCF_009913595	*Bacillus subtilis*	9	6	7
GCF_001889385	*Bacillus subtilis*	10	13	10
GCF_001889625	*Bacillus subtilis*	8	5	6
GCF_009914155	*Bacillus subtilis*	3	2	2
GCF_001890405	*Bacillus subtilis*	0	0	0

GCF_009937745	*Bacillus subtilis*	21	8	7
GCF_001902555	*Bacillus subtilis*	0	0	0
GCF_011765645	*Bacillus subtilis*	6	3	2
GCF_004101345	*Bacillus subtilis*	6	3	1
GCF_004101365	*Bacillus subtilis*	1	0	0
GCF_004101405	*Bacillus subtilis*	0	0	0
GCF_004101425	*Bacillus subtilis*	3	2	2
GCF_004101445	*Bacillus subtilis*	3	0	0
GCF_002055965	*Bacillus subtilis*	0	0	0
GCF_004101465	*Bacillus subtilis*	1	0	1
GCF_004101485	*Bacillus subtilis*	4	3	4
GCF_004101565	*Bacillus subtilis*	0	0	0
GCF_002072735	*Bacillus subtilis*	10	5	5
GCF_004101945	*Bacillus subtilis*	6	1	5
GCF_004103535	*Bacillus subtilis*	11	4	4
GCF_012647565	*Bacillus subtilis*	14	8	7
GCF_004103555	*Bacillus subtilis*	1	1	1
GCF_004103595	*Bacillus subtilis*	10	5	6
GCF_002096095	*Bacillus subtilis*	2	0	0
GCF_000769515	*Bacillus subtilis*	17	23	11
GCF_004119535	*Bacillus subtilis*	11	5	6
GCF_000772125	*Bacillus subtilis*	1	1	0

GCF_004119555	*Bacillus subtilis*	1	1	1
GCF_000772165	*Bacillus subtilis*	9	11	7
GCF_000772205	*Bacillus subtilis*	5	6	1
GCF_004119595	*Bacillus subtilis*	0	0	0
GCF_002142595	*Bacillus subtilis*	10	5	6
GCF_004119615	*Bacillus subtilis*	3	1	5
GCF_000782835	*Bacillus subtilis*	11	5	7
GCF_002163815	*Bacillus subtilis*	14	9	10
GCF_004119635	*Bacillus subtilis*	4	2	2
GCF_004119655	*Bacillus subtilis*	10	4	1
GCF_000789295	*Bacillus subtilis*	0	0	0
GCF_004119675	*Bacillus subtilis*	3	1	1
GCF_004119695	*Bacillus subtilis*	10	4	7
GCF_004119715	*Bacillus subtilis*	1	0	0
GCF_004119775	*Bacillus subtilis*	6	3	4
GCF_004119815	*Bacillus subtilis*	9	5	4
GCF_004119835	*Bacillus subtilis*	6	1	2
GCF_004119875	*Bacillus subtilis*	1	0	0
GCF_013285325	*Bacillus subtilis*	10	5	6
GCF_002202055	*Bacillus subtilis*	4	1	0
GCF_002205435	*Bacillus subtilis*	9	3	8
GCF_013347105	*Bacillus subtilis*	11	2	5

GCF_002216085	*Bacillus subtilis*	0	0	0
GCF_013388335	*Bacillus subtilis*	0	0	0
GCF_002220075	*Bacillus subtilis*	6	4	3
GCF_013393725	*Bacillus subtilis*	5	5	2
GCF_005153965	*Bacillus subtilis*	0	0	0
GCF_005160425	*Bacillus subtilis*	2	0	0
GCF_005234095	*Bacillus subtilis*	7	2	6
GCF_005849145	*Bacillus subtilis*	0	0	2
GCF_014171535	*Bacillus subtilis*	8	4	3
GCF_018394175	*Bacillus subtilis Strain: BYS2*	0	0	2
GCF_018141065	*Bacillus subtilis Strain: PMB102*	7	5	7
GCF_018224865	*Bacillus subtilis Strain: SC3.7*	10	8	7
GCF_003515125	*Bacillus subtilis Strain: FB6-3*	6	2	5
GCF_019464335	*Bacillus subtilis Strain: HD15*	7	2	2
GCF_018884165	*Bacillus subtilis Strain: s-16*	4	1	2
GCF_017753785	*Bacillus subtilis Strain: YB-04*	0	1	0
GCF_000523045	*Bacillus subtilis BEST7003*	2	1	0
GCF_000186745	*Bacillus subtilis BSn5*	10	7	8
GCF_000973605	*Bacillus subtilis HJ5*	4	2	2
GCF_000971925	*Bacillus subtilis KCTC 1028 = ATCC 6051a*	0	0	0
GCF_000497485	*Bacillus subtilis PY79*	1	0	0
GCF_000293765	*Bacillus subtilis QB928*	2	1	0

GCF_019599105	*Bacillus subtilis Strain: YPS-32*	0	0	0
GCF_001597265	*Bacillus subtilis subsp. globigii*	1	1	0
GCF_001565875	*Bacillus subtilis subsp. natto*	1	1	0
GCF_000209795	*Bacillus subtilis subsp. natto BEST195*	6	3	6
GCF_006094475	*Bacillus subtilis subsp. spizizenii ATCC 6633*	0	0	0
GCF_000146565	*Bacillus subtilis subsp. spizizenii str. W23*	8	6	8
GCF_000227465	*Bacillus subtilis subsp. spizizenii TU-B-10*	7	6	9
GCF_014298115	*Bacillus subtilis subsp. subtilis*	10	5	7
GCF_006741845	*Bacillus subtilis subsp. subtilis*	0	1	0
GCF_014961985	*Bacillus subtilis subsp. subtilis*	1	2	2
GCF_001465815	*Bacillus subtilis subsp. subtilis*	2	1	0
GCF_001541905	*Bacillus subtilis subsp. subtilis*	0	0	0
GCF_001660525	*Bacillus subtilis subsp. subtilis*	0	0	0
GCF_003426125	*Bacillus subtilis subsp. subtilis*	10	6	8
GCF_001697265	*Bacillus subtilis subsp. subtilis*	0	1	2
GCF_001703495	*Bacillus subtilis subsp. subtilis*	0	0	0
GCF_001750745	*Bacillus subtilis subsp. subtilis*	0	1	0
GCF_001750765	*Bacillus subtilis subsp. subtilis*	0	2	0
GCF_003665195	*Bacillus subtilis subsp. subtilis*	0	0	0
GCF_003665215	*Bacillus subtilis subsp. subtilis*	5	4	7
GCF_003665235	*Bacillus subtilis subsp. subtilis*	3	2	2
GCF_003665255	*Bacillus subtilis subsp. subtilis*	6	0	3

GCF_003665 275	*Bacillus subtilis subsp. subtilis*	2	0	0
GCF_003665 295	*Bacillus subtilis subsp. subtilis*	0	0	0
GCF_003665 315	*Bacillus subtilis subsp. subtilis*	0	1	0
GCF_003665 335	*Bacillus subtilis subsp. subtilis*	0	0	0
GCF_003665 355	*Bacillus subtilis subsp. subtilis*	0	0	0
GCF_003665 395	*Bacillus subtilis subsp. subtilis*	0	0	0
GCF_012648 205	*Bacillus subtilis subsp. subtilis*	2	1	1
GCF_012648 345	*Bacillus subtilis subsp. subtilis*	0	0	0
GCF_002173 615	*Bacillus subtilis subsp. subtilis*	1	0	0
GCF_002173 695	*Bacillus subtilis subsp. subtilis*	4	2	3
GCF_002173 715	*Bacillus subtilis subsp. subtilis*	0	0	0
GCF_000827 065	*Bacillus subtilis subsp. subtilis*	0	0	0
GCF_002201 955	*Bacillus subtilis subsp. subtilis*	0	0	0
GCF_002201 995	*Bacillus subtilis subsp. subtilis*	3	0	3
GCF_002202 035	*Bacillus subtilis subsp. subtilis*	12	6	4
GCF_004328 925	*Bacillus subtilis subsp. subtilis*	12	3	5
GCF_018454 405	*Bacillus subtilis subsp. subtilis Strain: A1 - Midalam*	4	2	2
GCF_000344 745	*Bacillus subtilis subsp. subtilis 6051-HGW*	0	0	0
GCF_006088 795	*Bacillus subtilis subsp. subtilis NCIB 3610 = ATCC 6051*	0	0	0
GCF_000186 085	*Bacillus subtilis subsp. subtilis NCIB 3610 = ATCC 6051*	2	1	0
GCF_000009 045	*Bacillus subtilis subsp. subtilis str. 168*	11	5	8
GCF_000155 325	*Bacillus subtilis subsp. subtilis str. 168*	3	1	2

GCF_002009095	*Bacillus subtilis subsp. subtilis str. 168*	0	0	0
GCF_002009135	*Bacillus subtilis subsp. subtilis str. 168*	0	0	0
GCF_012931705	*Bacillus subtilis subsp. subtilis str. 168*	0	0	0
GCF_013009385	*Bacillus subtilis subsp. subtilis str. 168*	1	0	0
GCF_000789275	*Bacillus subtilis subsp. subtilis str. 168*	0	0	0
GCF_000699525	*Bacillus subtilis subsp. subtilis str. AG1839*	0	0	0
GCF_000349795	*Bacillus subtilis subsp. subtilis str. BAB-1*	7	3	6
GCF_000321395	*Bacillus subtilis subsp. subtilis str. BSP1*	10	5	8
GCF_000155355	*Bacillus subtilis subsp. subtilis str. JH642*	2	2	0
GCF_000699465	*Bacillus subtilis subsp. subtilis str. JH642 substr. AG174*	0	0	0
GCF_000706705	*Bacillus subtilis subsp. subtilis str. OH 131.1*	9	5	8
GCF_000227485	*Bacillus subtilis subsp. subtilis str. RO-NN-1*	10	5	9
GCF_000155375	*Bacillus subtilis subsp. subtilis str. SMY*	1	1	1
GCF_012030675	*Bacillus subtilis subsp. subtilis str. SMY*	0	0	0
GCF_018986855	*Bacillus subtilis subsp. subtilisStrain: Miz-8*	1	2	0
GCF_000737405	*Bacillus subtilis TO-A*	6	4	3
GCF_000338735	*Bacillus subtilis XF-1*	10	5	9
GCF_012225885	*Bacillus tequilensis*	10	6	6
GCF_006151925	*Bacillus thuringiensis*	12	5	4
GCF_001017635	*Bacillus thuringiensis*	0	0	0
GCF_001182785	*Bacillus thuringiensis*	7	4	6
GCF_001420855	*Bacillus thuringiensis*	0	0	0

GCF_001455 345	*Bacillus thuringiensis*	7	2	11
GCF_009025 915	*Bacillus thuringiensis*	0	0	0
GCF_016070 565	*Bacillus thuringiensis*	8	3	4
GCF_009224 065	*Bacillus thuringiensis*	5	4	3
GCF_001595 725	*Bacillus thuringiensis*	20	10	3
GCF_003054 785	*Bacillus thuringiensis*	10	8	3
GCF_001598 095	*Bacillus thuringiensis*	4	0	2
GCF_001618 665	*Bacillus thuringiensis*	0	0	0
GCF_001685 565	*Bacillus thuringiensis*	2	1	5
GCF_001692 675	*Bacillus thuringiensis*	5	6	3
GCF_003626 955	*Bacillus thuringiensis*	4	1	1
GCF_003991 175	*Bacillus thuringiensis*	6	3	3
GCF_002025 105	*Bacillus thuringiensis*	5	4	3
GCF_002119 445	*Bacillus thuringiensis*	1	0	0
GCF_013267 295	*Bacillus thuringiensis*	0	0	0
GCF_002173 755	*Bacillus thuringiensis*	2	3	3
GCF_013267 315	*Bacillus thuringiensis*	0	0	0
GCF_013267 335	*Bacillus thuringiensis*	0	0	0
GCF_002184 245	*Bacillus thuringiensis*	6	3	4
GCF_002192 515	*Bacillus thuringiensis*	4	1	2
GCF_013267 795	*Bacillus thuringiensis*	0	0	0
GCF_013267 815	*Bacillus thuringiensis*	0	0	0

GCF_004194435	*Bacillus thuringiensis*	3	0	1
GCF_004194455	*Bacillus thuringiensis*	1	0	0
GCF_004194475	*Bacillus thuringiensis*	2	1	1
GCF_000832485	*Bacillus thuringiensis*	0	2	0
GCF_013340745	*Bacillus thuringiensis*	11	9	6
GCF_013343075	*Bacillus thuringiensis*	0	0	0
GCF_004354265	*Bacillus thuringiensis*	6	0	1
GCF_000832825	*Bacillus thuringiensis*	0	0	0
GCF_002222555	*Bacillus thuringiensis*	4	3	3
GCF_005155285	*Bacillus thuringiensis*	0	0	0
GCF_000832925	*Bacillus thuringiensis*	5	1	4
GCF_000833085	*Bacillus thuringiensis*	5	0	4
GCF_017751245	*Bacillus thuringiensis Strain: Bt-GS57*	0	0	0
GCF_017378115	*Bacillus thuringiensis Strain: TAND672*	0	0	0
GCF_000092165	*Bacillus thuringiensis BMB171*	7	6	5
GCF_001721165	*Bacillus thuringiensis Bt18247*	3	2	4
GCF_000161495	*Bacillus thuringiensis Bt407*	7	5	8
GCF_000306745	*Bacillus thuringiensis Bt407*	0	0	0
GCF_000342025	*Bacillus thuringiensis DAR 81934*	8	5	7
GCF_000835025	*Bacillus thuringiensis HD1002*	0	0	0
GCF_000292455	*Bacillus thuringiensis HD-771*	7	5	6
GCF_000292705	*Bacillus thuringiensis HD-789*	2	0	0

GCF_000161715	*Bacillus thuringiensis IBL 200*	10	7	9
GCF_000161735	*Bacillus thuringiensis IBL 4222*	13	6	8
GCF_003546665	*Bacillus thuringiensis LM1212*	6	2	9
GCF_000300475	*Bacillus thuringiensis MC28*	2	2	5
GCF_001640965	*Bacillus thuringiensis serovar alesti*	6	3	6
GCF_012222085	*Bacillus thuringiensis serovar andalousiensis*	6	5	10
GCF_000161635	*Bacillus thuringiensis serovar andalousiensis BGSC 4AW1*	4	3	4
GCF_000161615	*Bacillus thuringiensis serovar berliner ATCC 10792*	2	2	1
GCF_000193355	*Bacillus thuringiensis serovar chinensis CT-43*	0	0	0
GCF_000803665	*Bacillus thuringiensis serovar galleriae*	6	6	1
GCF_000161675	*Bacillus thuringiensis serovar huazhongensis BGSC 4BD1*	22	8	7
GCF_001183785	*Bacillus thuringiensis serovar indiana*	10	5	4
GCF_003445395	*Bacillus thuringiensis serovar israelensis*	0	0	1
GCF_013113775	*Bacillus thuringiensis serovar israelensis*	0	0	0
GCF_000835235	*Bacillus thuringiensis serovar kurstaki*	0	0	0
GCF_000717535	*Bacillus thuringiensis serovar kurstaki str. HD-1*	7	2	3
GCF_000338755	*Bacillus thuringiensis serovar kurstaki str. HD73*	1	0	0
GCF_000161575	*Bacillus thuringiensis serovar kurstaki str. T03a001*	7	3	4
GCF_000688795	*Bacillus thuringiensis serovar kurstaki str. YBT-1520*	6	5	1
GCF_000747545	*Bacillus thuringiensis serovar kurstaki str. YBT-1520*	3	1	0
GCF_000161595	*Bacillus thuringiensis serovar monterrey BGSC 4AJ1*	8	5	8
GCF_000940785	*Bacillus thuringiensis serovar morrisoni*	7	4	3

GCF_000161555	*Bacillus thuringiensis serovar pakistani str. T13001*	6	5	6
GCF_000161655	*Bacillus thuringiensis serovar pondicheriensis BGSC 4BA1*	7	7	6
GCF_000161695	*Bacillus thuringiensis serovar pulsiensis BGSC 4CC1*	11	6	8
GCF_000341665	*Bacillus thuringiensis serovar thuringiensis str. IS5056*	1	1	0
GCF_000161515	*Bacillus thuringiensis serovar thuringiensis str. T01001*	6	6	2
GCF_001548175	*Bacillus thuringiensis serovar tolworthi*	7	7	6
GCF_000015065	*Bacillus thuringiensis str. Al Hakam*	4	2	7
GCF_018885365	*Bacillus thuringiensis Strain: GR007*	0	0	0
GCF_000497525	*Bacillus thuringiensis YBT-1518*	6	3	6
GCF_016605985	*Bacillus toyonensis*	3	2	3
GCF_018739605	*Bacillus toyonensis Strain: BPN45/4*	1	1	2
GCF_018741125	*Bacillus toyonensis Strain: JAS03/3*	4	2	2
GCF_018741545	*Bacillus toyonensis Strain: JAS22/1*	5	3	1
GCF_018741525	*Bacillus toyonensis Strain: JAS13/1*	8	4	6
GCF_000496285	*Bacillus toyonensis BCT-7112*	0	2	0
GCF_018741625	*Bacillus toyonensis Strain: JAS411*	0	0	0
GCF_016026855	*Bacillus tropicus*	0	0	0
GCF_016027575	*Bacillus tropicus*	0	0	0
GCF_011040455	*Bacillus tropicus*	3	1	6
GCF_013331815	*Bacillus tropicus*	0	0	0
GCF_018141085	*Bacillus tropicus Strain: LM1212-DB*	0	0	0
GCF_003667885	*Bacillus vallismortis*	9	7	8

GCF_004116955	*Bacillus vallismortis*	10	5	6
GCF_002113805	*Bacillus vallismortis*	0	1	0
GCF_000875875	*Bacillus velezensis*	12	12	8
GCF_014358035	*Bacillus velezensis*	0	0	0
GCF_002290045	*Bacillus velezensis*	17	15	8
GCF_006489235	*Bacillus velezensis*	4	4	0
GCF_014495825	*Bacillus velezensis*	8	4	4
GCF_006489255	*Bacillus velezensis*	0	0	0
GCF_006489275	*Bacillus velezensis*	3	4	1
GCF_014622705	*Bacillus velezensis*	0	0	0
GCF_002310315	*Bacillus velezensis*	0	1	0
GCF_002327165	*Bacillus velezensis*	2	1	0
GCF_000973485	*Bacillus velezensis*	3	3	0
GCF_014706595	*Bacillus velezensis*	11	14	10
GCF_014764325	*Bacillus velezensis*	5	4	2
GCF_006965525	*Bacillus velezensis*	7	5	1
GCF_014841075	*Bacillus velezensis*	0	0	0
GCF_000987825	*Bacillus velezensis*	12	13	8
GCF_006974185	*Bacillus velezensis*	0	0	1
GCF_000988345	*Bacillus velezensis*	5	8	3
GCF_002556565	*Bacillus velezensis*	11	13	11
GCF_002688525	*Bacillus velezensis*	4	0	1

GCF_001023 595	*Bacillus velezensis*	10	13	9
GCF_002741 705	*Bacillus velezensis*	11	13	8
GCF_014961 825	*Bacillus velezensis*	0	0	0
GCF_002761 535	*Bacillus velezensis*	14	14	8
GCF_002764 075	*Bacillus velezensis*	7	3	4
GCF_015239 615	*Bacillus velezensis*	2	6	2
GCF_002796 465	*Bacillus velezensis*	14	13	9
GCF_015277 495	*Bacillus velezensis*	2	2	1
GCF_015291 865	*Bacillus velezensis*	11	12	5
GCF_008244 925	*Bacillus velezensis*	12	11	6
GCF_002844 125	*Bacillus velezensis*	1	2	0
GCF_002845 365	*Bacillus velezensis*	6	12	8
GCF_002850 475	*Bacillus velezensis*	13	15	8
GCF_008367 895	*Bacillus velezensis*	2	0	0
GCF_015732 475	*Bacillus velezensis*	11	18	8
GCF_002893 845	*Bacillus velezensis*	7	5	4
GCF_002944 585	*Bacillus velezensis*	9	10	7
GCF_008727 675	*Bacillus velezensis*	8	12	7
GCF_008802 875	*Bacillus velezensis*	0	0	0
GCF_001536 925	*Bacillus velezensis*	8	15	9
GCF_002968 415	*Bacillus velezensis*	6	5	2
GCF_016065 635	*Bacillus velezensis*	0	1	0

GCF_002993245	*Bacillus velezensis*	10	14	10
GCF_009362235	*Bacillus velezensis*	0	0	0
GCF_003045165	*Bacillus velezensis*	0	0	0
GCF_016406185	*Bacillus velezensis*	11	14	7
GCF_001593395	*Bacillus velezensis*	12	14	5
GCF_003047005	*Bacillus velezensis*	0	1	1
GCF_016415725	*Bacillus velezensis*	13	12	10
GCF_003047025	*Bacillus velezensis*	0	0	0
GCF_003047045	*Bacillus velezensis*	0	1	1
GCF_016766595	*Bacillus velezensis*	13	12	5
GCF_003071465	*Bacillus velezensis*	4	4	1
GCF_003073255	*Bacillus velezensis*	15	16	11
GCF_003073455	*Bacillus velezensis*	0	0	0
GCF_003076535	*Bacillus velezensis*	12	9	11
GCF_016801735	*Bacillus velezensis*	0	0	0
GCF_016834435	*Bacillus velezensis*	8	7	2
GCF_016859395	*Bacillus velezensis*	0	2	1
GCF_016888865	*Bacillus velezensis*	0	0	0
GCF_003150855	*Bacillus velezensis*	1	7	3
GCF_009663035	*Bacillus velezensis*	11	13	13
GCF_016904925	*Bacillus velezensis*	15	15	10
GCF_009664145	*Bacillus velezensis*	5	6	2

GCF_003184945	*Bacillus velezensis*	14	14	9
GCF_003265265	*Bacillus velezensis*	2	1	0
GCF_003285085	*Bacillus velezensis*	3	1	0
GCF_009738165	*Bacillus velezensis*	10	15	9
GCF_003390415	*Bacillus velezensis*	9	17	8
GCF_001685645	*Bacillus velezensis*	12	12	12
GCF_009789615	*Bacillus velezensis*	11	11	5
GCF_001687745	*Bacillus velezensis*	4	2	0
GCF_003431885	*Bacillus velezensis*	0	0	0
GCF_003432425	*Bacillus velezensis*	9	9	8
GCF_003441635	*Bacillus velezensis*	13	18	5
GCF_009834085	*Bacillus velezensis*	7	8	6
GCF_003546955	*Bacillus velezensis*	10	11	9
GCF_009856975	*Bacillus velezensis*	9	11	10
GCF_003555525	*Bacillus velezensis*	0	0	0
GCF_001723585	*Bacillus velezensis*	9	13	7
GCF_003573875	*Bacillus velezensis*	11	13	11
GCF_009913295	*Bacillus velezensis*	14	11	10
GCF_003612755	*Bacillus velezensis*	0	0	0
GCF_009913315	*Bacillus velezensis*	9	9	6
GCF_001752685	*Bacillus velezensis*	12	15	10
GCF_009913335	*Bacillus velezensis*	0	0	0

GCF_009913 355	*Bacillus velezensis*	7	9	3
GCF_009913 375	*Bacillus velezensis*	1	1	0
GCF_001854 345	*Bacillus velezensis*	16	13	9
GCF_009913 475	*Bacillus velezensis*	0	0	0
GCF_009913 495	*Bacillus velezensis*	0	0	0
GCF_001857 985	*Bacillus velezensis*	11	12	10
GCF_009914 135	*Bacillus velezensis*	14	10	9
GCF_003667 745	*Bacillus velezensis*	13	11	8
GCF_009914 175	*Bacillus velezensis*	8	12	5
GCF_009931 675	*Bacillus velezensis*	15	15	8
GCF_003721 475	*Bacillus velezensis*	15	12	8
GCF_003854 915	*Bacillus velezensis*	10	12	8
GCF_003855 275	*Bacillus velezensis*	4	3	2
GCF_003860 445	*Bacillus velezensis*	3	3	2
GCF_011032 725	*Bacillus velezensis*	1	1	0
GCF_011100 445	*Bacillus velezensis*	4	0	0
GCF_011769 925	*Bacillus velezensis*	0	0	0
GCF_002005 345	*Bacillus velezensis*	18	13	10
GCF_011784 665	*Bacillus velezensis*	9	11	5
GCF_012226 065	*Bacillus velezensis*	12	14	10
GCF_002057 535	*Bacillus velezensis*	0	0	0
GCF_002072 695	*Bacillus velezensis*	0	1	0

GCF_004101805	*Bacillus velezensis*	16	10	7
GCF_012275165	*Bacillus velezensis*	1	2	0
GCF_012647845	*Bacillus velezensis*	6	4	0
GCF_002105595	*Bacillus velezensis*	9	12	8
GCF_000769555	*Bacillus velezensis*	4	3	0
GCF_002117165	*Bacillus velezensis*	0	0	0
GCF_004119575	*Bacillus velezensis*	8	7	5
GCF_013122275	*Bacillus velezensis*	0	0	0
GCF_013201135	*Bacillus velezensis*	0	0	0
GCF_013201465	*Bacillus velezensis*	16	13	8
GCF_004119735	*Bacillus velezensis*	4	2	1
GCF_000815275	*Bacillus velezensis*	11	16	13
GCF_002192235	*Bacillus velezensis*	0	0	0
GCF_004119855	*Bacillus velezensis*	0	0	0
GCF_004135455	*Bacillus velezensis*	12	14	12
GCF_002205715	*Bacillus velezensis*	11	12	10
GCF_004329055	*Bacillus velezensis*	6	4	1
GCF_004331895	*Bacillus velezensis*	12	14	8
GCF_004337655	*Bacillus velezensis*	6	4	1
GCF_013348705	*Bacillus velezensis*	1	0	0
GCF_013367755	*Bacillus velezensis*	0	0	0
GCF_002216755	*Bacillus velezensis*	5	6	0

GCF_004799565	*Bacillus velezensis*	10	13	8
GCF_013401395	*Bacillus velezensis*	12	12	8
GCF_013694265	*Bacillus velezensis*	10	12	11
GCF_002237515	*Bacillus velezensis*	8	11	7
GCF_013701805	*Bacillus velezensis*	0	0	0
GCF_002238395	*Bacillus velezensis*	8	14	3
GCF_013875015	*Bacillus velezensis*	10	8	5
GCF_002243325	*Bacillus velezensis*	11	15	10
GCF_005696395	*Bacillus velezensis*	10	11	12
GCF_000833005	*Bacillus velezensis*	10	15	9
GCF_014048285	*Bacillus velezensis*	11	13	7
GCF_014048385	*Bacillus velezensis*	9	13	7
GCF_014117505	*Bacillus velezensis*	22	24	11
GCF_014180705	*Bacillus velezensis*	6	7	4
GCF_014211995	*Bacillus velezensis*	0	0	0
GCF_017347625	*Bacillus velezensis Strain: AD-3*	12	7	7
GCF_017742975	*Bacillus velezensis Strain: AD8*	10	15	9
GCF_019222745	*Bacillus velezensis Strain: BS-G1*	0	0	0
GCF_009734085	*Bacillus velezensis Strain: GA1*	1	2	0
GCF_019334525	*Bacillus velezensis Strain: LABIM44*	7	12	10
GCF_019285615	*Bacillus velezensis Strain: LBY-1*	10	11	4
GCF_018415955	*Bacillus velezensis Strain: VTX20*	9	13	6

GCF_017948225	*Bacillus velezensis Strain: VY03*	9	12	3
GCF_019464595	*Bacillus velezensis Strain: WB*	0	1	0
GCF_018223705	*Bacillus velezensis Strain: Y816*	12	11	8
GCF_019163475	*Bacillus velezensis Strain: YYC*	2	1	0
GCF_019551675	*Bacillus velezensis Strain: 19573-3*	0	0	0
GCF_018866365	*Bacillus velezensis Strain: GMEKP1*	1	3	1
GCF_018775385	*Bacillus velezensis Strain: J17-4*	0	1	0
GCF_018406485	*Bacillus velezensis Strain: KOF112*	1	0	0
GCF_018732185	*Bacillus velezensis Strain: NZ4*	14	12	7
GCF_018771705	*Bacillus velezensis Strain: US1*	0	0	0
GCF_018629015	*Bacillus velezensis Strain: VCN56*	11	8	2
GCF_018398955	*Bacillus velezensis Strain: VTX9*	10	17	8
GCF_000319475	*Bacillus velezensis AS43.3*	11	14	11
GCF_000283695	*Bacillus velezensis CAU B946*	9	15	11
GCF_000015785	*Bacillus velezensis FZB42*	14	16	12
GCF_000493375	*Bacillus velezensis NAU-B3*	12	12	9
GCF_000973585	*Bacillus velezensis NJN-6*	7	4	0
GCF_000685725	*Bacillus velezensis SQR9*	11	17	10
GCF_019449395	*Bacillus velezensis Strain: GS-1*	4	6	2
GCF_018278845	*Bacillus velezensis Strain: JK19*	5	3	0
GCF_018771665	*Bacillus velezensis Strain: Q12*	9	6	1
GCF_000583065	*Bacillus velezensis TrigoCor1448*	12	15	9

GCF_000455 565	*Bacillus velezensis UCMB5033*	12	15	11
GCF_000341 875	*Bacillus velezensis UCMB5036*	12	15	11
GCF_000455 585	*Bacillus velezensis UCMB5113*	13	13	10
GCF_000284 395	*Bacillus velezensis YAU B9601-Y2*	4	2	0
GCF_001889 165	*Bacillus weihaiensis*	4	2	3
GCF_000160 955	*Bacillus wiedmannii*	5	1	9
GCF_008807 735	*Bacillus wiedmannii*	6	2	9
GCF_018741 285	*Bacillus wiedmannii Strain: JAS07/5*	8	5	10
GCF_018741 405	*Bacillus wiedmannii Strain: JAS08/1*	3	1	9
GCF_018741 585	*Bacillus wiedmannii Strain: JAS28/2*	9	6	8
GCF_001857 925	*Bacillus xiamenensis*	4	3	8
